# Ligand‐Engineered Metal–Organic Frameworks of 3D Infinite Trinuclear Zinc Units for Photocatalytic Monooxygenation of Sulfenamides

**DOI:** 10.1002/advs.202506037

**Published:** 2025-06-05

**Authors:** Xinglei He, Chunlong Yu, Fengtao Zhang, Chenxu Gong, Jingheng Li, Ding‐Bo Zeng, Bin Zhao, Xiong Chen, Ke‐Yin Ye

**Affiliations:** ^1^ Key Laboratory of Molecule Synthesis and Function Discovery (Fujian Province University) College of Chemistry Fuzhou University Fuzhou 350108 China; ^2^ School of Chemistry and Chemical Engineering Henan Normal University Xinxiang 453007 China

**Keywords:** aerobic photooxidation, atomic‐scale regulation, CO_2_ cycloaddition, infinite trinuclear Zn units, metal–organic frameworks

## Abstract

Metal–organic frameworks (MOFs) composed of infinite metal units exhibit enhanced electron transport and charge migration capabilities compared to discrete metal units. Herein, three underdeveloped isomorphic MOFs featuring 3D infinite zinc units are designed and synthesized. The Lewis acidity and photocatalytic activity of these MOFs are fine‐tuned through atomic‐level engineering of nitrogen atoms of ligands and the resultant change of charge transfer modes. These MOFs are promising catalysts in the photocatalytic monooxygenation of sulfenamides with molecular oxygen. Mechanistic investigations suggest that the uneven charge distribution and large dipole moment at the pyridine center of 1‐PTB and the infinite Zn unit frameworks of degenerate energy levels are key to its excellent photocatalytic activity.

## Graphical abstract

The rarely investigated metal–organic frameworks (MOFs) of 3D infinite trinuclear zinc units are designed and synthesized. Through controllable programming of ligands, their Lewis acidity and photoactivity are fine‐tuned to enable these MOFs as promising catalysts for the cycloaddition of CO_2_ and photocatalytic monooxygenation of sulfenamides and sulfides.

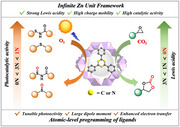

## 1 Introduction

**Scheme 1:**
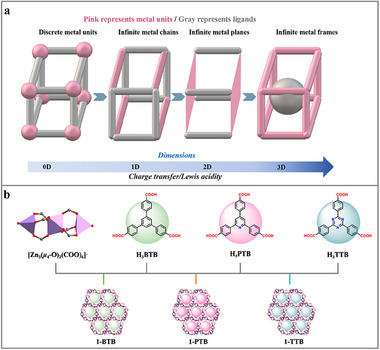
a) Metal units in MOFs of various dimensions. b) Synthesis of 1‐BTB, 1‐PTB, and 1‐TTB.

Metal–organic frameworks (MOFs) are crystalline porous materials with well‐defined structures through the assembly of diverse metal ions/clusters and bridging ligands.^[^ ^1^, ^2^, ^3^ ^]^ Regarding the dimension of metal units, most MOFs contain discrete, 0D metal nodes separated by ligands. This feature explains their low charge mobility because of insufficient orbital overlap between bridging ligands and their innate porous structures.^[^ ^4^ ^]^ By contrast, MOFs with infinite 1D metal chains, 2D metal planes, and even 3D metal frames are sparely investigated and mostly concentrated on titanium clusters (**Scheme** **1**a).^[^ ^5^ ^]^ One potential benefit of infinite metal units is that degeneration of the molecular orbital renders much‐dispersed and abundant electrons across the frameworks to facilitate charge carrier migration and thus improve catalytic activity.^[^ ^5^, ^6^ ^]^

Compared with various types of photocatalysts, such as covalent–organic frameworks,^[^ ^7^, ^8^ ^]^ metal oxide semiconductor,^[^ ^9^, ^10^ ^]^ and carbon nitride,^[^ ^11^, ^12^ ^]^ MOFs^[^ ^6^, ^13^ ^]^ feature the simple modifiability to fine‐tune their charge separation and transfer efficiency via metal and ligand synergy.^[^ ^6^, ^14^, ^15^ ^]^ The structural programmability of organic ligands allows the rational design of framework architectures with well‐defined catalytic centers, enabling accurate control over the spatial arrangement of active sites and the physicochemical properties of materials.^[^ ^14^, ^15^, ^16^, ^17^ ^]^ For instance, incorporating nitrogen atoms enables the precise modulation of charge distribution, light‐harvesting capabilities, and bandgap engineering in MOFs. This helps to improve their optoelectronic performance, modulate Lewis acid‐base characters, and enhance charge transfer efficiency.^[^ ^17^, ^18^, ^19^, ^20^ ^]^

Zn‐based MOFs have been widely recognized as promising heterogeneous catalysts of exceptional catalytic activity because of their low cost and toxicity, high crystallinity, and facile preparation.^[^ ^21^ ^]^ Unlike the 1D chains and 2D planes of MOFs containing infinite Ti clusters,^[^ ^5^ ^]^ the weak coordination formed by the soft Zn^2+^ ion and hard carboxylate provides ample opportunities for the ion exchange between Zn^2+^ and diverse metal ions for the late‐stage construction of functional MOFs containing infinite metal units.^[^ ^22^, ^23^ ^]^ Herein, we have designed and synthesized three 3D MOFs featured infinite trinuclear [Zn_3_(*μ*_*4*_‐O)_2_(COO)_6_]^–^ units [1‐BTB, 1‐PTB, and 1‐TTB; H_3_BTB = 4,4′,4′′‐benzene‐1,3,5‐triyl‐tris(benzoic acid); H_3_PTB = 4,4′,4′′‐pyridine‐1,3,5‐triyl‐tris(benzoic acid); H_3_TTB = 4,4′,4′′‐triazine‐1,3,5‐triyl‐tris(benzoic acid)] (Scheme 1b). To the best of our knowledge, none of the MOFs with 3D infinite metal frames are known yet. Unlike polyoxometalates,^[^ ^24^, ^25^ ^]^ all organic ligands of these MOFs are trapped within the cages of the infinite metal frameworks and thus provide ample opportunities to fine‐tune their structures. In this context, we first demonstrated that all infinite trinuclear Zn units exhibit enhanced Lewis acidity than those discrete ones and efficiently catalyze the cycloaddition of CO_2_ and styryl epoxide with high turnover number (TON) and turnover frequency (TOF). Through the atomic‐scale regulation of nitrogen atoms of ligands, a distinctive metal‐to‐ligand charge transfer (MLCT) is operative for their enhanced photocatalytic activity with molecular oxygen. These MOFs are ideal photocatalysts for the green and sustainable monooxygenation of a wide spectrum of sulfenamides with molecular oxygen.

## 2 Results and Discussions

**Figure 1:**
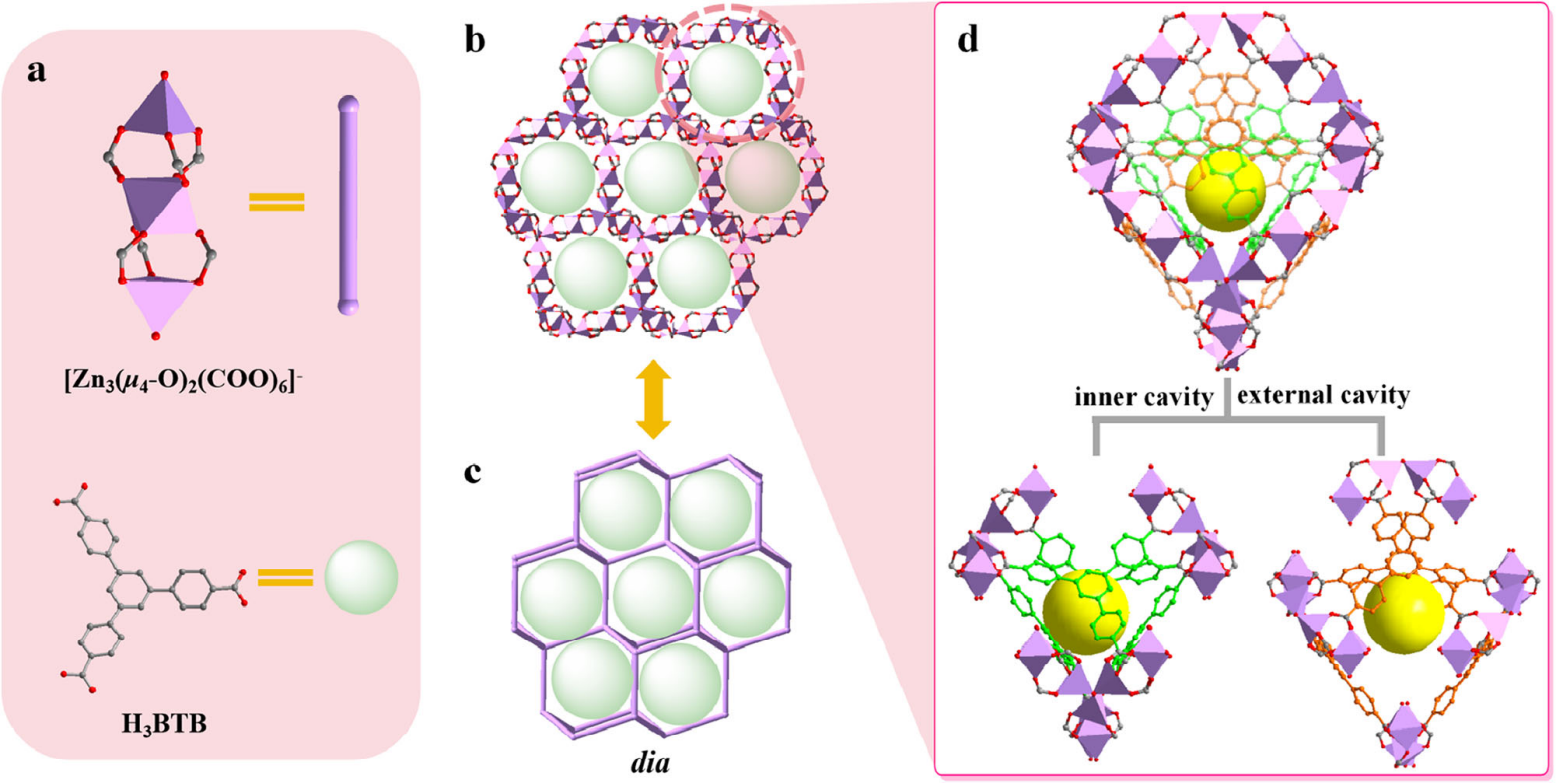
a) Structures of the trinuclear Zn unit and H_3_BTB. b,c) The 3D framework (b) and topology (c) of the infinite trinuclear Zn units. d) The cages of 1‐BTB.

We first synthesized three isomorphic MOFs assembled by 3D infinite trinuclear Zn units and tridentate carboxylic acid ligands, i.e., 1‐BTB, 1‐PTB, and 1‐TTB, respectively (Scheme 1b). Their isomorphic structures were confirmed by simulated powder X‐ray diffraction (PXRD) patterns (Figure S2a, Supporting Information). In addition, the single crystal X‐ray diffraction of them (SC‐XRD, Table S1, Supporting Information) reveals a *cubic* crystal system with an *Fd‐3* space group. The asymmetric unit consists of 1/3 of the ligand, two Zn atoms (Zn1 and Zn2), and a *μ*_*4*_‐O from H_2_O (O1, Figure S3a–c, Supporting Information). Zn1 forms an octahedron configuration with six oxygen atoms from six carboxylic acids (Figure S3d, Supporting Information). Zn2 forms a tetrahedron configuration with three oxygen atoms from three carboxylic acids and one *μ*_*4*_‐O atom from H_2_O (Figure S3e, Supporting Information). Based on one Zn1 and two Zn2, a trinuclear Zn unit ([Zn_3_(*μ*_*4*_‐O)_2_(COO)_6_]^–^) coordinated with carboxylic acids was formed (**Figure** **1**a; Figure S3f, Supporting Information). Four trinuclear Zn units are further connected by one *μ*_*4*_‐O atom to form a tetrahedron structure (Figure S3g,h, Supporting Information) and infinitely expand (Figure 1b). Overall, this 3D framework is anionic, balanced by the Me_2_NH_2_^+^ cation or proton from H_2_O.

The resultant infinite Zn unit framework is a typical dia topology. This assembly traps all organic ligands within the cages of the framework (Figure 1b,c). The six carboxylic acids in each cage form a double‐layered cavity structure and lock each window (Figure 1d). Therefore, this dense structure results in a negligible specific surface area but profoundly enhanced structural stability (Figure S4, Supporting Information).^[^ ^23^, ^26^ ^]^ According to the PLATON analysis, the effective free voids of 1‐BTB, 1‐PTB, and 1‐TTB of the crystal volume are 5.1%, 8.2%, and 7.4%, respectively. These results are consistent with the Connolly surface analysis (Figure S5, Supporting Information).

Thermal gravimetric analysis (TGA) showed that weight losses at 100, 100–400, and 400 °C are attributed to evaporation of free water molecules, breakage of coordination bonds, and structural collapse, respectively (Figure S6a, Supporting Information). However, no decomposition of these MOFs was observed at 300 °C (Figure S6b–d, Supporting Information). Moreover, their tolerance across a broad pH range and diverse solvents further underscores their chemical stability (Figure S7, Supporting Information). The scanning electron microscopy (SEM) images reveal that they are regularly polyhedron crystals (Figure S8a–c, Supporting Information). Energy dispersive spectrometer (EDS) mapping images and spectra show the uniform distribution of each element (Figure S8d–f, Supporting Information). The presence of N atoms in 1‐PTB and 1‐TTB is consistent with the results of elemental analysis and X‐ray photoelectron spectroscopy (XPS) spectra (Figures S8g–i and S9, Supporting Information).

**Figure 2:**
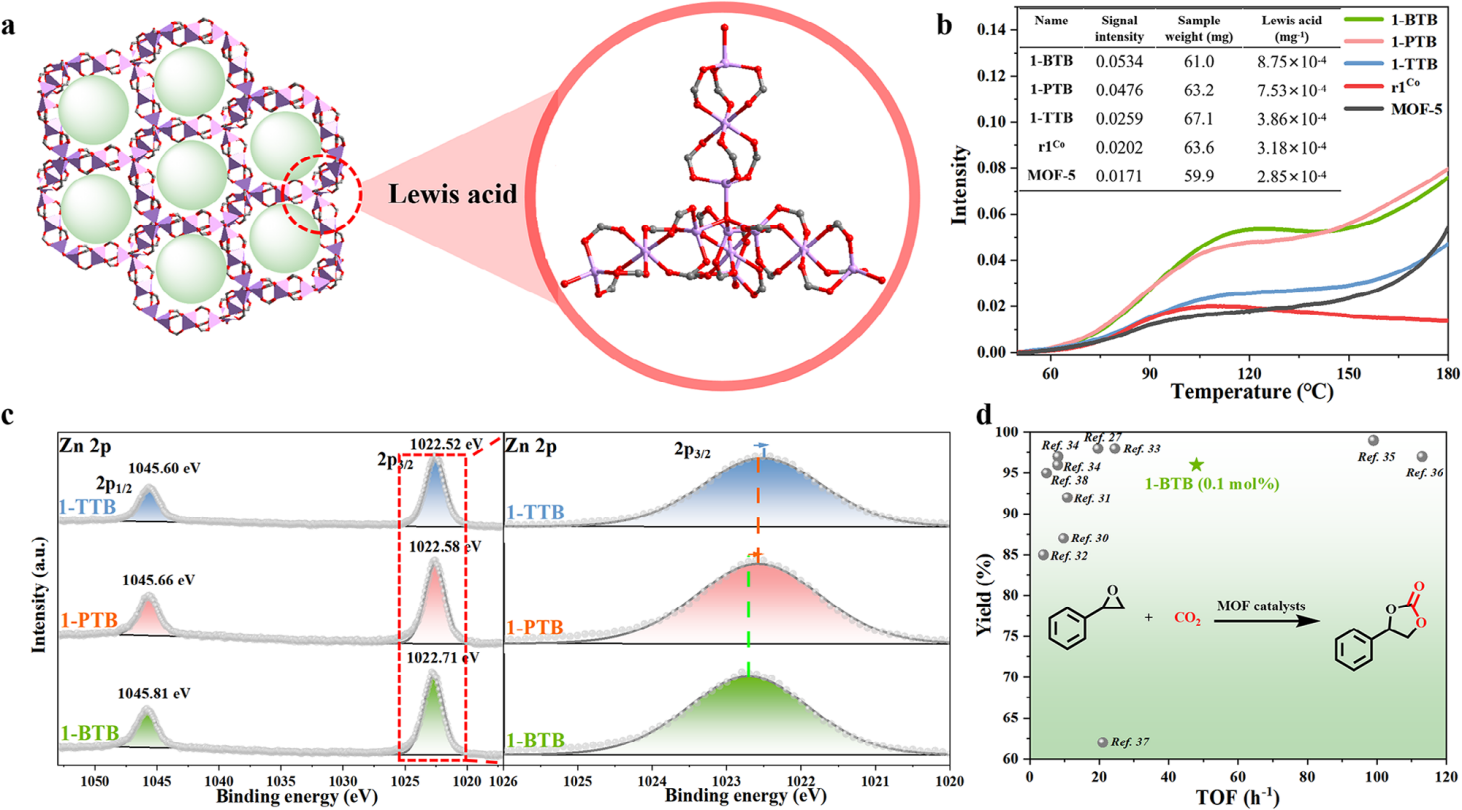
a) The local Lewis acid sites of 1‐BTB. b) TPD‐NH_3_ profiles. c) The Zn 2p XPS spectra. d) MOFs‐catalyzed cycloaddition of CO_2_ and styrene epoxide.

According to the temperature‐programmed desorption (TPD) of ammonia (NH_3_), all MOFs containing infinite trinuclear Zn units (1‐BTB, 1‐PTB, and 1‐TTB) display higher Lewis acidity than the discrete ones (r1Co^[^ ^27^ ^]^ and MOF‐5^[^ ^28^ ^]^) (**Figure** **2**a,b). Specifically, 1‐BTB exhibits higher Lewis acidity, which is consistent with the XPS spectra where Zn atoms in 1‐BTB exhibit the highest binding energy (Figure 2c). We hypothesize that the electron‐donating nature of N atoms in 1‐PTB and 1‐TTB diminishes their Lewis acidity.^[^ ^29^ ^]^

We chose the cycloaddition of CO_2_ and styrene epoxide as the benchmark reaction to investigate the Lewis acidity of these MOFs further. After extensive reaction optimizations (see Figure S10, Supporting Information), 0.1 mol% of 1‐BTB displayed the highest catalytic efficiency to give the anticipated carbonate in 96% yield. The turnover number (TON) and turnover frequency (TOF) are 960 and 48.5 h^−1^, respectively, outperforming most reported MOF catalysts (Figure 2d; Table S3, Supporting Information).^[^ ^27^, ^30^, ^31^, ^32^, ^33^, ^34^, ^35^, ^36^, ^37^, ^38^ ^]^ Notably, this cycloaddition reaction accommodates various epoxides (Scheme S1, Supporting Information). Remarkably, the catalyst was easily recycled while maintaining high catalytic activity (Figure S11, Supporting Information). By contrast, the analogous discrete trinuclear Zn unit was less catalytically competent (63% yield, Figure S10c, Supporting Information),^[^ ^39^ ^]^ substantiating the enhanced Lewis acidity of the infinite trinuclear Zn units (see Figure S12, Supporting Information, for the possible mechanism).

**Figure 3:**
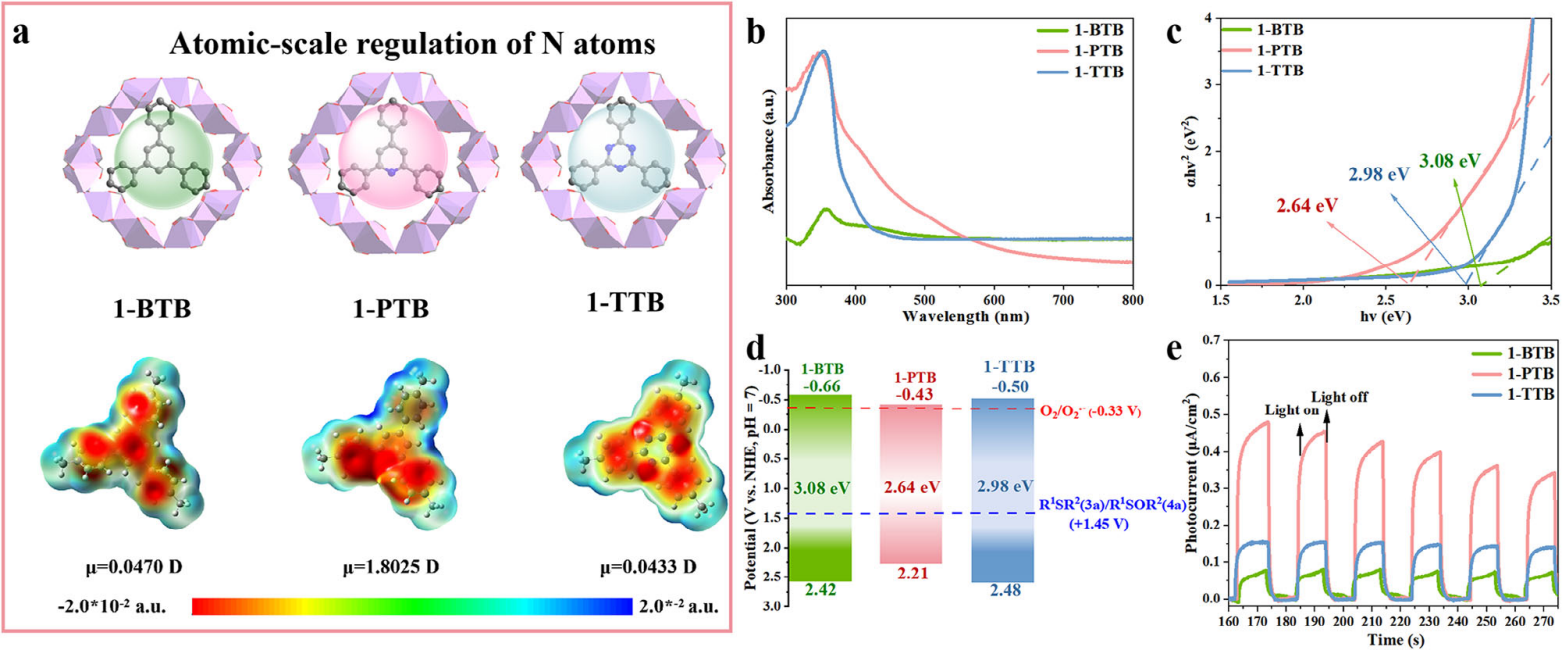
a) Regulation of the N atoms and the ESP mapping (red: negative potential; blue: positive potential) with dipole moments of carboxylic acids. b) UV–vis DRS spectra. c) Tauc plots for band gap calculation. d) Band structures. e) Transient photocurrent tests.

Leveraging the precise regulation of the N atoms in the ligands, the photoactivities of three MOFs were systematically evaluated (**Figure** **3**a). The ultraviolet–visible diffuse reflectance spectroscopy (UV–vis DRS) reveals a strong and broad absorption in the range of 300–550 nm for 1‐PTB (Figure 3b), primarily attributable to carboxylate ligands and the charge transfer within the frameworks (Figure S13, Supporting Information).^[^ ^6^, ^14^, ^40^, ^41^ ^]^ The Tauc plots were derived via the Kubelka–Munk function, thereby inferring the band gaps (*E*_g_) of 1‐BTB, 1‐PTB, and 1‐TTB to be 3.08, 2.64, and 2.98 eV, respectively (Figure 3c). The positive slope of the Mott–Schottky plots proves that these MOFs are *n*‐type semiconductors,^[^ ^42^, ^43^ ^]^ in which the conduction band (*E*_CB_) potentials of 1‐BTB, 1‐PTB, and 1‐TTB are −0.66, −0.43, and −0.50 V (vs normal hydrogen electrode at pH 7), respectively (Figure S14a–c, Supporting Information). Furthermore, their calculated valence band (*E*_VB_) potentials are 2.42, 2.21, and 2.48 V, which are consistent with the valence band X‐ray photoelectron spectroscopy (VB‐XPS) tests (Figure S14d, Supporting Information). Therefore, the band structures of MOFs suggest their potential to reduce O_2_ to O_2_‐ (E_O2_, −0.33 V)^[^ ^44^ ^]^ and oxidize sulfenamides (E_3a_, +1.42 V)^[^ ^45^ ^]^ (Figure 3d), underscoring their photocatalytic promise.

Transient photocurrent tests showed that 1‐PTB exhibited the highest photocurrent density among the three variants, indicative of its superior charge separation and transfer efficiency (Figure 3e).^[^ ^14^, ^44^ ^]^ Electrochemical impedance spectroscopy (EIS) confirms the lowest interfacial charge transfer resistance of 1‐PTB, which facilitates charge migration (Figure S15a, Supporting Information).^[^ ^14^, ^46^ ^]^ In addition, the linear sweep voltammetry (LSV) curves further substantiate 1‐PTB's exceptional photoelectrochemical performance, displaying the highest current density among the three MOFs (Figure S15b, Supporting Information).^[^ ^47^ ^]^

We further analyzed the differences in photocatalytic activities by building reasonable molecular models and using rationalized density functional theory (DFT) calculations. The highest occupied molecular orbital (HOMO) and the lowest unoccupied molecular orbital (LUMO) of the trinuclear Zn unit and molecular fragments of three carboxylate ligands show that the trinuclear Zn unit can form staggered frontier orbitals with BTB and PTB (Figure S17, Supporting Information).^[^ ^47^ ^]^ In particular, the close LUMO energy levels between the trinuclear Zn unit and PTB are thermodynamically conducive for charge transfer (Figure S17, Supporting Information).^[^ ^6^ ^]^

Furthermore, the electrostatic potential (ESP) maps were evaluated by Multiwfn.^[^ ^48^, ^49^ ^]^ Figure 3a shows charges in BTB and TTB are evenly distributed across the benzene or triazine rings, respectively. By contrast, the charges in PTB are concentrated on the N atom in pyridine, which creates a considerable dipole moment (1.8025 D) compared to BTB (0.0470 D) and TTB (0.0433 D). The enhanced dipole moment amplifies electric field polarization, thereby promoting efficient charge separation and electron transfer.^[^ ^19^ ^]^ The abundant electrons in the infinite Zn unit frame and uneven charge distribution result in the charge transfer path shift from metal‐to‐metal charge transfer (MMCT) in 1‐BTB and 1‐TTB to MLCT in 1‐PTB (Figure S18, Supporting Information). This change of charge transfer path is key for enhanced photoactivity as MLCT is more conducive to the separation of electron‐hole pairs than MMCT.^[^ ^50^, ^51^, ^52^ ^]^

Photoluminescence (PL) spectroscopies provided additional insights into electron transfer kinetics. The PL intensity of these MOFs likely originates from the ligands (Figure S16a,b, Supporting Information). Comparative PL spectra of the MOFs and their carboxylate ligands reveal a pronounced blue shift in 1‐PTB (from 470 to 394 nm) relative to 1‐BTB (from 393 to 384 nm) and 1‐TTB (from 389 to 386 nm), signifying efficient charge transfer from the trinuclear Zn unit to the PTB in 1‐PTB (Figure S16d–f, Supporting Information).^[^ ^50^ ^]^

**Figure 4:**
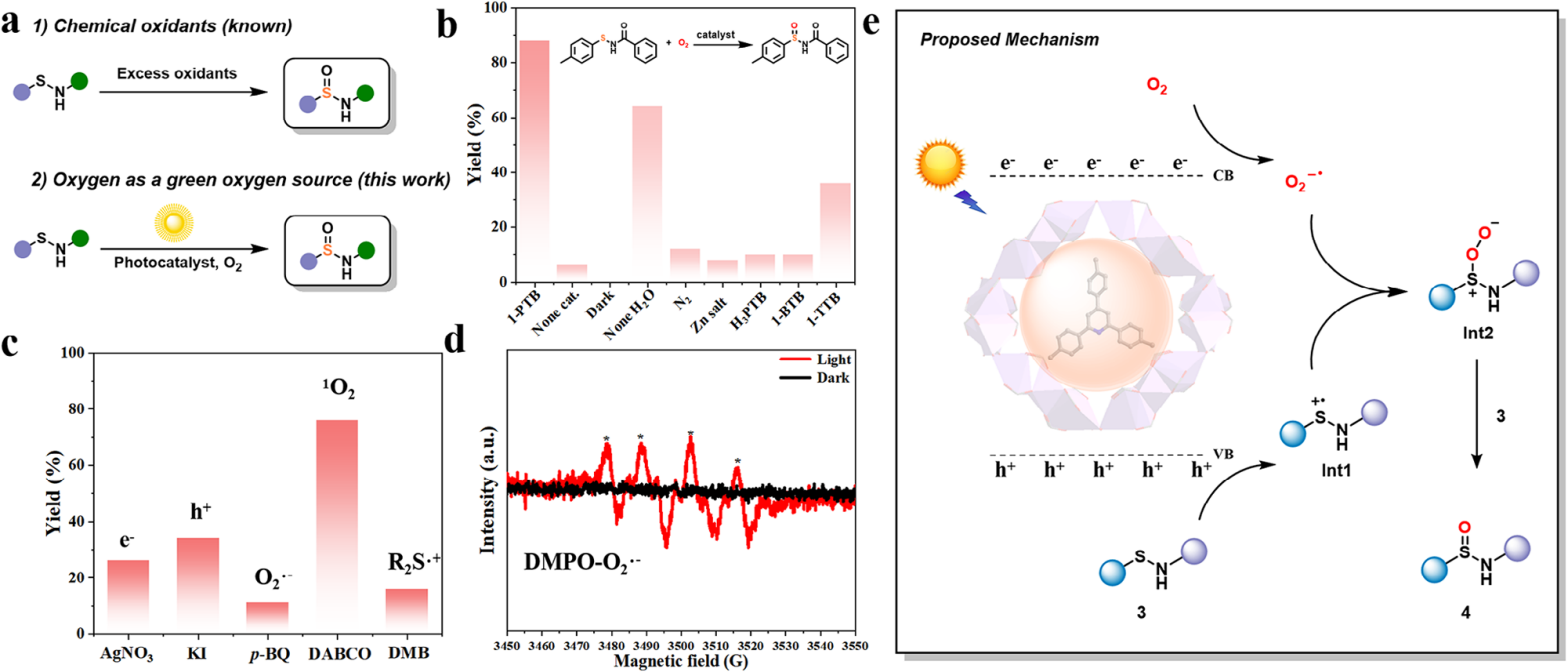
a) Chemical and photocatalytic monooxygenation of sulfenamides. b) Optimization of reaction conditions. c) Control experiments to probe potential active species. d) EPR spectra (in dark or under Xe lamp for 135 s) of 1‐PTB (1.0 mg L^−1^) in air‐saturated MeCN that contained 0.1 m DMPO. e) Possible mechanism of photocatalytic monooxygenation of sulfenamides.

Sulfinamides are widely spread in diverse biologically active molecules, drug intermediates, or ligands.^[^ ^53^, ^54^ ^]^ Photocatalytic monooxygenation of sulfenamides under aerobic conditions is a green and sustainable approach as it obviates excess oxidants but uses visible light and oxygen as the energy source and oxidant, respectively (**Figure** **4**a).^[^ ^53^, ^54^, ^55^, ^56^ ^]^ Photocatalytic monooxygenation of *N*‐(*p*‐tolylthio)benzamide was realized with 1‐PTB as the catalyst under a 420 nm LED lamp with an O_2_ balloon for 10 h at 25 °C in 88% yield, which is also consistent with photoactivity assessments for three MOFs (Figure 4b). Control experiments confirm the pivotal roles of 1‐PTB, O_2_, and light irradiation. Remarkably, Zn(NO_3_)_2_·6H_2_O, H_3_PTB, and 1‐BTB exhibited negligible photocatalytic activity. The yield decreased to 36% with 1‐TTB as the photocatalyst. Without water, the yield was reduced to 64%. This system can be amplified (4.5 mmol, 1.1 g) under optimal conditions to give the target product in 72% yield. The types and amounts of solvents, reaction time, and loading of the catalyst were further investigated to determine the optimal reaction conditions (Figure S19, Supporting Information). Investigation of the effects of light wavelengths revealed that the best activity was achieved under the irradiation of light of 420 nm (Figure S20, Supporting Information). 1‐PTB can be reused while maintaining good catalytic activity (Figure S21a, Supporting Information) and structural stability as confirmed by PXRD patterns and XPS spectra (Figure S21b–d, Supporting Information). Additionally, 1‐PTB is also suitable for aerobic photooxidation from sulfides into sulfoxides (Figure S22 and Scheme S2, Supporting Information). It is worth noting that due to the nonporous structure of MOFs, the reaction occurs on the surface of catalyst. However, unlike ZnO or TiO_2_, the ligand can fine‐tune the electron distribution and charge transport pathway.

To verify the possible active species, we carried out mechanistic investigations. The yield was profoundly reduced with the addition of AgNO_3_ (an electron scavenger) or KI (a hole scavenger), confirming the necessity of photoinduced e^−^ and h^+^ (Figure 4c). Similarly, the decreased yield with *p*‐benzoquinone (*p*‐BQ, an O_2_^•^^−^ scavenger) is indicative of photoinduced O_2_^•^^−^ (Figure 4c). Further, the successful capture of O_2_^•^ **^**−**^** by 5,5‐dimethyl‐1‐pyrroline N‐oxide (DMPO) was monitored by electron paramagnetic resonance (EPR) spectroscopy. As shown in Figure 4d, after light irradiation, a characteristic quadruple signal of DMPO‐O_2_^•^^−^ adduct appeared. Conversely, the presence of diazabicyclo[2.2.2]octane (DABCO) had no effect, and the radical trapping experiment with 2,2,6,6‐tetramethylpiperidine (TEMP) failed to detect ^1^O_2_, ruling out its involvement (Figure 4c; Figure S23, Supporting Information).^[^ ^57^ ^]^ Moreover, the reduced yield with 1,4‐dimethoxybenzene (DMB, a sulfur radical cation scavenger) substantiated the role of sulfur radical cations. Therefore, the photocatalytic‐generated e^−^, h^+^, O_2_^•^^−^, and sulfur radical cations are active species in this monooxygenation of sulfenamides.

A possible mechanism is illustrated in Figure 4e. 1‐PTB is excited under light irradiation, producing electrons (e^−^) and holes (h^+^). This charge transfer process facilitates both the reduction of dioxygen to the superoxide anion radical and the single‐electron oxidation of sulfenamide (3) to the corresponding radical cation (Int1), respectively. These radical intermediates then readily recombine to generate the peroxyl sulfenamide (Int2) to ultimately afford the anticipated sulfinamide (4) through the reaction with another equivalent of sulfenamide (3).^[^ ^58^ ^]^

**Scheme 2:**
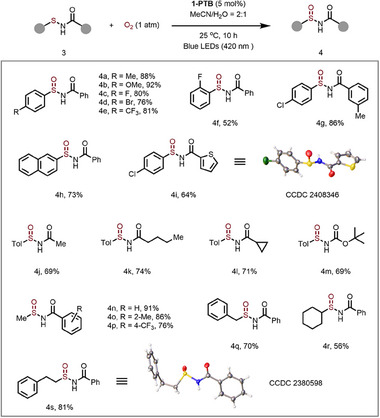
Substrate scope of sulfinamides. Reaction conditions: 3 (0.2 mmol), 1‐PTB (20 mg, 5 mol% based on trinuclear Zn unit), MeCN /H_2_O = 2:1, irradiated under blue LEDs (420 nm) under an O_2_ balloon at 25 °C for 10 h.

The substrate scope of this 1‐PTB‐catalyzed photocatalytic monooxygenation of sulfenamides to sulfinamides was delineated (**Scheme** **2**). Various *N*‐(arylthio)benzamides bearing electron‐donating, such as Me (4a, 4g) and MeO (4b), and electron‐withdrawing substituents, including F (4c, 4f), Br (4d), and CF_3_ (4e), were all well tolerated. The sulfinamide containing a thiophene moiety (4i) was obtained in 64% yield, and its chemical structure was confirmed by X‐ray diffraction analysis. Besides benzamides, alkyl carboxamides such as acetamide (4j), pentanamide (4k), cyclopropanecarboxamide (4l), and tert‐butylcarbamate (4m) were well tolerated. Sulfenamides derived from alkylthiols, including Me (4n‐4p), benzyl (4q), cyclohexyl (4r), and phenylethyl (4s, CCDC 2380598) were readily transformed into their corresponding sulfinamides in good to excellent yields. This photooxidation protocol was also applied to the monooxygenation of various sulfides to sulfoxides (see Scheme S2, Supporting Information).

## 3 Conclusion

In summary, three rarely investigated 3D MOFs of high Lewis acidity featuring infinite Zn unit frameworks were designed and synthesized. Using the cycloaddition of CO_2_ and styrene epoxide as the benchmark reaction, 0.1 mol% of 1‐BTB catalyst resulted in the formation of carbonate in excellent yield (96%) with high TON (990) and TOF (48.5 h^−1^). These MOFs also serve as promising photocatalysts as they provide an opportunity to regulate photocatalytic activity via atomic‐scale regulation of N atoms. The uneven charge distribution and large dipole moment at the pyridine center of 1‐PTB, together with the infinite Zn unit frameworks of degenerate energy levels, render it a competent photocatalyst. Furthermore, the unique MLCT pathway also explains its enhanced photocatalytic activity with molecular oxygen, which was exemplified by the green and sustainable photocatalytic monooxygenation of a wide spectrum of sulfenamides.

## Conflict of Interest

The authors declare no conflict of interest.

## Supporting information



Supporting Information

## Data Availability

Data that support the findings of this work are available from the corresponding author upon reasonable request.
