# Supplementary material for: Ligand‐Engineered Metal–Organic Frameworks of 3D Infinite Trinuclear Zinc Units for Photocatalytic Monooxygenation of Sulfenamides
Source: Adv Sci (Weinh). 2025 Jun 5;12(33):e06037. doi: 10.1002/advs.202506037 (PMC12412583; doi:10.1002/advs.202506037)
Supplement: Supplementary file 1 — Supporting Information [file ADVS-12-e06037-s001.pdf]

ADVANCED SCIENCE
Open Access

Supporting Information

for *Adv. Sci.*, DOI 10.1002/advs.202506037

Ligand-Engineered Metal–Organic Frameworks of 3D Infinite Trinuclear Zinc Units for Photocatalytic Monooxygenation of Sulfenamides

Xinglei He, Chunlong Yu, Fengtao Zhang, Chenxu Gong, Jingheng Li, Ding-Bo Zeng, Bin Zhao, Xiong Chen and Ke-Yin Ye**

Supporting Information

Ligand-Engineered Metal-Organic Frameworks of Three-Dimensional Infinite Trinuclear Zinc Units for Monooxygenation of Sulfenamides

*Xinglei He, ‡[a] Chunlong Yu, ‡[a] Fengtao Zhang, [a] Chenxu Gong, [a] Jingheng Li, [a] Ding-Bo Zeng, [a] Bin Zhao, [a] Xiong Chen *[a] and Ke-Yin Ye *[a,b]*

[a] Key Laboratory of Molecule Synthesis and Function Discovery (Fujian Province University), College of Chemistry, Fuzhou University, Fuzhou 350108, China.

[b] School of Chemistry and Chemical Engineering, Henan Normal University, Xinxiang 453007, China.

* Corresponding author, e-mail: chenxiong987@fzu.edu.cn; kyye@fzu.edu.cn.

‡ These authors contributed equally to this work.

Content

SI-1 Experimental section **3**

SI-2 Crystal structure description and characterization **10**

SI-3 Lewis acidity study of MOFs in the CO_2 cycloaddition **18**

SI-4 Optical performance research **24**

SI-5 DFT calculations **29**

SI-6 Photocatalytic monooxygenation of sulfenamides **31**

SI-7 Study on the reaction mechanism for photocatalytic monooxygenation ... **37**

SI-8 NMR data **38**

SI-9 References **75**

SI-1 Experimental section

Materials and Instrumentation

All reagents and solvents in this work are commercially available and without further purification. Acetonitrile (99.9%), *N*,*N*-Dimethylformamide (99.9%), *N*,*N*-Dimethylacetamide (99.5%), ethanol (≥95.0%), methanol (≥99.5%), 1,4-dioxane (≥99.5%), dimethyl sulfoxide (99.8%), ethyl acetate (99.5%), dichloromethane (≥99.5%), 4,4′,4″-benzene-1,3,5-triyl-tris(benzoic acid) (98.0%), 4,4′,4″-triazine-1,3,5-triyl-tris(benzoic acid) (98.0%), tetrabutylammonium bromide (99.0%), potassium iodide (≥99.5%), 1,4-benzoquinone (98.0%), 1,4-diazabicyclo[2.2.2]octane (99.0%), 1,4-dimethoxybenzene (99.0%), 5,5-dimethyl-1-pyrroline N-oxide (98.0%), 2,2,6,6-tetramethylpiperidine (98.0%), epoxides, sulfides, and reagents for synthesizing sulfenamide were purchased from Energy Chemical, Sun Chemical Technology (Shanghai) Co., Ltd. Zinc nitrate hexahydrate (≥99.0%), silver nitrate (≥99.8%), and toluene (99.5%) were purchased from Sinopharm Chemical Reagent Co., Ltd. 4,4′,4″-pyridine-1,3,5-triyl-tris(benzoic acid) (95.0 %) was purchased from Leyan Reagent, Shanghai Haohong Scientific Co., Ltd.

Elemental analyses were performed with a Vario EL Cube elemental analyzer. TG was carried out in an air atmosphere with a heating rate of 10 °C/min on a Mettler Toledo TG/DSC 3+. Single-crystal X-ray diffraction (SC-XRD) was tested on a Bruker D8 Venture using Mo Kα radiation. The empirical absorption correction was performed using the Crystal Clear program. The structure was solved by direct methods and refined on F^2 by the full-matrix least-squares technique using the SHELXL program package. Powder X-ray diffraction (PXRD) data was collected on an Ultima IV automated multipurpose X-ray diffractometer using Cu-Kα radiation. Elemental analysis (EA) was characterized by Vario EL Cube. The UV-visible absorption spectra were collected on a UV-2600 UV-vis spectrophotometer (Shimadzu) with a wave range of 200-1200 nm. Photoluminescence (PL) spectra were recorded on an Edinburgh FI/FSTCSPC 920 spectrophotometer. Electron paramagnetic resonance (EPR) measurements were performed on a Bruker model A300 spectrometer. Photocurrent performance was conducted with a BAS Epsilon Electrochemical System in a conventional three electrode cell, using a Pt plate as the counter electrode and an Ag/AgCl (3 M KCl) electrode as the reference electrode. The electrolyte was a 0.2 M Na_2SO_4 aqueous solution (pH = 7). The working electrode was prepared on an indium tin oxide (ITO) glass that was cleaned by sonication in ethanol for 30 min and dried at 353 K. The boundary of ITO glass was protected using scotch tape. 5 mg MOF sample was dispersed in 900 μL DMF and 100 μL Nafion by sonication to get a slurry. The slurry was

spread onto pretreated ITO glass. After air-drying, the Scotch tape was unstuck, and the uncoated part of the electrode was isolated with epoxy resin. X-ray photon electron spectroscopy (XPS) measurements were performed on a Thermo Scientific Nexsa X-ray photon electron spectrometer using Mg as the exciting source. The NMR experiments were carried out on Bruker AV-400 (400 MHz) or JEOL-500 (500 MHz) spectrometers. Chemical shifts were reported in ppm. ^{1}H NMR spectra were referenced to $CDCl_3$ (7.26 ppm) and ^{13}C NMR spectra were referenced to $CDCl_3$ (77.16 ppm). Peak multiplicities were designated by the following abbreviations: s, singlet; d, doublet; t, triplet; m, multiplet; brs, broad singlet and J, coupling constant in Hz. The N_2/CO_2 adsorption isotherms were recorded by using a Quantachrome EVO surface area and porosity analyzer. Before the adsorption measurement, the samples were activated at 120 °C under vacuum for 8 h. The acidity of the catalyst was determined by temperature programmed desorption of ammonia (TPD-NH_3), by using a Micromeritics AutoChem II 2920 adsorption instrument. Before NH_3 adsorption, the sample was activated at 120 °C for 1 h in He atmosphere. After adsorption of NH_3 at 50 °C for 1 h, the desorption step was performed from 50 to 400 °C at a heating rate of 10 °C/min. The surface morphologies and element distribution analyses were performed on a Regulus8230 Scanning Electron Microscope (SEM) with an Energy Dispersive Spectrometer (EDS). Before testing, the sample is sprayed with a thin layer of gold in a vacuum. HRMS were obtained on an Exactive Plus LC-MS (ESI) mass spectrometer with the use of a quadrupole analyzer or an Agilent 1290-6545XT mass spectrometer with a QTOF analyzer.

Synthesis of 1-BTB

A mixture of $Zn(NO_3)_2{\cdot}6H_2O$ (15.0 mg, 0.05 mmol) and 4,4′,4″-benzene-1,3,5-triyl-tris(benzoic acid) (H_3BTB, 13.2 mg, 0.03 mmol) was dissolved in a mixed solvent of N, N-dimethylacetamide (DMA) and H_2O (DMA/H_2O, 4.5 mL/1.5 mL) in a 10 mL screw cap vial with lid and heated at 90 °C for three days. After cooling to room temperature, colorless crystals were collected, washed with DMA and MeOH, and dried at 80 °C. Yield: 13.2 mg (82.5%). Elemental analysis data calculated (calcd.) for $C_{108}H_{48}O_{25}Zn_6$: C, 60.7; H, 2.2; O, 18.7%. Found: C, 58.1; H, 4.2; O, 21.3%. The CCDC number for **1-BTB** is 2302677.

Synthesis of 1-PTB

The synthesis process of **1-PTB** is the same as that of **1-BTB**, except that H_3BTB is replaced with 4,4′,4″-pyridine-1,3,5-triyl-tris(benzoic acid) (H_3PTB). Yield: 9.3 mg (57.6%). Elemental analysis data calculated (calcd.) for $C_{104}H_{56}N_4O_{25}Zn_6$: C, 58.0; H, 2.6; N, 2.6; O, 18.6 %. Found: C, 55.4; H, 4.1; N, 3.6; O, 24.6 %. The CCDC number for **1-PTB** is 2367585.

Synthesis of 1-TTB

The synthesis process of **1-TTB** is the same as that of **1-BTB**, except that H_3BTB is replaced with 4,4′,4″-triazine-1,3,5-triyl-tris(benzoic acid) (H_3TTB). Yield: 11.8 mg (72.8%). Elemental analysis data calculated (calcd.) for $C_{96}H_{48}N_{12}O_{25}Zn_6$: C, 53.3; H, 2.2; N, 7.8; O, 18.5 %. Found: C, 52.0; H, 3.7; N, 8.8; O, 24.6 %. The CCDC number for **1-TTB** is 2367589.

Synthesis of other catalysts

Zn cluster monomers [S1], r1^{Co} [S2], and MOF-5 [S3] were synthesized according to the reported references.

Synthesis of sulfenamides

The synthesis of sulfenamides refers to reported works.[S4-S7]

General procedure for the cycloaddition of CO_2 and epoxides

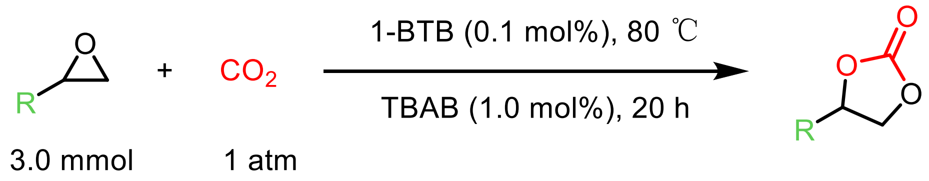

In a microwave reaction tube (10 mL) equipped with a stirring bar, epoxide (3.0 mmol), activated **1-BTB** (3.2 mg, 0.1 mol% based on trinuclear Zn unit) and tetrabutylammonium bromide (TBAB, 9.7 mg, 1.0 mol%) were added. The tube was evacuated and flushed with CO_2 from a CO_2 balloon for three times. The reaction mixture was stirred and heated at 80 °C for 20 h. After completion, the catalysts were collected by a filter and the resultant reaction mixture was washed with ethyl acetate (3 × 15 mL). The filtrate was concentrated in vacuo, and the catalytic conversion, selectivity, and yield were determined by ^1H NMR spectra using CH_2Br_2 as the internal standard.

Gram scale procedure

In a microwave reaction tube (20 mL) equipped with a stirring bar, styrene oxide (10.0 mmol), **1-BTB** (10.8 mg, 0.1 mol% based on trinuclear Zn unit) and TBAB (32.4 mg, 1.0 mol%) were added. The tube was evacuated and flushed with CO_2 from a CO_2 balloon three times. The reaction mixture was stirred and heated at 80 °C for 24 h. After completion, the catalyst was collected by filter and the resultant reaction mixture was washed with ethyl acetate (3 × 15 mL). The filtrate was concentrated in vacuo, and the catalytic conversion, selectivity, and yield were determined by ^1H NMR spectra using CH_2Br_2 as the internal standard.

Cycle procedure

In a dry flask (50 mL) equipped with a stirring bar, epoxide (20.0 mmol), activated **1-BTB** (22.0 mg, 0.1 mol% based on trinuclear Zn unit) and TBAB (65.0 mg, 1.0 mol%) were added. The tube was evacuated and flushed with CO_2 from a CO_2 balloon for three times. The reaction mixture was stirred and heated at 80 °C for 24 h. After completion, the catalyst was collected and washed by a filter and the crystals were activated with acetone for three times. The catalytic conversion, selectivity, and yield were determined by ^1H NMR spectra using CH_2Br_2 as the internal standard.

General procedure for the photocatalyzed monooxygenation

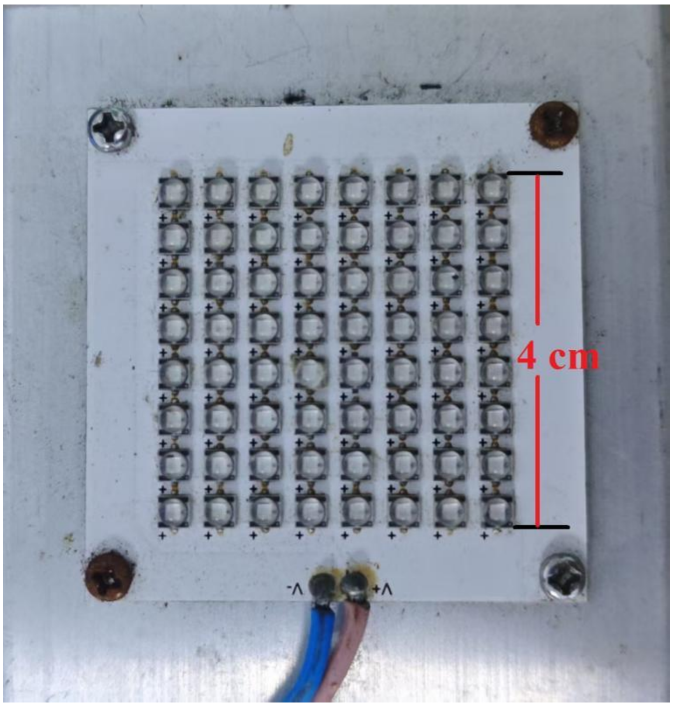

Figure S1 The light source used in this study**.**

The power of light sources used in this study is 60 W.

General procedure for the monooxygenation of sulfenamides

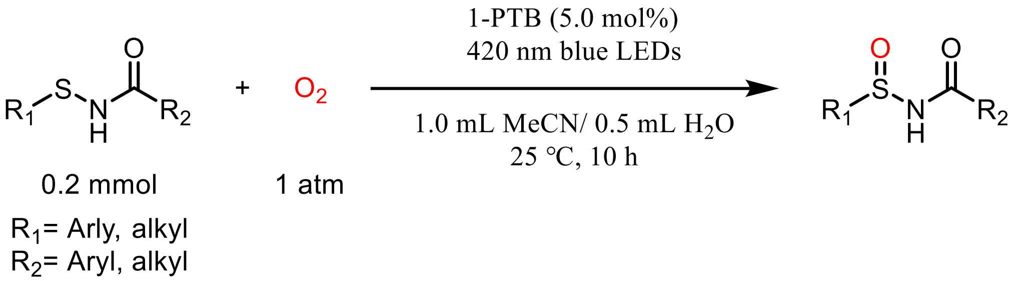

In a microwave reaction tube (10 mL) equipped with a stirring bar, sulfenamide (0.2 mmol) and activated **1-PTB** (20 mg, 5.0 mol% based on trinuclear Zn unit) were added. The tube was evacuated and flushed with O_2 from an O_2 balloon for three times. MeCN (1.0 mL) and H_2O (0.5 mL) were injected respectively into the tube *via* syringes. The reaction mixture was stirred and lit at 420 nm blue LED light at 25 °C for 10 h. After completion, the catalyst was collected by filter and the resultant reaction mixture was washed with ethyl acetate (3 × 15 mL). The filtrate was concentrated in vacuo, and the crude residue was subjected to flash column chromatography on silica gel to yield the desired product.

Gram scale procedure

In a microwave reaction tube (50 mL) equipped with a stirring bar, *N*-(*p*-tolylthio)benzamide (4.5 mmol, 1.1 g) and **1-PTB** (450 mg, 5.0 mol% based on trinuclear Zn unit) were added. The tube was evacuated and flushed with O_2 from an O_2 balloon three times. MeCN (22.5 mL) and H_2O (11.5 mL)

were injected respectively into the tube *via* syringes. The reaction mixture was stirred and lit at 420 nm blue LED light at 25 °C for 16 h. After completion, the catalyst was collected by filter and the resultant reaction mixture was washed with ethyl acetate (3 × 25 mL). The filtrate was concentrated in vacuo, and the crude residue was subjected to flash column chromatography on silica gel to yield the desired product.

Cycle procedure

After standard procedures, the catalyst was washed and activated with acetone for three times before use.

General procedure for the monooxygenation of sulfides

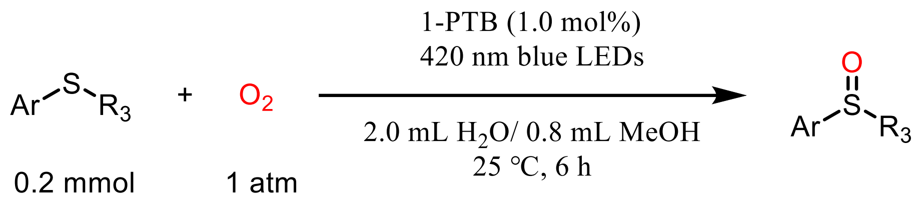

In a microwave reaction tube (10 mL) equipped with a stirring bar, sulfide (0.5 mmol) and activated **1-PTB** (10 mg, 1.0 mol% based on trinuclear Zn unit) were added. The tube was evacuated and flushed with O_2 from an O_2 balloon for three times. MeOH (0.8 mL) and H_2O (2.0 mL) were injected respectively into the tube *via* syringes. The reaction mixture was stirred and lit at 420 nm blue LED light at 25 °C for 6 h. After completion, the catalyst was collected by filter and the resultant reaction mixture was washed with ethyl acetate (3 × 15 mL). The filtrate was diluted with ethyl acetate (15 mL) and washed H_2O (3 × 10 mL), and brine (3 × 10 mL). The organic phase was dried over Na_2SO_4 and concentrated in vacuo, and the crude residue was subjected to flash column chromatography on silica gel to yield the desired product.

Photoelectrochemical measurements

The photoelectrochemical analysis was conducted on a BAS Epsilon electrochemical system with a conventional three-electrode cell, which used a Pt plate as the counter electrode and an Ag/AgCl electrode as the reference electrode. The working electrode was prepared on ITO glass that was cleaned by sonication in ethanol and then dried at 353 K for 1 h. Typically, 5 mg of the sample was fully dispersed in a mixture of 900 μL of *N*,*N*-Dimethylformamide (DMF) and 100 μL Nafion membrane-solution by sonication to get suspension. The suspension (20 μL) was spread onto the exposed area (0.25 cm^2) of the ITO glass, whose boundary was protected using scotch tape. After air drying, the working electrode is placed in the air overnight and is then ready for testing. The

photocurrent density was in 0.2 M Na_2SO_3 aqueous solution. The electrochemical impedance spectroscopy (EIS) was conducted in 0.5 M KCl aqueous solution containing $K_3[Fe(CN)_6]/K_4[Fe(CN)_6]$ (0.01 M).

Density functional theory (DFT) calculations [S8-S16]

Metal units and ligand molecular fragments for structure optimization, energy, and electronic properties were calculated using Gaussian 16 B.01 software at B3LYP/6-311 g(d,p), the D3 version of Grimme's dispersion was employed and vibration frequency was calculated at the same theoretical level to ensure that the structure is minimum (no imaginary frequency). The dipole moment was calculated by B3LYP-D3/def2-TZVPD with higher precision. The ESP map and molecular orbitals were generated using Gaussview 6.0 and the ESP was evaluated by Multiwfn based on the highly effective algorithm proposed.

SI-2 Crystal structure description and characterization

Crystal data and structure refinement details

Table S1 Crystal data and structure refinement details of **1-BTB**, **1-PTB**, and **1-TTB.**

**Complex**	**1-BTB**	**1-PTB**	**1-TTB**
Formula	$C_{108}H_{48}O_{25}Zn_6$	$C_{104}H_{56}N_4O_{25}Zn_6$	$C_{96}H_{48}N_{12}O_{25}Zn_6$
formula weight, fw	2137.68	2153.74	2161.68
Temperature, *T* [K]	273.15	296.15(10)	296.15(10)
crystal system	*cubic*	*cubic*	*cubic*
space group	*Fd-3*	*Fd-3*	*Fd-3*
a [Å]	26.042(3)	25.8569(3)	25.6877(2)
b [Å]	26.042(3)	25.8569(3)	25.6877(2)
c [Å]	26.042(3)	25.8569(3)	25.6877(2)
α [°]	90	90	90
β [°]	90	90	90
γ [°]	90	90	90
V [$Å^3$]	17661(6)	17287.4(6)	16950.2(4)
Z	8	8	8
ρ [g cm^{-3}]	1.608	1.655	1.694
μ [mm^{-1}]	1.687	1.725	1.762
θ range	2.706-23.65	1.9190-19.3560	1.9320-23.9410
F(000)	8608	8704	8704
goodness-of-fit, GOF	1.146	1.070	1.120
R_1^a [I > 2σ (I)]	0.0772	0.0752	0.0651
wR_2^b (all data)	0.2425	0.2447	0.2005

$^aR_1 = ||F_o| - |F_c|| / |F_o|$. $^bwR_2 = [w(|F_o^2| - |F_c^2|)^2 / w|F_o^2|^2]^{1/2}$.

Crystal data of MOFs were tested on a Bruker D8 Venture using Mo Kα radiation. The empirical absorption correction was performed using the Crystal Clear program. The structure was solved by direct methods and refined on F^2 by the full-matrix least-squares technique using the SHELXL program package. Table S1 shows the refinement details of **1-BTB**, **1-PTB**, and **1-TTB.**

Table S2 ICP-OES data of **1-BTB**, **1-PTB**, and **1-TTB**.

	**1-BTB**	**1-PTB**	**1-TTB**
The content of Zn	15.3 wt%	14.3 wt%	13.6 wt%

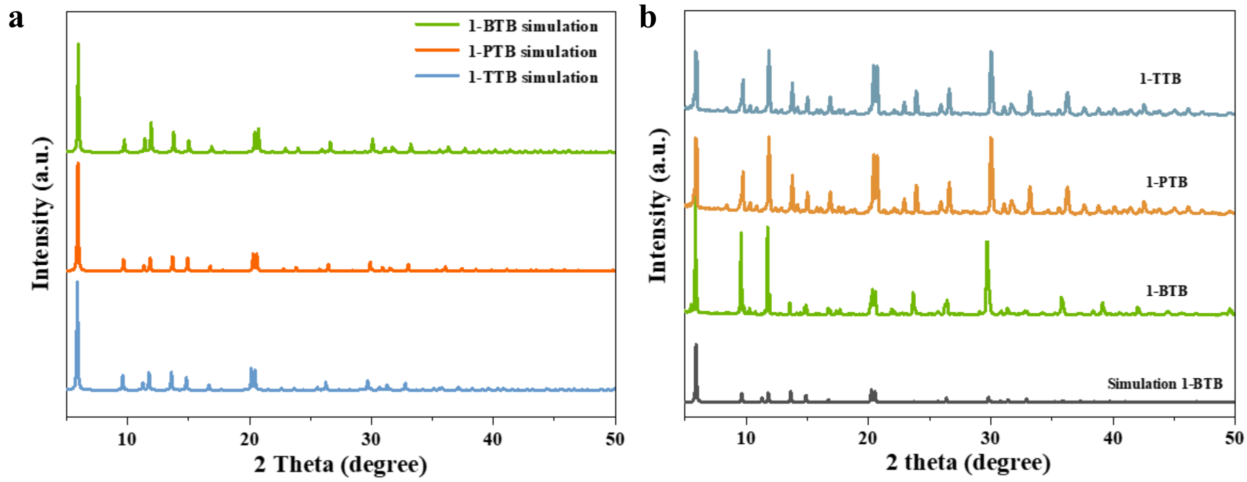

Figure S2 Simulated PXRD patterns (a) and PXRD patterns (b) of **1-BTB**, **1-PTB**, and **1-TTB.**

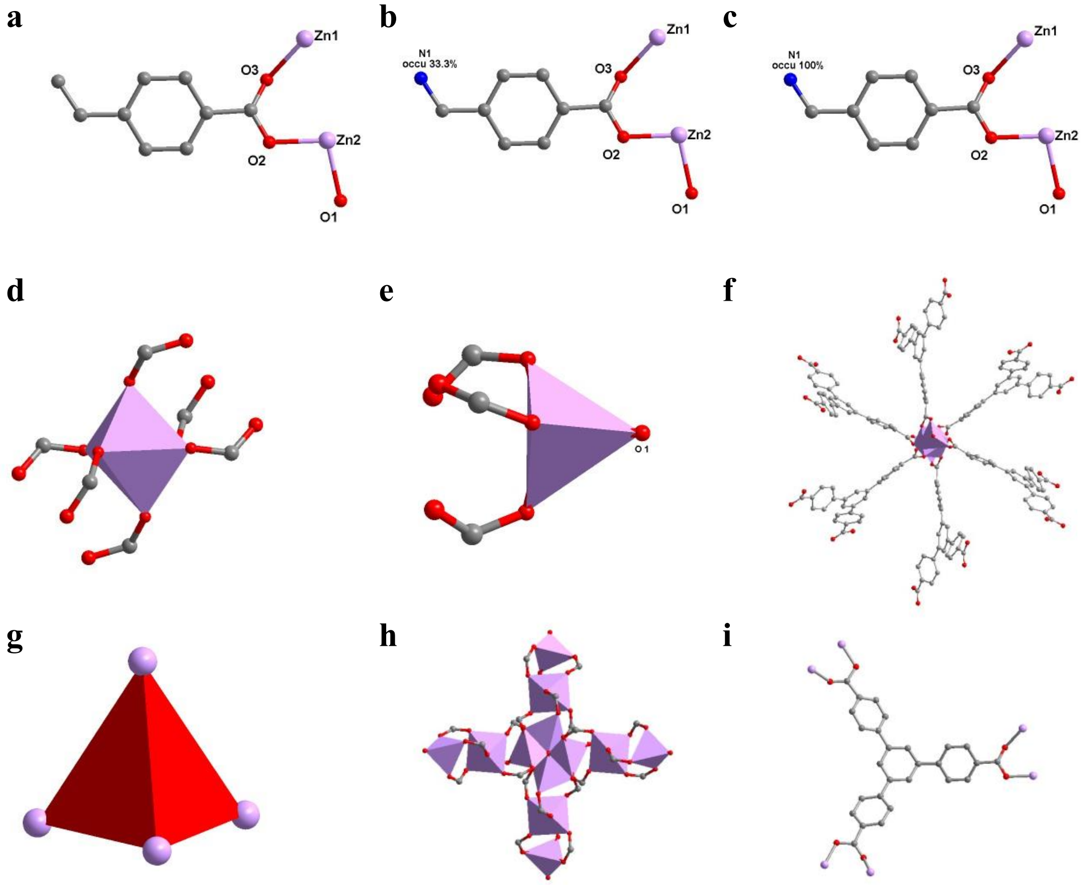

Figure S3 The asymmetric units of **1-BTB** (a), **1-PTB** (b), and **1-TTB** (c), respectively. It is worth noting that the atomic occupancy of N in **1-PTB** is 33.3%. Coordination environments of Zn1 (d), Zn2 (e), trinuclear Zn cluster (f), μ_4-O (g, h), and the ligand (i).

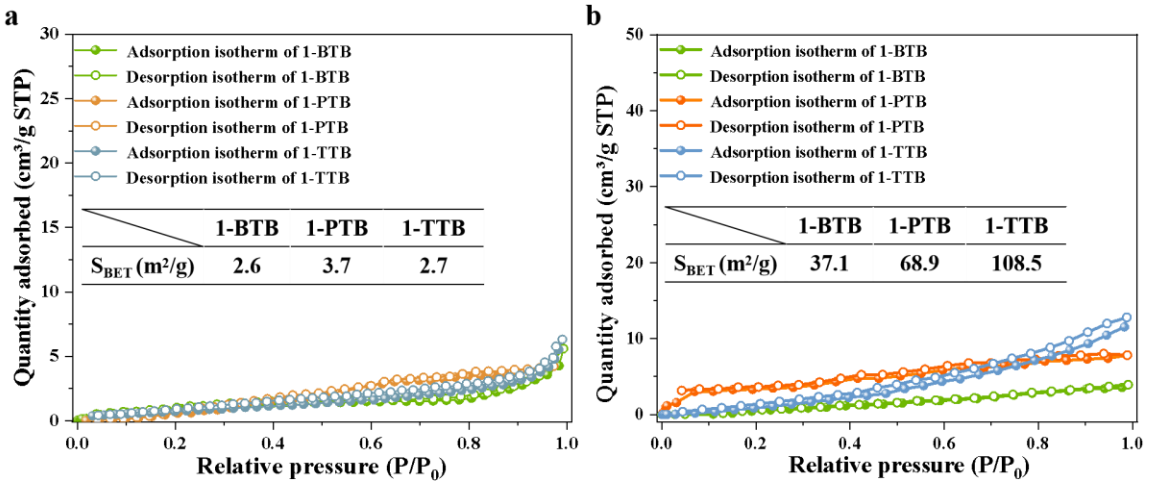

Figure S4 (a) The N_2 adsorption-desorption isotherms (P_0=760 mmHg). (b) The CO_2 adsorption-desorption isotherms (P_0=12000 mmHg). Inner tables: surface area analyzed by the density functional theory (DFT) method.

There is not much difference in the surface areas measured under N_2 conditions, which are 2.6 m^2/g, 3.7 m^2/g, and 2.7 m^2/g for **1-BTB**, **1-PTB**, and **1-TTB**, respectively (Figure S4a). Figure S4b shows that the BET surface area of MOFs increases with the increase of N element content, which may be due to the adsorption of acidic CO_2 by basic N atom sites, thereby resulting in the enrichment of CO_2 on the surface of MOFs.

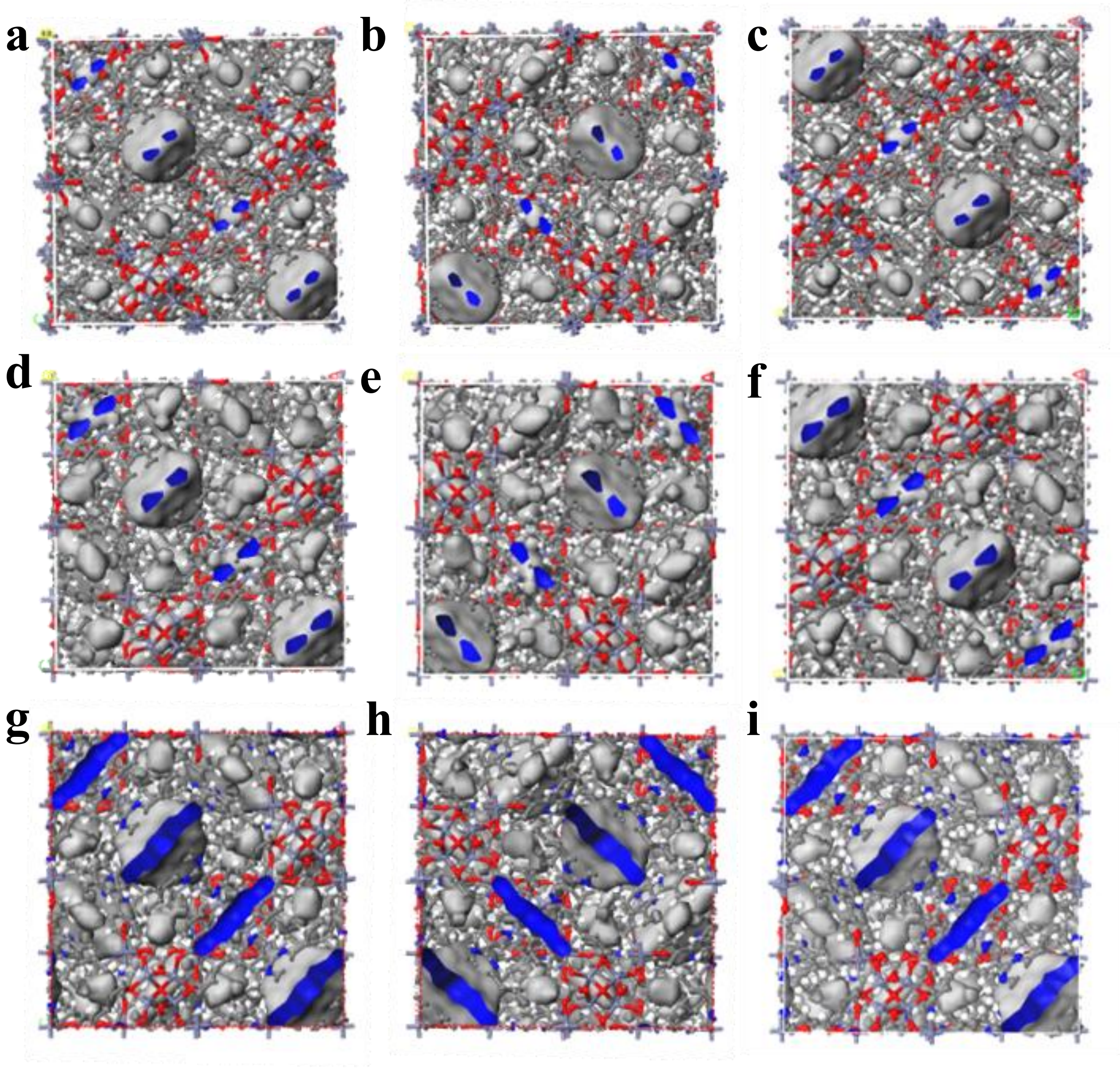

Figure S5 Connolly surface of **1-BTB** (a, b, c), **1-PTB** (d, e, f), and **1-TTB** (g, h, i) on the *bc* plane (a, d, g), *ac* plane (b, e, h) and *bc* plane (c, f, i).

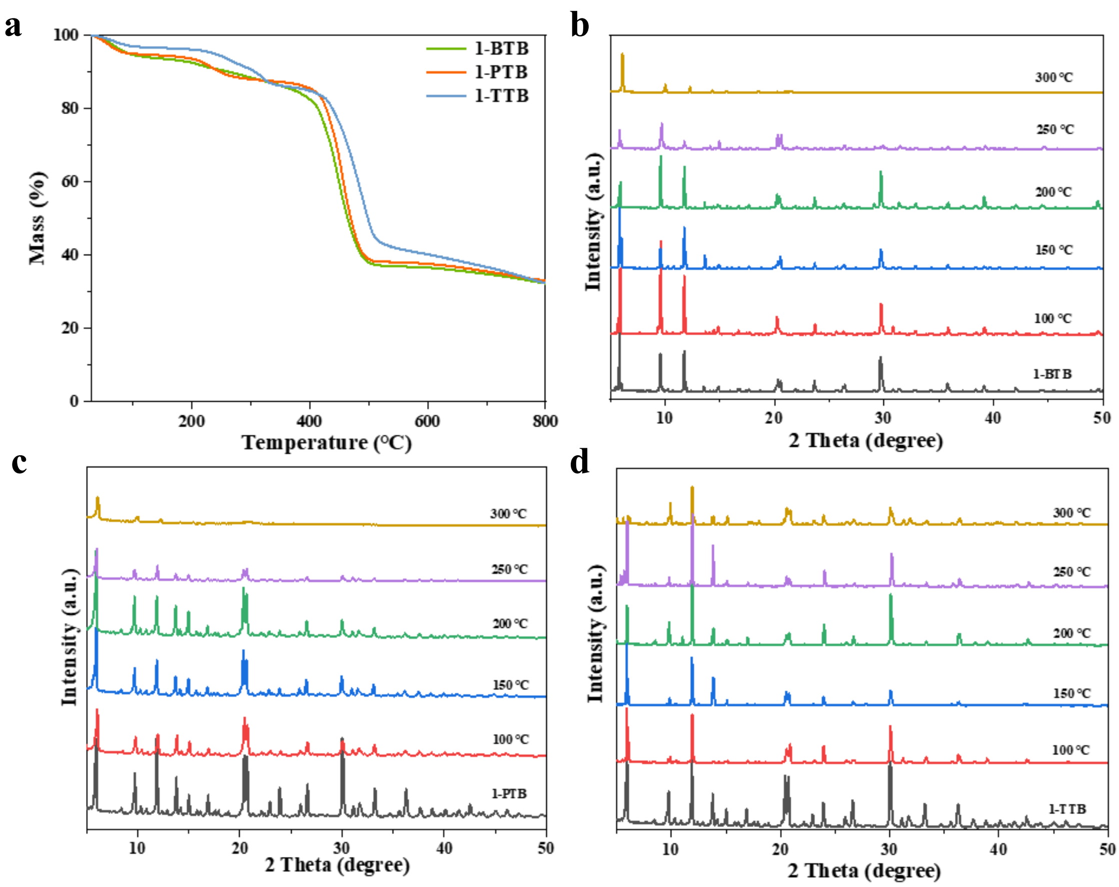

Figure S6 (a) The TGA of **1-BTB**, **1-PTB**, and **1-TTB** under N_2 atmosphere. The PXRD patterns of **1-BTB** (b), **1-PTB** (c), and **1-TTB** (d) at various temperatures for 1 h.

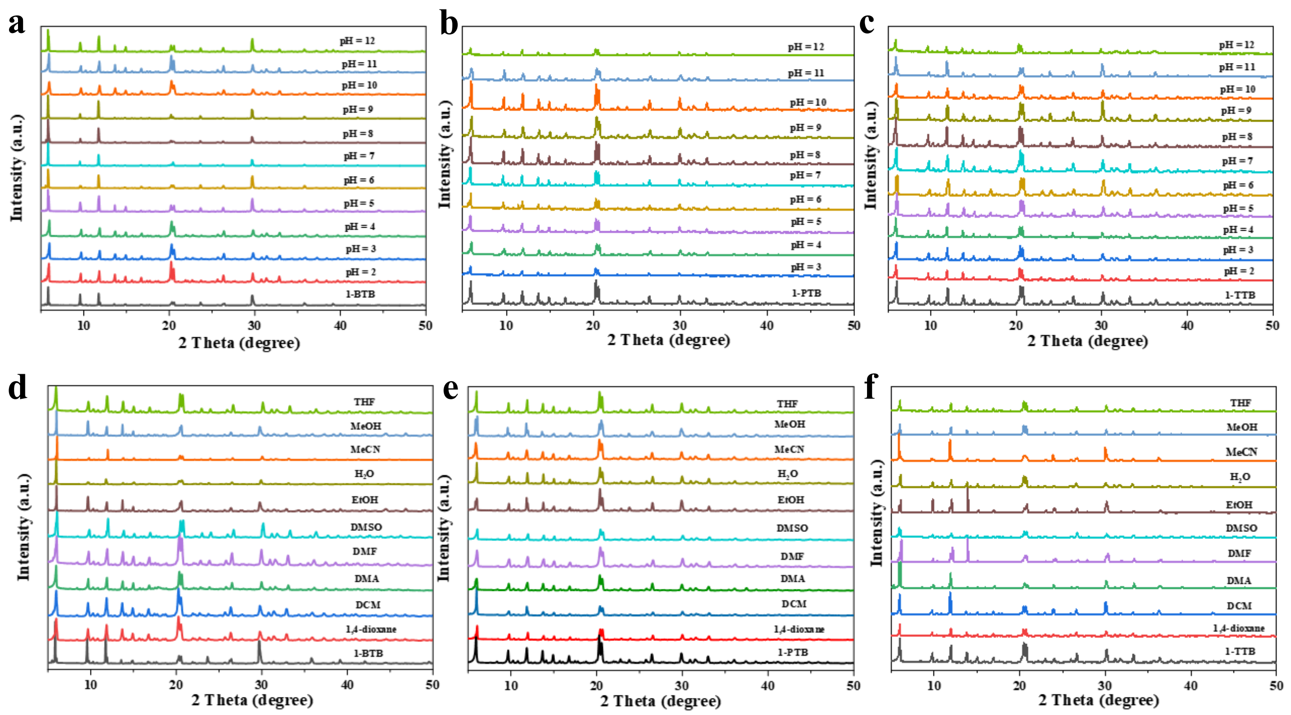

Figure S7 Chemical stability tests of **1-BTB** (a), **1-PTB** (b), and **1-TTB** (c) soaked in different pH aqueous solutions for 24 h. Solvent stability tests of **1-BTB** (d), **1-PTB** (e), and **1-TTB** (f) soaked in different solvents for 30 days.

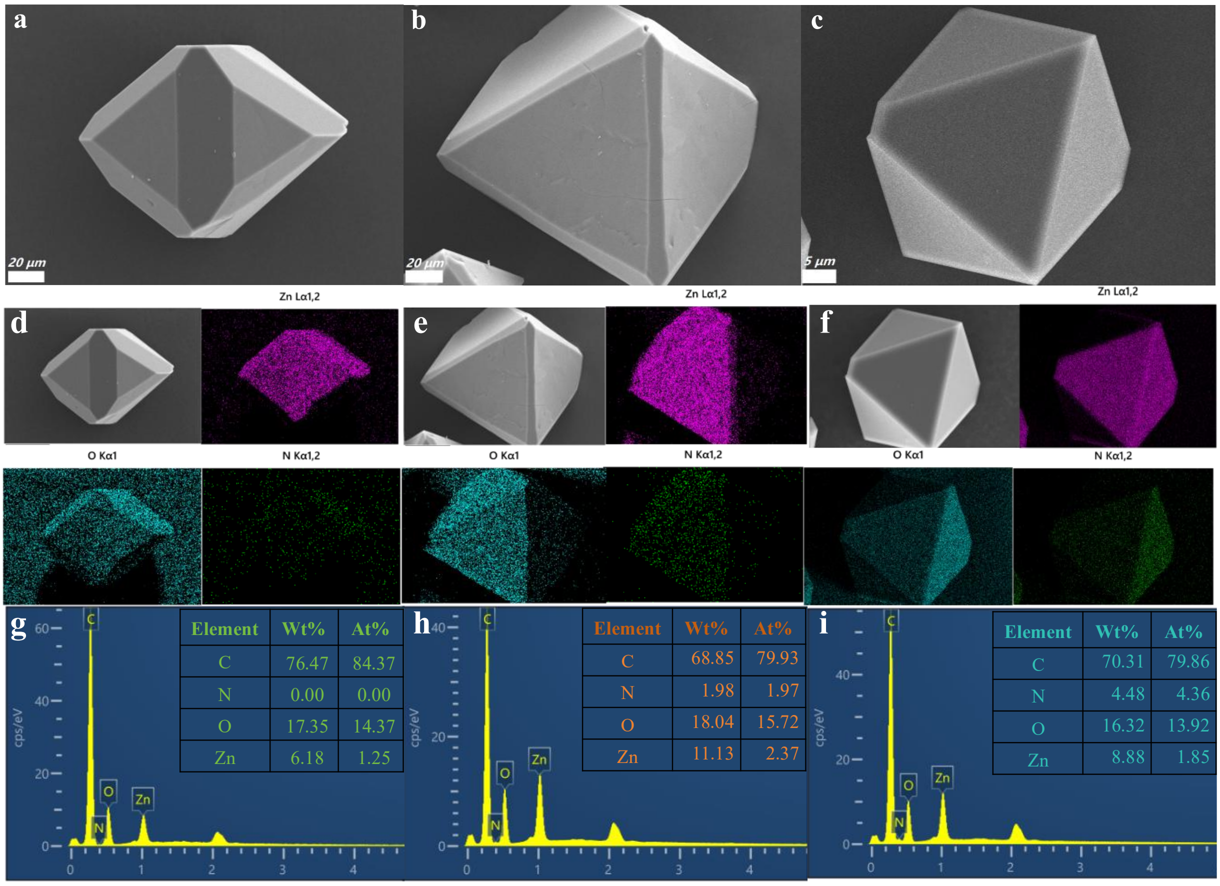

Figure S8 The SEM images of **1-BTB** (a), **1-PTB** (b), and **1-TTB** (c). EDS-mapping of **1-BTB** (d), **1-PTB** (e), and **1-TTB** (f). EDS-spectra of **1-BTB** (g), **1-PTB** (h), and **1-TTB** (i). Inner tables: The content of different elements analysed by EDS.

The increase of the content of N element with increasing of N atoms in the ligand is consistent with the results of elemental analysis (Figure S8g-i).

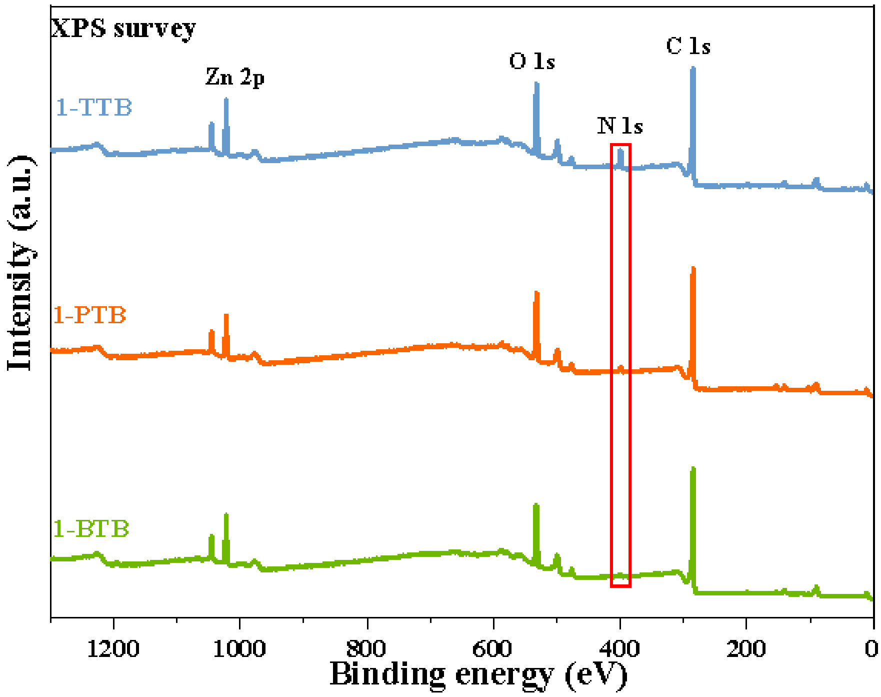

Figure S9 (a) The XPS survey spectra of **1-BTB**, **1-PTB**, and **1-TTB**.

XPS survey spectra show that the characteristic peak of the N element gradually increases with the increase of the content of N atoms in ligands (Figure S9).

SI-3 Lewis acidity study of MOFs in the CO_2 cycloaddition

The Lewis acidity of the catalysts was determined by TPD-NH_3. Before NH_3 adsorption, the sample was activated at 120 °C for 8 h. After adsorption of NH_3 at 50 °C for 0.5 h, the desorption step was performed from 50 to 400 °C at a heating rate of 10 °C/min. The desorption peak in the range of 50-200 °C might be ascribed to the adsorption of NH_3 on the Lewis acid sites of catalysts.[S17]

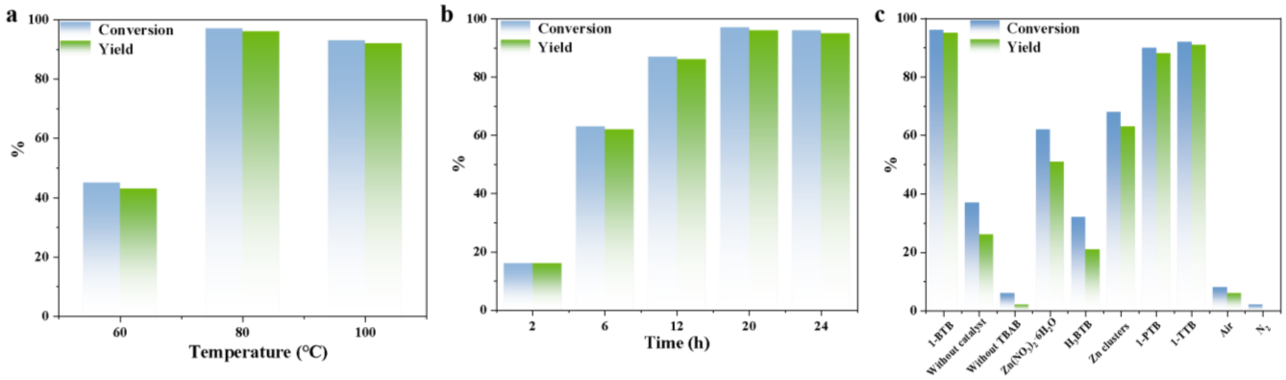

Figure S10 (a) Reaction temperature optimization. (b) Reaction time optimization. (c) Cycloaddition of CO_2 and styrene oxide under various conditions. The amount of catalyst is 0.1 mol% except for $Zn(NO_3)_2$•$6H_2O$ and H_3BTB (0.5 mol%). Standard conditions: catalyst (0.5 mol%), styrene epoxide (360.5 mg, 3.0 mmol), TBAB (9.7 mg, 1.0 mol%), solvent-free, CO_2 balloon at 80 °C for 20 h. Yield was analyzed by ^1H NMR with CH_2Br_2 as the internal standard.

As shown in Figures S10a and b, with the styrene epoxide as a model substrate, and **1-BTB** as a catalyst, the optimal conditions were obtained by controlling the temperature and time. The catalytic efficiency of **1-BTB** (3.2 mg, 0.1 mol% based on the trinuclear Zn units) could reach 96% in the presence of TBAB (9.7 mg, 1.0 mol%) as the co-catalyst at 80 °C for 20 hours (Figure S10c). In contrast, the yields were greatly reduced in the absence of the catalyst or co-catalyst (Figure S10c). In addition, the catalytic activity of $Zn(NO_3)_2 \cdot 6H_2O$ and H_3BTB ligands is much lower than **1-BTB** (Figure S10c). Zn cluster monomers with similar structures to $[Zn_3(\mu_4\text{-}O)_2(COO)_6]^-$ were used as catalysts with 63% yield.[S1] These results showed that **1-BTB** can effectively catalyze the cycloaddition of CO_2 and styrene oxide. With **1-PTB** and **1-TTB** as the catalysts, the yields were 88% and 91%, respectively. These results were slightly lower than **1-BTB** (Figure S10c). The reaction hardly occurs in the air or N_2 (Figure S10c).

Table S3 Comparison of catalytic activity of various MOF catalysts.

Entry	Catalyst	Yield (%)	Temperature (°C)	CO_2 pressure	TON	TOF (h^{-1})	References
**1**	MOF-801(D)	87	80	1 bar	145	9.7	[S18]
**2**	CuBDC-CMC	92	80	1 atm	183	10.8	[S19]
**3**	$Cu_3(BTC)_2$@iPOF-TM-Br^-	85	60	5 bar	94	3.9	[S20]
**4**	NU-1000 (Zr)	98	80	1-3 bar	98	24.5	[S21]
**5**	Nd MOF	97	80	1 bar	97	8.1	[S22]
**6**	Sm MOF	96	80	1 bar	96	8.0	[S22]
**7**	NUC-101a	99	55	5 atm	495	99	[S23]
**8**	NUC-103a	97	65	0.1 MPa	1021	113.4	[S24]
**9**	MOF-74 (Zn, Mg)	62	80	8 bar	105	21.0	[S25]
**10**	Ce-NU-1008	95	Room temperature	1 bar	95	4.75	[S26]
**11**	$r1^{Co}$	98	80	1 atm	196	19.6	[S2]
**12**	**1-BTB**	96	80	1 atm	960	48	**This work**

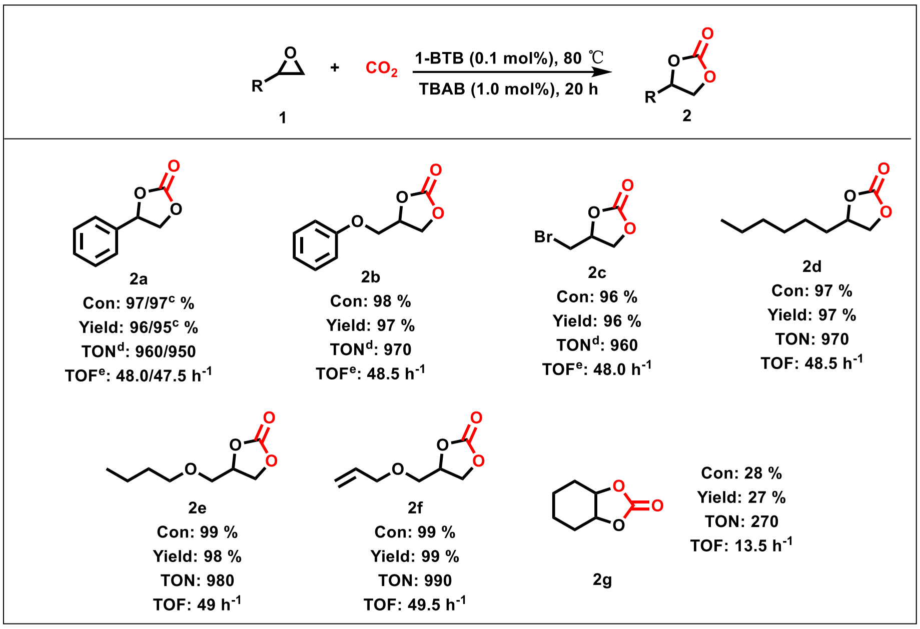

[a]Standard conditions: substrate (3.0 mmol), TBAB (9.7 mg, 1.0 mol%), **1-BTB** (3.2 mg, 0.1 mol% based on the trinuclear Zn units) , CO_2 balloon at 80 °C for 20 h; [b]Yield was analyzed by ^{1}H NMR, CH_2Br_2 as the internal standard; [c]10.0 mmol substrate; [d]TON: = (moles of product) / (moles of trinuclear Zn unit); [e]TOF: = TON / (reaction time).

Scheme S1 Substrate scope[a].

The substrate scope was further studied encouraged by the excellent catalytic performance of **1-BTB** (Scheme S1). Whether the terminal groups are aromatic or alkyl, the corresponding product could be obtained in excellent yields (**2a-f**). Moreover, common electron-withdrawing groups (**2b**, **2c**, **2e**, and **2f**) were converted in almost quantitative yields. However, due to the large steric hindrance of cyclohexene oxide (**2g**), a lower yield (27%) was achieved. After expanding the reaction scale, excellent yields were still maintained (10 mmol and 20 mmol styrene oxide for 97% and 90% yield, respectively), demonstrating the potential application value.

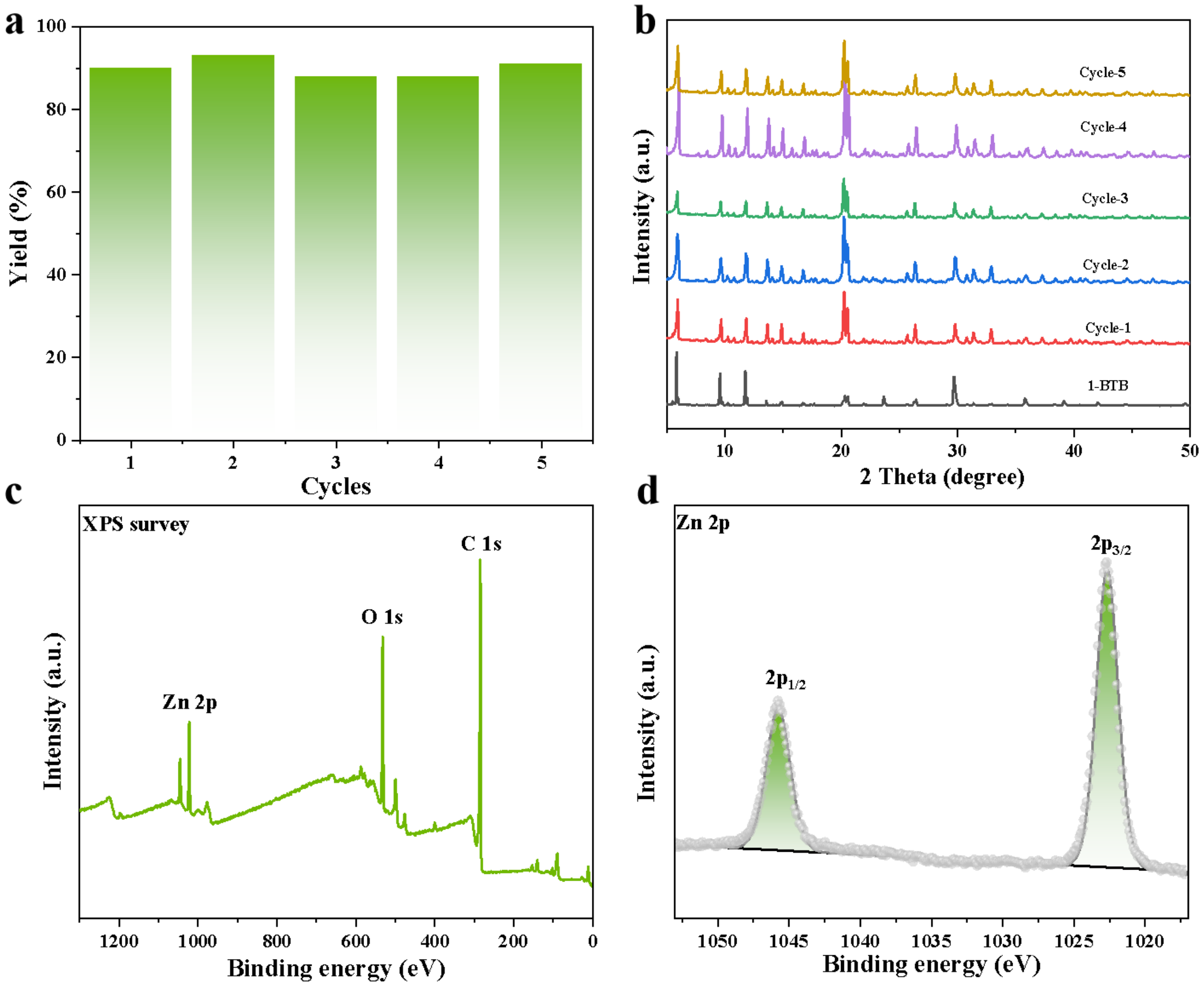

Figure S11 (a) Recycling performance of **1-BTB** with 20.0 mmol styrene oxide for 24 h in 5 cycles. (b) PXRD patterns after each cycle. The XPS survey spectra (c) and Zn 2p spectra (d) of **1-BTB** after cycles.

1-BTB could be recycled at least 5 times with good yields (Figure S11a). The PXRD patterns (Figure S11b) and XPS spectra (Figure S11c and d) after cycles highlighted the excellent recyclability and stability of **1-BTB**.

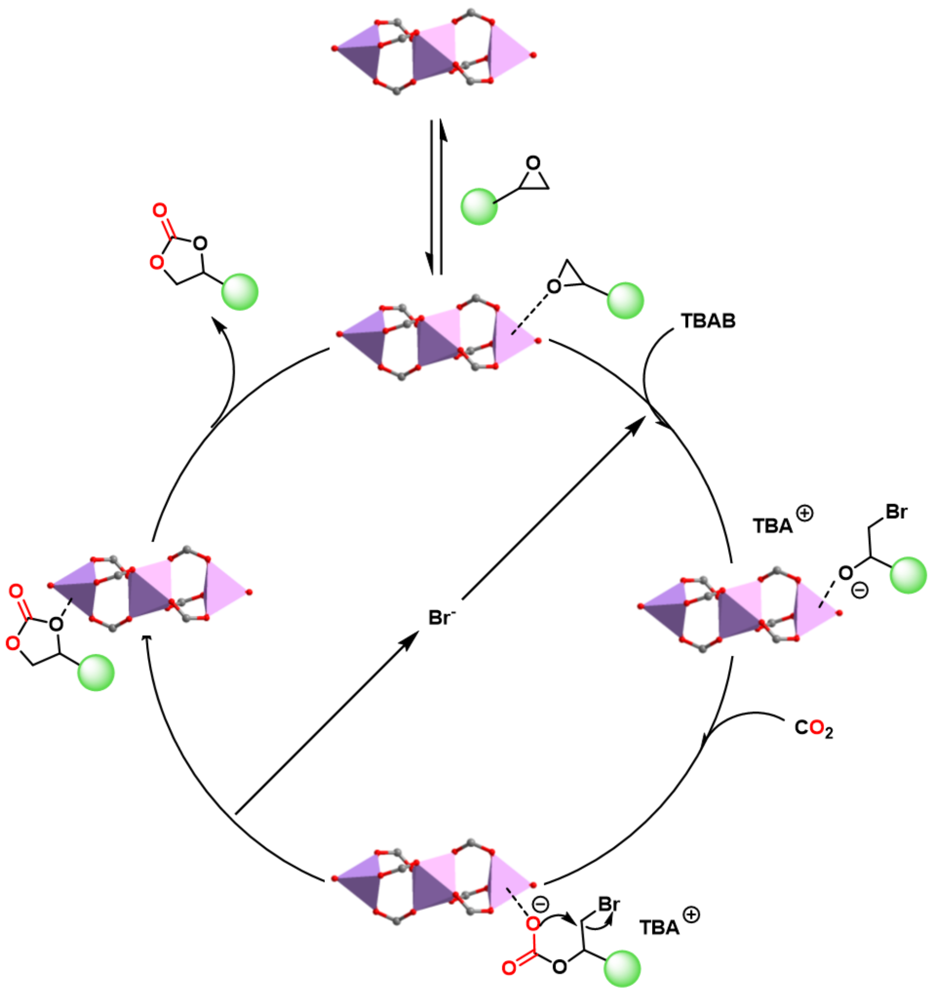

Figure S12 Proposed mechanism for the cycloaddition of CO_2 and epoxide.

The possible mechanism was proposed (Figure S12). Firstly, the epoxide is adsorbed and polarized by the trinuclear Zn unit. Secondly, the decisive ring-opening step is initiated when nucleophilic Br^- attacks the C atom with a small steric hindrance. Then, the ring-opened anionic attacks polarized CO_2 to form the carbonate species. Finally, the cyclic carbonate is obtained through intramolecular cyclization and desorption to regenerate the catalyst.[S27]

SI-4 Optical performance research

The Tauc plot is calculated using the Kubelka-Munk function.

$(\alpha h v)^2 = B(hv - E_g)$ Equation S1

Where h is Planck's constant, v is the frequency of light, B is a constant, E_g is the band gap, and α is the absorbance. Plot with $(\alpha hv)^2$ as the ordinate and hv as the abscissa. The x-axis intercept obtained by fitting the linear region is the band gap.

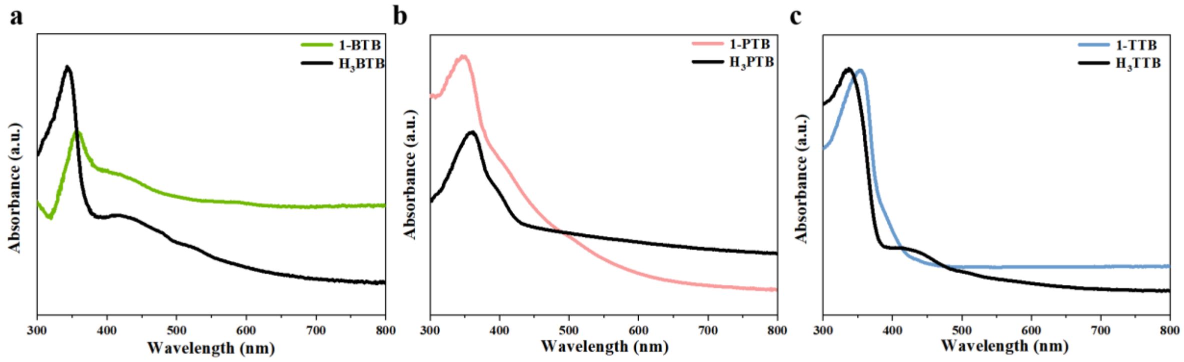

Figure S13 UV/vis DRS spectra of **1-BTB** and H_3BTB (a), **1-PTB** and H_3PTB (b), and **1-TTB** and H_3TTB (c).

The UV/vis DRS spectra of **1-BTB**, **1-PTB**, **1-TTB**, and the corresponding ligands were compared (Figure S13). The shift in peak position indicates that the electron transfer occurs in the frameworks.

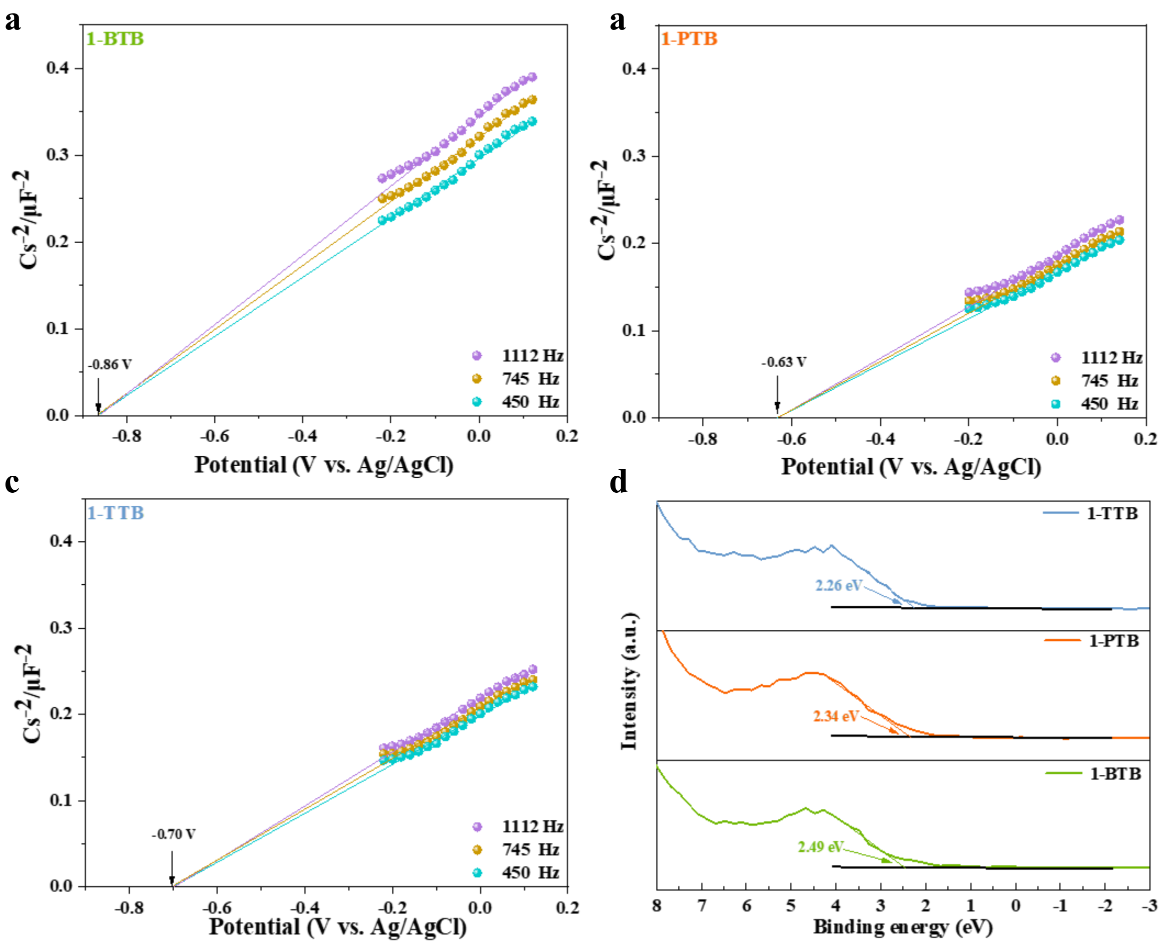

Figure S14 Mott-Schottky plots of **1-BTB** (a), **1-PTB** (b), and **1-TTB** (c). (d) The XPS valence band spectra of **1-BTB**, **1-PTB**, and **1-TTB**.

The E_{VB} value of the corresponding normal hydrogen electrode ($E_{VB,NHE}$) obtained from XPS was calculated using the equation $E_{VB,NHE} = \varphi + E_{VB,XPS} - 4.44$, where φ is the work function of the instrument (4.2 eV).[S28]

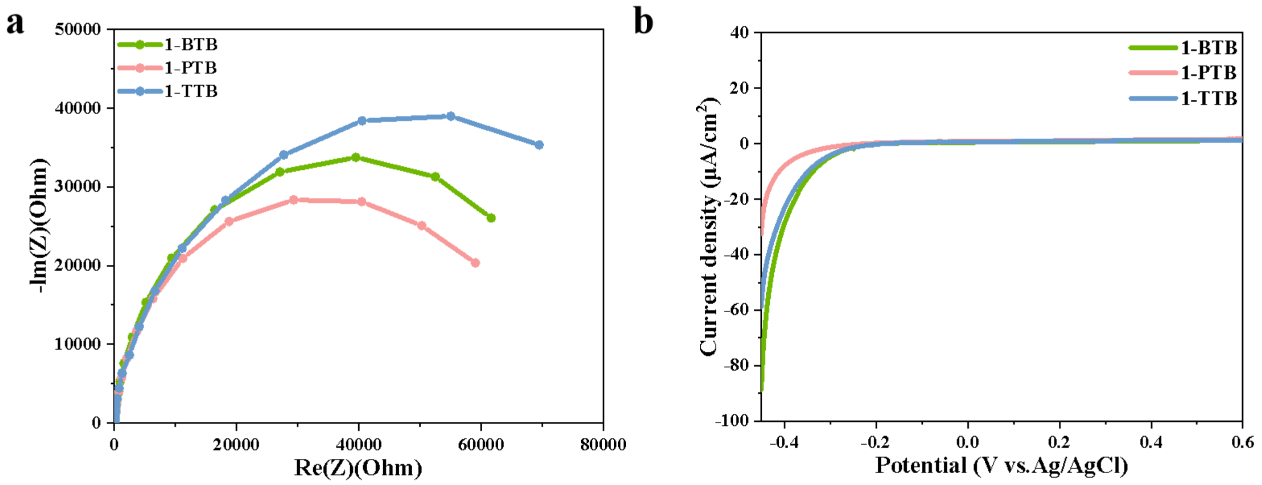

Figure S15 (a) Nyquist plots of **1-BTB**, **1-PTB**, and **1-TTB**. (b) LSV curves of **1-BTB**, **1-PTB**, and **1-TTB**.

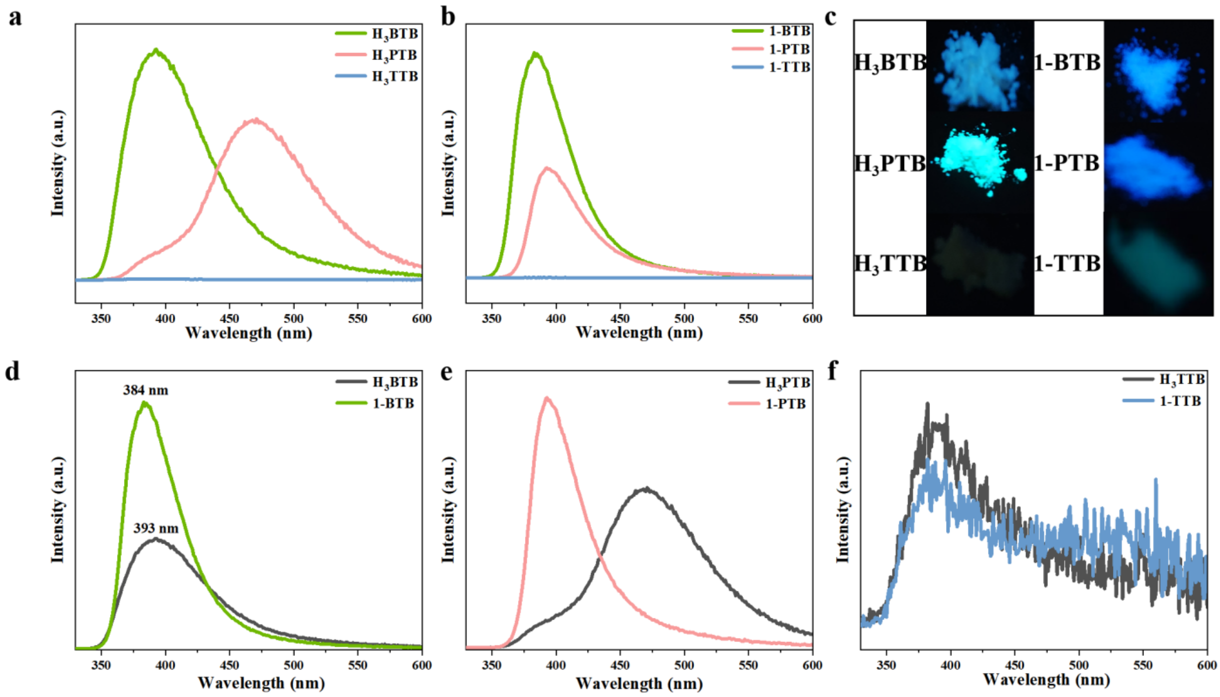

Figure S16 PL spectra of ligands (a) and MOFs (b). (c) PL digital images of ligands and MOFs at 365 nm. (d-f) Comparison of PL spectra of ligands and MOFs.

The H_3BTB and **1-BTB** both have the strongest PL ability, emitting blue light under excitation at 365 nm (Figure S16a-c). The PL intensity of H_3TTB and **1-TTB** is almost negligible (Figure S16a-c). The PL spectra of **1-PTB** are greatly blue-shifted compared with that of H_3PTB, resulting in the change of fluorescence from green to blue under excitation at 365 nm (Figure S16c-f). The huge fluorescence difference of **1-PTB** also indicates the MLCT mechanism.

.

SI-5 DFT calculations

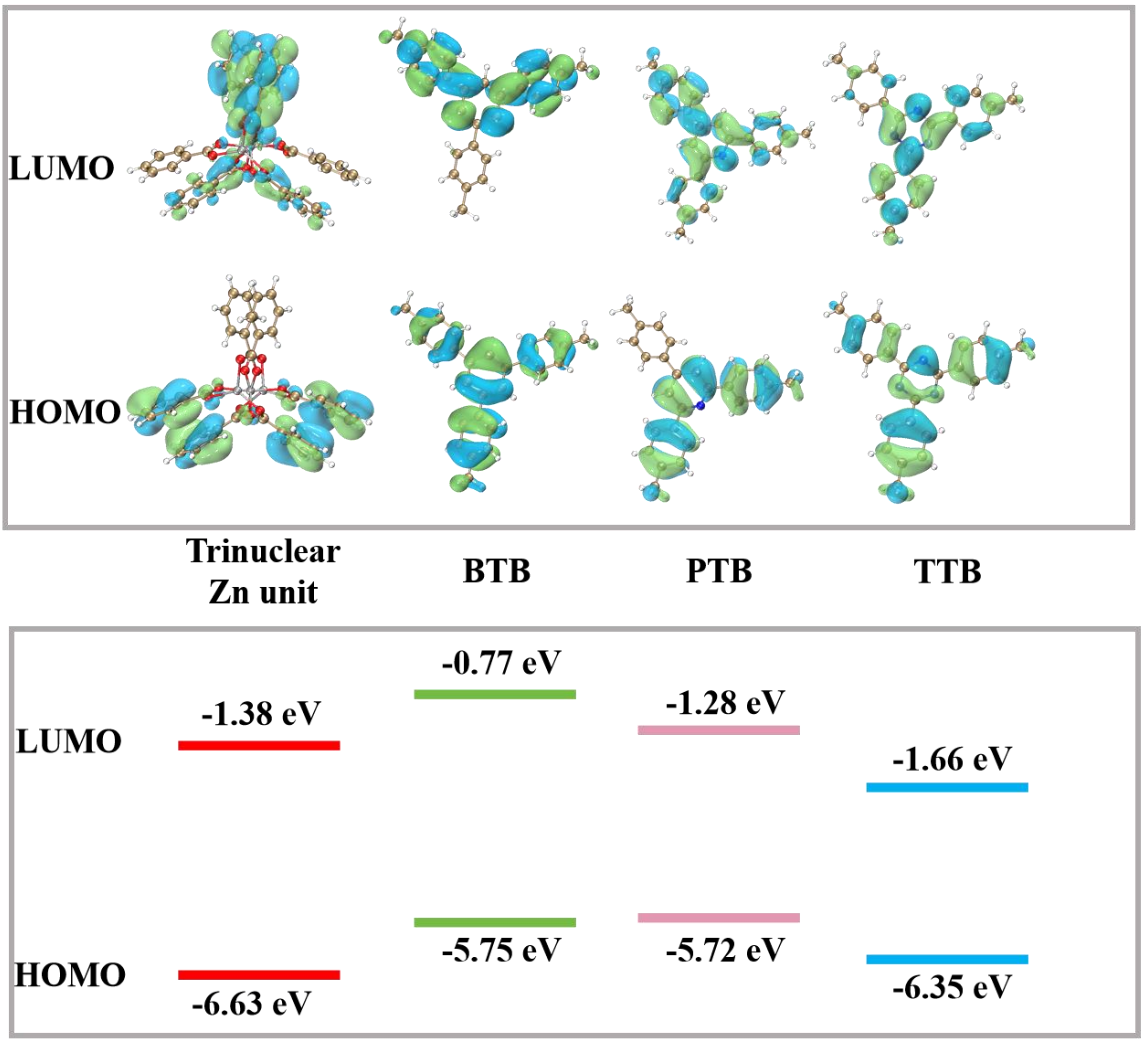

Figure S17 HOMO and LUMO energy level diagram for the trinuclear Zn unit model and molecular fragments models of ligands.

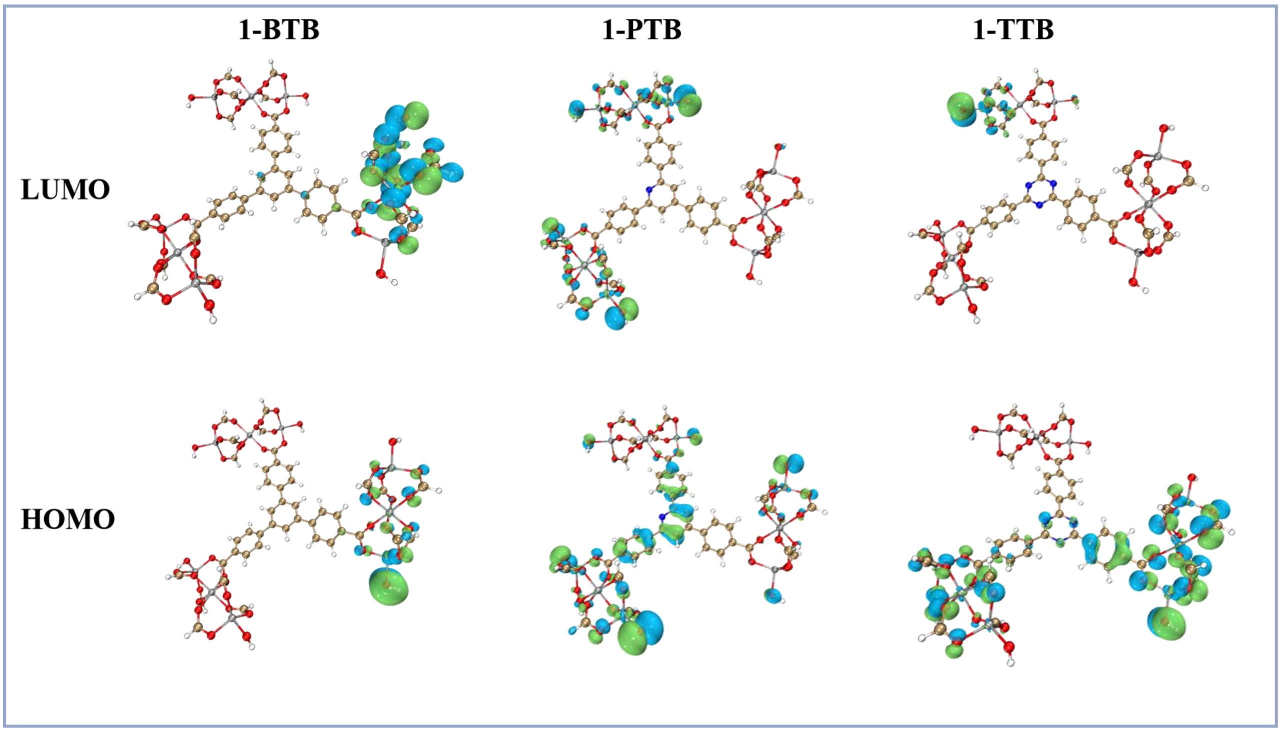

Figure S18 HOMO and LUMO energy level diagram for **1-BTB**, **1-PTB**, and **1-TTB**.

The primary contribution to the HOMO and LUMO orbitals in **1-BTB** and **1-TTB** almost originates from trinuclear Zn units. Figure S18 illustrates that for **1-PTB**, the HOMO is located at the ligands and trinuclear Zn units, whereas the LUMO is primarily located at the trinuclear Zn units, indicating the unusual charge transfer from trinuclear Zn units to ligands and the significant separation between the HOMO and LUMO orbitals. This enhanced separation aids in preventing carriers recombination and promoting photocatalytic efficiency (Figure S18).[S29]

SI-6 Photocatalytic monooxygenation of sulfenamides

Table S4 Crystal data and structure refinement details of **4i** and **4s.**

**Complex**	**4s**	**4i**
Formula	$C_{15}H_{15}NO_2S$	$C_{11}H_8NO_2S_2$
formula weight, fw	273.34	285.75
Temperature, T [K]	296	296
crystal system	*orthorhombic*	*monoclinic*
space group	*P 21 21 21*	*C 1 c 1*
a [Å]	5.5753(6)	10.5271(17)
b [Å]	13.8463(15)	16.939(3)
c [Å]	17.577(2)	7.636(2)
α [°]	90	90
β [°]	90	114.846(4)
γ [°]	90	90
V [$Å^3$]	1356.9(3)	1235.6(5)
Z	4	2
ρ [g cm^{-3}]	1.338	1.536
μ [mm^{-1}]	0.235	0.634
θ range	2.75-24.99	2.405-24.994
F(000)	576	584
goodness-of-fit, GOF	1.043	1.010
R_1^a [I > 2σ (I)]	0.0360	0.0369
wR_2^b (all data)	0.0884	0.0848

$^aR_1 = \|\,|F_o| - |F_c|\,\| / |F_o|$. $^b wR_2 = [w(|F_o^2| - |F_c^2|)^2 / w|F_o^2|^2]^{1/2}$.

Crystal data were tested on a Bruker D8 Venture using Mo Kα radiation. The empirical absorption correction was performed using the Crystal Clear program. The structure was solved by direct methods and refined on F^2 by the full-matrix least-squares technique using the SHELXL program package. Table S3 shows the refinement details of **4i** and **4s.**

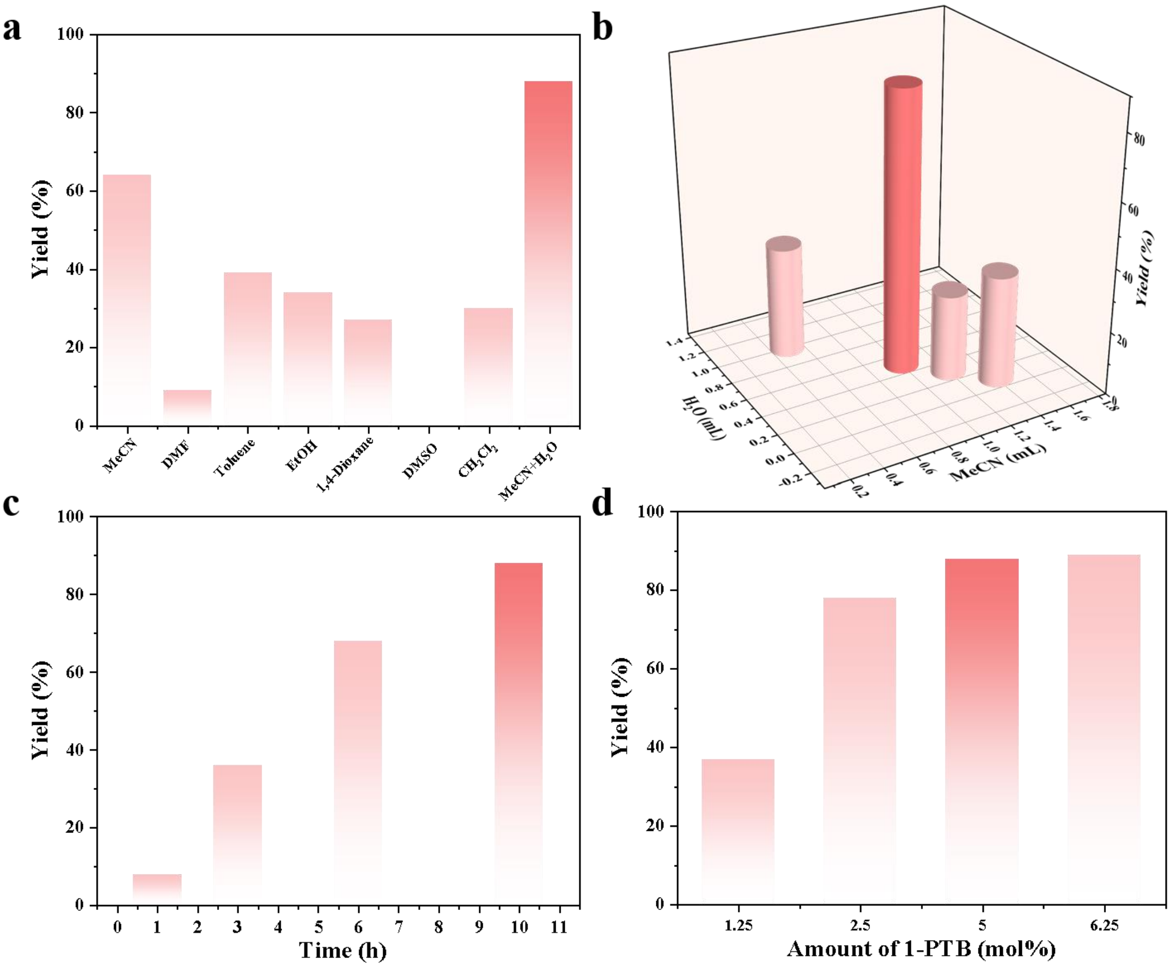

Figure S19 The condition optimization for photooxidation of sulfenamide in solvent type (a), solvent ratio (b), reaction time (c), and amount of **1-PTB** (d). Standard conditions: sulfenamide (51.9 mg, 0.2 mmol), **1-PTB** (20 mg, 5.0 mol% based on trinuclear Zn unit), MeCN (1.0 mL) and H_2O (0.5 mL) were reacted at 420 nm blue LED light for 10 h under O_2 ballon at 25 °C.

In the optimization of solvents, MeCN gave the highest yield (64%). When H_2O was used as an additive, the yield increased to 88%. Here, H_2O acts as the protic solvent to stabilize the intermediates in the reaction process (Figure S19a).[S30] Then, the optimal solvent ratio (1.5 mL, $v_{MeCN}/V_{H_2O} = 2$) was obtained (Figure S19b). The reaction time and catalyst amount were also evaluated (Figure S19c and d).

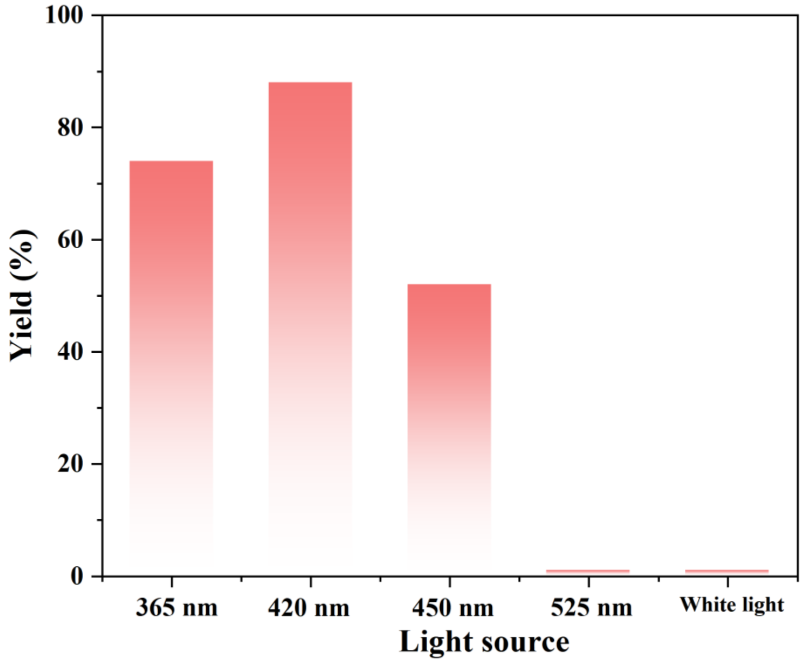

Figure S20 The effect of light wavelength on the photocatalytic monooxygenation of sulfenamides.

Figure S20 shows that the yield reaches the highest (88%) at the light source of 420 nm. As the light wavelength increases, the catalytic activity of **1-PTB** gradually decreases and even becomes inactivated, which is consistent with the results of UV/vis DRS spectra. The yield was 74% at a light source of 365 nm, but the skeleton of **1-PTB** after reaction had completely collapsed. In addition, white light with full wavelength failed to drive the reaction to occur.

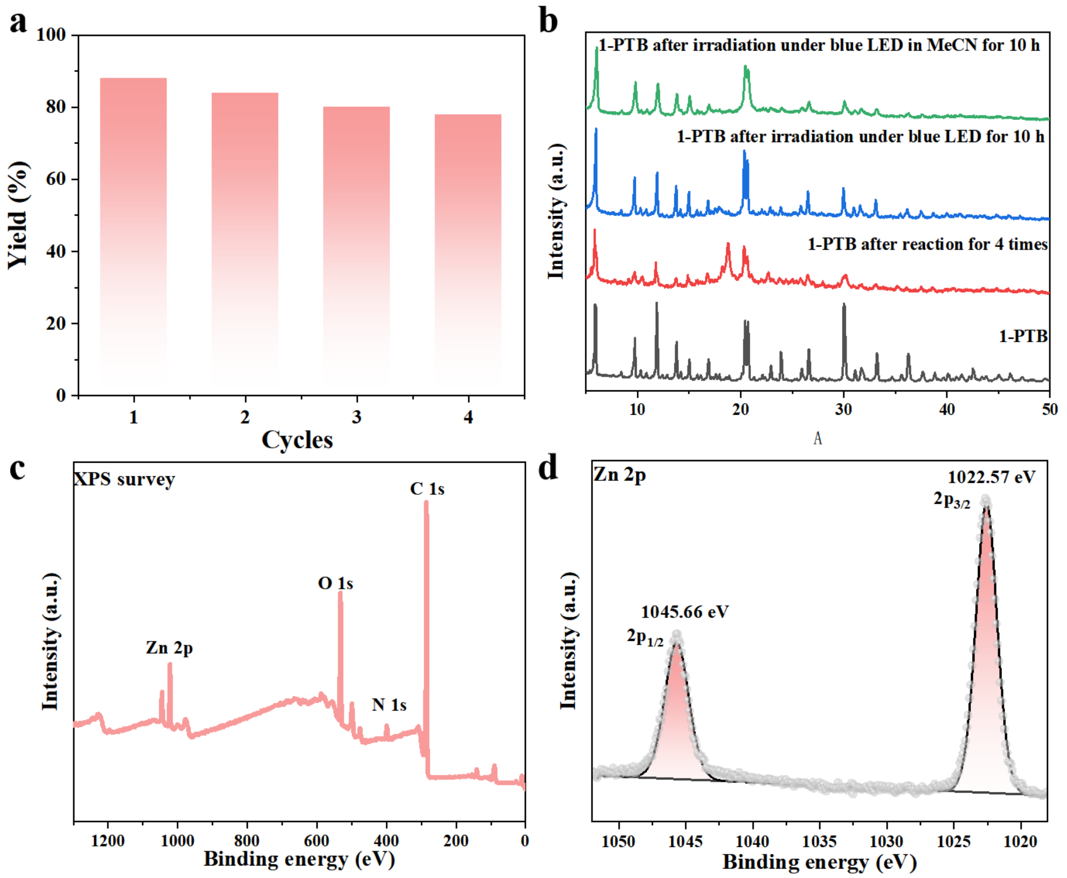

Figure S21 (a) Recycling experiments. Standard conditions: sulfenamide (0.2 mmol), **1-PTB** (5.0 mol%), MeCN (1.0 mL) and H_2O (0.5 mL), O_2 (1 atm), blue LEDs (420 nm), r.t., 10 h. (b) The PXRD patterns condition optimization. (c) The XPS survey spectrum of **1-PTB** after 4 cycles. (d) The Zn 2p XPS spectrum of **1-PTB** after 4 cycles.

The photostability of **1-PTB** was evaluated by exposing it to air and MeCN under a blue LED lamp for 10 h (Figure S21a). The crystallinity of **1-PTB** decreased after 4 cycles (Figure S21a). XPS spectra showed that the elemental composition of **1-PTB** and the coordination environment of Zn did not change significantly after 4 cycles (Figure S21b and c).

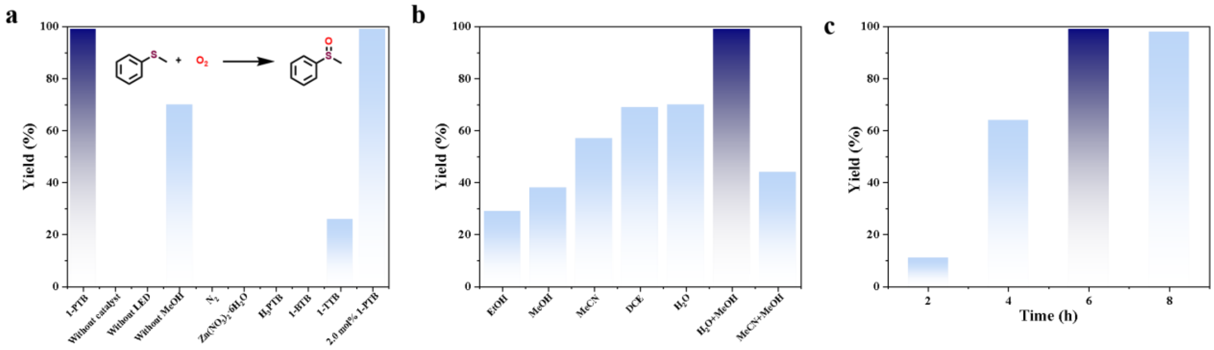

Figure S22 The optimization for photocatalytic monooxygenation of sulfide in various conditions (a), solvent type (b), and reaction time (c). Standard conditions: (Methylsulfinyl)benzene (62.1 mg, 0.5 mmol), **1-PTB** (10 mg, 1.0 mol% based on trinuclear Zn unit), H_2O (2.0 mL), and MeOH (0.8 mL) were reacted at 420 nm blue LED light for 6 h under O_2 ballon at 25 °C

We also optimized the reaction conditions of photocatalytic monooxygenation of sulfides (Figure S22). The (methylsulfinyl)benzene as a model substrate and **1-PTB** as the photocatalyst were reacted under a 420 nm LED lamp with an O_2 balloon for 6 hours at 25 °C to obtain the optimal yield (99%, Figure S22a). In the absence of photocatalysts, LED light, or O_2, the reaction cannot occur. $Zn(NO_3)_2 \cdot 6H_2O$, H_3PTB, or **1-BTB** have no catalytic activity. When the photocatalyst was replaced with **1-TTB**, the yield was reduced to 26%. It is particularly important to note that MeOH helps dissolve sulfide in the system (Figure S22a and b). The yield hardly changes when the amount of photocatalyst continues to increase (Figure S22c). The optimal time and solvent are 6 h and the mixed solution of MeOH and H_2O, respectively.

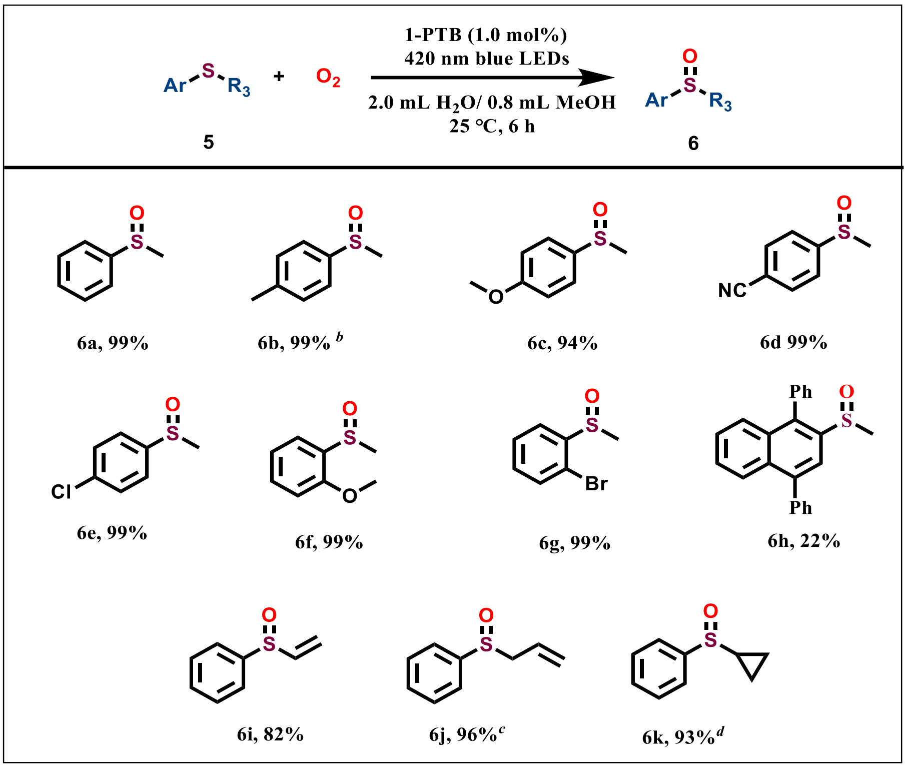

[a] Standard conditions: Sulfide (0.5 mmol), **1-PTB** (10 mg, 1.0 mol% based on trinuclear Zn unit), H_2O (2.0 mL) and MeOH (0.8 mL) were reacted at 420 nm blue LED light for 6 h under O_2 ballon at 25 °C; [b] Reaction time was 7 h; [c] Reaction time was 12 h; [d] Reaction time was 10 h.

Scheme S2 Substrate scope of sulfides[a].

The substrate scope of sulfides was also further investigated and evaluated (Scheme S2). Various electron-withdrawing or electron-donating para-substituents (**6a-e**) can be well tolerated. Different groups in the ortho position can also obtain the corresponding products in excellent yields (**6f** and **g**). The poor reaction effect for skeletons containing multiple benzene rings may be due to poor solubility (**6h**). Different groups for the alkyl moiety can also be well tolerated (**6i-k**).

SI-7 Study on the reaction mechanism for photocatalytic monooxygenation

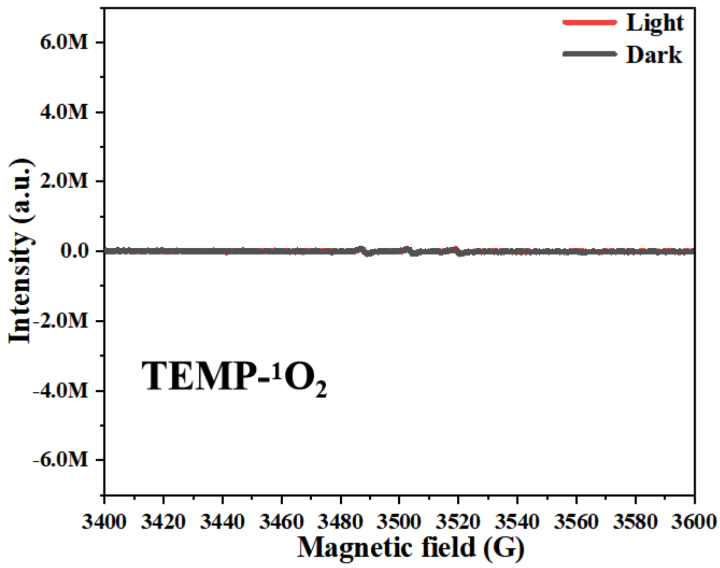

Figure S23 EPR spectra (in dark or under Xe lamp for 135 s) of **1-PTB** (1.0 mg/L) in air-saturated MeCN that contained 0.1 M TEMP

SI-8 NMR data

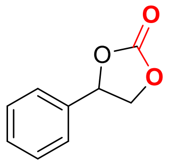

4-phenyl-1,3-dioxolan-2-one (**2a**)[S2]

The desired pure product was purified using silica gel chromatography (PE : EA = 10 : 1) to give **2a** as a white solid (472.9 mg, 96% yield).

^{1}H NMR (500 MHz, Chloroform-*d*) δ 7.45 - 7.38 (m, 3H), 7.36 - 7.32 (m, 2H), 5.66 (t, *J* = 8.0 Hz, 1H), 4.78 (t, *J* = 8.4 Hz, 1H), 4.31 (dd, *J* = 8.7, 7.8 Hz, 1H).

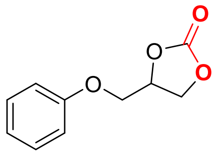

4-(phenoxymethyl)-1,3-dioxolan-2-one (**2b**)[S2]

The desired pure product was purified using silica gel chromatography (PE : EA=5 : 1) to give **2b** as a white solid (565.1 mg, 97% yield).

^{1}H NMR (400 MHz, Chloroform-*d*) δ 7.31 (t, *J* = 7.8 Hz, 2H), 7.02 (t, *J* = 7.4 Hz, 1H), 6.91 (d, *J* = 8.0 Hz, 2H), 5.08 - 4.98 (m, 1H), 4.62 (t, *J* = 8.4 Hz, 1H), 4.54 (dd, *J* = 8.5, 5.9 Hz, 1H), 4.24 (dd, *J* = 10.6, 4.2 Hz, 1H), 4.14 (dd, *J* = 10.6, 3.6 Hz, 1H).

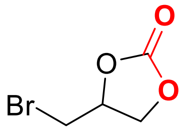

4-(bromomethyl)-1,3-dioxolan-2-one (**2c**)[S2]

The desired pure product was purified using silica gel chromatography (PE) to **2c** as a colorless oil (521.3 mg, 96% yield).

^{1}H NMR (500 MHz, Chloroform-*d*) δ 5.00 - 4.91 (m, 1H), 4.56 (t, *J* = 8.6 Hz, 1H), 4.30 (dd, *J* = 8.9, 5.9 Hz, 1H), 3.60 (dd, *J* = 11.4, 5.5 Hz, 1H), 3.54 (dd, *J* = 11.3, 3.8 Hz, 1H).

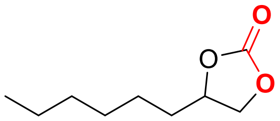

4-hexyl-1,3-dioxolan-2-one (**2d**)[S2]

The desired pure product was purified using silica gel chromatography (PE) to give **2d** as a colorless oil (501.1 mg, 97% yield).

^{1}H NMR (400 MHz, Chloroform-*d*) δ 4.68 - 4.60 (m, 1H), 4.46 (td, *J* = 8.1, 1.2 Hz, 1H), 3.99 (ddd, *J* = 8.4, 7.1, 1.4 Hz, 1H), 1.80 - 1.67 (m, 1H), 1.65 - 1.56 (m, 1H), 1.39 - 1.17 (m, 8H), 0.80 (td, *J* = 6.9, 1.5 Hz, 3H).

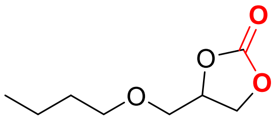

4-(butoxymethyl)-1,3-dioxolan-2-one (**2e**)[S2]

The desired pure product was purified using silica gel chromatography (PE) to give **2e** as a colorless oil (512.1 mg, 98% yield).

^{1}H NMR (500 MHz, Chloroform-*d*) δ 4.78 - 4.71 (m, 1H), 4.47 - 4.37 (m, 1H), 4.34 - 4.23 (m, 1H), 3.64 - 3.55 (m, 1H), 3.53 - 3.46 (m, 1H), 3.41 (qt, *J* = 6.6, 2.3 Hz, 2H), 1.50 - 1.39 (m, 2H), 1.32 - 1.20 (m, 2H), 0.85 - 0.76 (m, 3H).

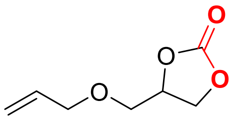

4-((allyloxy)methyl)-1,3-dioxolan-2-one (**2f**)[S2]

The desired pure product was purified using silica gel chromatography (PE) to give **2f** as a colorless oil (469.9 mg, 99% yield).

^{1}H NMR (500 MHz, Chloroform-*d*) δ 5.86 - 5.74 (m, 1H), 5.25 - 5.08 (m, 2H), 4.84 - 4.71 (m, 1H), 4.44 (td, *J* = 8.4, 3.4 Hz, 1H), 4.35 - 4.26 (m, 1H), 4.03 - 3.91(m, 2H), 3.67 - 3.58 (m, 1H), 3.57 - 3.46 (m, 1H).

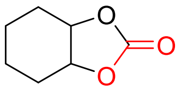

hexahydrobenzo[d][1,3]dioxol-2-one (**2g**)[S2]

The desired pure product was purified using silica gel chromatography (PE) to give **2g** as a colorless oil (115.2 mg, 27% yield).

^{1}H NMR (500 MHz, Chloroform-*d*) δ 4.67 (t, *J* = 3.9 Hz, 2H), 1.88 (q, *J* = 5.5 Hz, 4H), 1.60 (m,

2H), 1.45 - 1.35 (m, 2H).

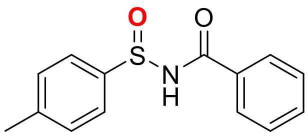

N-(p-tolylsulfinyl)benzamide (**4a**)[S4]

The desired pure product was purified using silica gel chromatography (PE : EA = 4 : 1) to give **4a** as a white solid (45.6 mg, 88% yield).

^{1}H NMR (400 MHz, Chloroform-*d*) δ 8.28 (br, 1H), 7.80 (d, *J* = 8.1 Hz, 2H), 7.68 (d, *J* = 8.0 Hz, 2H), 7.57 (t, *J* = 8.0 Hz, 1H), 7.45 (t, *J* = 7.7 Hz, 2H), 7.36 (d, *J* = 8.0 Hz, 2H), 2.44 (s, 3H).

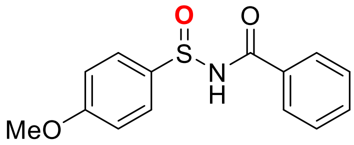

N-[(4-methoxyphenyl)sulfinyl)benzamide (**4b**)[S4]

The desired pure product was purified using silica gel chromatography (PE : EA = 4 : 1) to give **4b** as a white solid (50.6 mg, 92% yield).

^{1}H NMR (500 MHz, DMSO-d_6) δ 11.49 (br, 1H), 7.89 (d, *J* = 8.4 Hz, 2H), 7.71 (d, *J* = 8.6 Hz, 2H), 7.62 (t, *J* = 7.5 Hz, 1H), 7.50 (t, *J* = 7.7 Hz, 2H), 7.17 (d, *J* = 8.8 Hz, 2H), 3.84 (s, 3H).

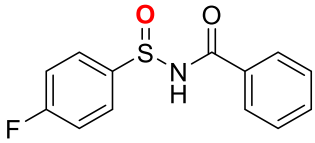

N-((4-fluorophenyl)sulfinyl)benzamide (**4c**)[S4]

The desired pure product was purified using silica gel chromatography (PE : EA = 5 : 1) to give **4c** as a white solid (42.0 mg, 80% yield).

^{1}H NMR (400 MHz, DMSO-d_6) δ 11.60 (br, 1H), 7.91 - 7.84 (m, 4H), 7.64 - 7.61 (m, 1H), 7.52 - 7.45 (m, 4H).

^{19}F NMR (376 MHz, DMSO-d_6) δ -109.3.

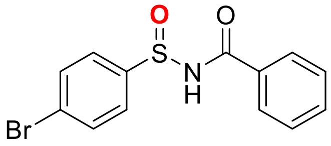

N-((4-bromophenyl)sulfinyl)benzamide (**4d**)[S5]

The desired pure product was purified using silica gel chromatography (PE : EA = 5 : 1) to give **4d** as a white solid (49.4 mg, 76% yield).

^{1}H NMR (400 MHz, DMSO-*d₆*) δ 11.62 (br, 1H), 7.90 (d, J = 7.7 Hz, 2H), 7.83 (d, J = 6.8 Hz, 2H), 7.73 (d, J = 6.8 Hz, 2H), 7.67 – 7.59 (m, 1H), 7.51 (t, J = 7.6 Hz, 2H).

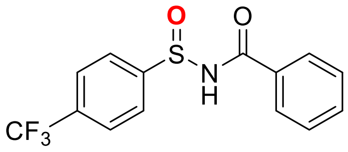

N-((4-(trifluoromethyl)phenyl)sulfinyl)benzamide (**4e**)[S4]

The desired pure product was purified using silica gel chromatography (PE : EA = 5 : 1) to give **4e** as a white solid (50.5 mg, 81% yield).

^{1}H NMR (400 MHz, Chloroform-*d*) δ 8.80 - 8.62 (m, 1H), 7.89 (d, *J* = 8.1 Hz, 2H), 7.80 (t, *J* = 8.7 Hz, 4H), 7.58 (t, *J* = 7.4 Hz, 1H), 7.46 (t, J = 7.6 Hz, 2H).

^{19}F NMR (471 MHz, Chloroform-*d*) δ -62.3.

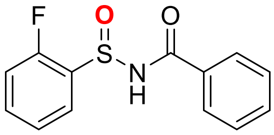

N-((2-fluorophenyl)sulfinyl)benzamide (**4f**)

The desired pure product was purified using silica gel chromatography (PE : EA = 4 : 1) to give **4f** as a white solid (27.0 mg, 52% yield).

^{1}H NMR (400 MHz, Chloroform-*d*) δ 8.47 (br, 1H), 8.02 **-** 7.92 (m, 1H), 7.80 (d, *J* = 7.7 Hz, 2H), 7.57 (t, *J* = 7.0 Hz, 2H), 7.42 (dt, *J* = 22.6, 7.6 Hz, 3H), 7.19 (t, *J* = 8.9 Hz, 1H).

^{13}C NMR (101 MHz, Chloroform-*d*) δ 167.2, 158.6 (d, *J* = 251.6 Hz), 134.3 (d, *J* = 7.7 Hz), 133.2, 131.6, 130.9 (d, *J* = 15.4 Hz), 128.9, 127.9, 126.8, 125.2 (d, *J* = 3.7 Hz), 116.5 (d, *J* = 20.0 Hz).

^{19}F NMR (471 MHz, DMSO-*d₆*) δ -114.1 (m).

HRMS (ESI) m/z: [M+H$^+$] Calcd. for $C_{13}H_{11}FNO_2S^+$: 264.0490; found: 264.0485.

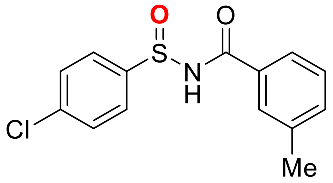

N-((4-chlorophenyl)sulfinyl)-3-methylbenzamide (**4g**)

The desired pure product was purified using silica gel chromatography (PE : EA = 5 : 1) to give **4g** as

a white solid (50.5 mg, 86% yield).

^{1}H NMR (400 MHz, Chloroform-*d*) δ 8.83 (br, 1H), 7.71 - 7.55 (m, 4H), 7.48 (d, *J* = 8.4 Hz, 2H), 7.40 - 7.28 (m, 2H), 2.37 (s, 3H).

^{13}C NMR (126 MHz, Chloroform-*d*) δ 167.7, 142.3, 139.0, 138.5, 134.2, 131.4, 129.8, 128.8, 128.8, 126.5, 125.1, 21.4.

HRMS (ESI) m/z: [M+H$^+$] Calcd. for $C_{14}H_{13}ClNO_2S^+$: 294.0351; found: 294.0354.

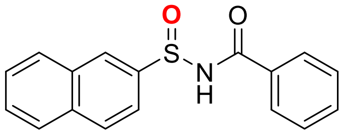

N-(naphthalen-2-ylsulfinyl)benzamide (**4h**)[S4]

The desired pure product was purified using silica gel chromatography (PE : EA = 5 : 1) to give **4h** as a white solid (43.3 mg, 73% yield).

^{1}H NMR (500 MHz, DMSO-*d*$_6$) δ 11.67 (br, 1H), 8.44 (s, 1H), 8.16 (dd, *J* = 15.1, 7.9 Hz, 2H), 8.07 (d, *J* = 7.0 Hz, 1H), 7.95 - 7.87 (m, 2H), 7.79 (dd, *J* = 8.6, 1.9 Hz, 1H), 7.70 - 7.61 (m, 3H), 7.50 (t, *J* = 7.8 Hz, 2H).

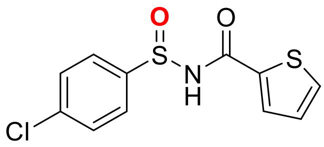

N-((4-chlorophenyl)sulfinyl)furan-2-carboxamide (**4i**)

The desired pure product was purified using silica gel chromatography (PE : EA = 5 : 1) to give **4i** as a white solid (34.3 mg, 64% yield).

^{1}H NMR (400 MHz, DMSO-*d*$_6$) δ 11.64 (br, 1H), 7.97 (dd, *J* = 14.6, 4.4 Hz, 2H), 7.80 (d, *J* = 8.2 Hz, 2H), 7.71 (d, *J* = 8.3 Hz, 2H), 7.20 (t, *J* = 4.4 Hz, 1H).

^{13}C NMR (101 MHz, DMSO-*d*$_6$) δ 162.8, 142.9, 137.0, 136.8, 134.8, 132.4, 129.7, 128.9, 127.8.

HRMS (ESI) m/z: [M+H$^+$] Calcd. for $C_{11}H_9ClNO_3S^+$: 285.9758; found: 285.9753.

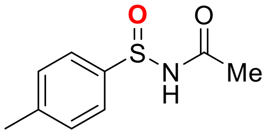

N-(*p*-tolylsulfinyl)acetamide (**4j**)[S6]

The desired pure product was purified using silica gel chromatography (PE : EA = 2 : 1) to give **4j** as a white solid (27.2 mg, 69% yield).

^{1}H NMR (400 MHz, Chloroform-*d*) δ 7.61 (d, *J* = 8.0 Hz, 2H), 7.52 (br, 1H) 7.36 (d, *J* = 7.9 Hz, 2H), 2.44 (s, 3H), 2.20 (s, 3H).

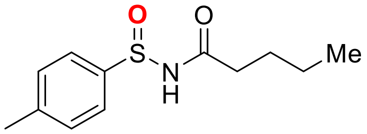

N-(*p*-tolylsulfinyl)pentanamide (**4k**)

The desired pure product was purified using silica gel chromatography (PE : EA = 2 : 1) to give **4k** as a white solid (35.4 mg, 74% yield).

^{1}H NMR (400 MHz, Chloroform-*d*) δ 7.87 (br, 1H), 7.55 (d, *J* = 7.9 Hz, 2H), 7.33 (d, *J* = 7.9 Hz, 2H), 2.43 (s, 3H), 2.41 - 2.31 (m, 2H), 1.65 (t, *J* = 7.7 Hz, 2H), 1.41 - 1.32 (m, 2H), 0.91 (t, *J* = 7.3 Hz, 3H).

^{13}C NMR (101 MHz, Chloroform-*d*) δ 173.8, 142.6, 140.5, 130.1, 124.7, 26.8, 22.2, 21.5, 13.7.

HRMS (ESI) m/z: [M+H$^+$] Calcd. for $C_{12}H_{18}NO_2S^+$: 240.1053; found: 240.1049.

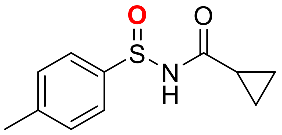

N-(*p*-tolylsulfinyl)cyclopropanecarboxamide (**4l**)

The desired pure product was purified using silica gel chromatography (PE : EA = 2 : 1) to give **4l** as a white solid (31.7 mg, 71% yield).

^{1}H NMR (400 MHz, Chloroform-*d*) δ 8.44 (br, 1H), 7.55 (d, *J* = 7.9 Hz, 2H), 7.33 (d, *J* = 7.9 Hz, 2H), 2.43 (s, 3H), 1.62 (br, 1H), 1.12 (m, 2H), 0.92 (m, 2H).

^{13}C NMR (101 MHz, Chloroform-*d*) δ 174.8, 142.5, 140.4, 130.0, 124.8, 21.5, 14.5, 9.6.

HRMS (ESI) m/z: [M+H$^+$] Calcd. for $C_{11}H_{14}NO_2S^+$: 224.0740; found: 224.0736.

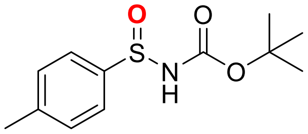

tert-butyl (*p*-tolylsulfinyl)carbamate (**4m**)[S4]

The desired pure product was purified using silica gel chromatography (PE : EA = 3 : 1) to give **4m** as a white solid (35.3 mg, 69% yield).

^{1}H NMR (400 MHz, Chloroform-*d*) δ 7.61 (d, *J* = 8.0 Hz, 2H), 7.33 (d, *J* = 7.9 Hz, 2H), 6.79 (br, 1H), 2.42 (s, 3H), 1.50 (s, 9H).

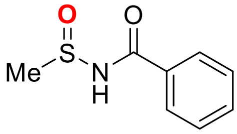

N-(methylsulfinyl)benzamide (**4n**)

The desired pure product was purified using silica gel chromatography (PE : EA = 2 : 1) to give **4n** as a white solid (33.2 mg, 91% yield).

^{1}H NMR (400 MHz, Chloroform-*d*) δ 10.09 (br, 1H), 7.86 (d, *J* = 7.7 Hz, 2H), 7.53 (t, *J* = 7.4 Hz, 1H), 7.41 (t, *J* = 7.6 Hz, 2H), 2.90 (s, 3H).

^{13}C NMR (101 MHz, Chloroform-*d*) δ 167.4, 133.1, 131.3, 128.7, 128.2, 41.1.

HRMS (ESI) m/z: [M+H^{+}] Calcd. for $C_8H_{10}NO_2S^+$: 184.0427; found: 184.0425.

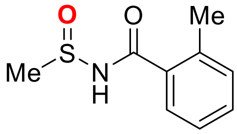

2-methyl-*N*-(methylsulfinyl)benzamide (**4o**)

The desired pure product was purified using silica gel chromatography (PE : EA = 2 : 1) to give **4o** as a white solid (33.7 mg, 86% yield).

^{1}H NMR (400 MHz, Chloroform-*d*) δ 9.21 (br, 1H), 7.44 (d, *J* = 7.6 Hz, 1H), 7.41 **-** 7.32 (m, 1H), 7.27 **-** 7.21 (m, 2H), 2.81 (s, 3H), 2.45 (s, 3H).

^{13}C NMR (126 MHz, Chloroform-*d*) δ 169.9, 137.7, 132.6, 131.6, 131.5, 127.7, 125.9, 41.5, 20.2.

HRMS (ESI) m/z: [M+H^{+}] Calcd. for $C_9H_{12}NO_2S^+$: 198.0584; found: 198.0582.

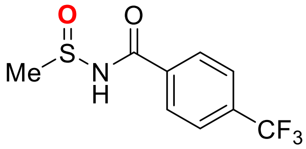

N-(methylsulfinyl)-4-(trifluoromethyl)benzamide (**4p**)

The desired pure product was purified using silica gel chromatography (PE : EA = 1 : 1) to give **4p** as a white solid (38.3 mg, 76% yield).

^{1}H NMR (400 MHz, Chloroform-*d*) δ 10.29 (br, 1H), 7.98 (d, *J* = 8.1 Hz, 2H), 7.69 (d, *J* = 8.1 Hz, 2H), 2.95 (s, 3H).

^{13}C NMR (126 MHz, Chloroform-*d*) δ 166.2, 134.7 (q, *J* = 32.6 Hz), 128.8, 125.9 (q, *J* = 3.6 Hz), 124.6, 123.5 (q, *J* = 272.6 Hz), 41.3.

^{19}F NMR (471 MHz, Chloroform-*d*) δ -63.1.

HRMS (ESI) m/z: [M+H$^+$] Calcd. for $C_9H_9F_3NO_2S^+$: 252.0301; found: 252.0297.

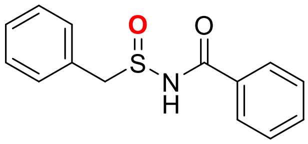

N-(benzylsulfinyl)benzamide (**4q**)

The desired pure product was purified using silica gel chromatography (PE : EA = 4 : 1) to give **4q** as a white solid (36.1 mg, 70% yield).

^{1}H NMR (400 MHz, Chloroform-*d*) δ 9.16 (br, 1H), 7.77 (d, *J* = 7.7 Hz, 2H), 7.54 (t, *J* = 7.4 Hz, 1H), 7.41 (t, *J* = 7.6 Hz, 2H), 7.36 - 7.24 (m, 5H), 4.36 (d, *J* = 13.0 Hz, 1H), 4.23 (d, *J* = 13.0 Hz, 1H).

^{13}C NMR (101 MHz, Chloroform-*d*) δ 167.2, 133.1, 131.6, 130.6, 129.0, 128.8, 128.7, 128.3, 128.0, 60.7.

HRMS (ESI) m/z: [M+H$^+$] Calcd. for $C_{14}H_{14}NO_2S^+$: 260.0740; found: 260.0736.

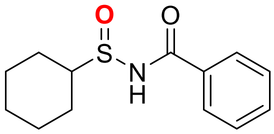

N-(cyclohexylsulfinyl)benzamide (**4r**)[S7]

The desired pure product was purified using silica gel chromatography (PE : EA = 4:1) to give **4r** as a white solid (28.1 mg, 56% yield).

^{1}H NMR (400 MHz, Chloroform-*d*) δ 9.14 (br, 1H), 7.88 (d, *J* = 7.6 Hz, 2H), 7.57 (t, *J* = 7.2 Hz, 1H), 7.46 (t, *J* = 7.6 Hz, 2H), 3.06 (tt, *J* = 11.4, 3.8 Hz, 1H), 2.04 - 1.94 (m, 2H), 1.89 - 1.80 (m, 2H), 1.72 - 1.63 (m, 2H), 1.39 - 1.22 (m, 4H).

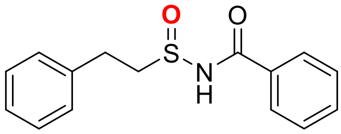

N-(phenethylsulfinyl)benzamide (**4s**)

The desired pure product was purified using silica gel chromatography (PE : EA = 5 : 1) to give **4s** as a white solid (43.9 mg, 81% yield).

^{1}H NMR (400 MHz, Chloroform-*d*) δ 9.53 (s, 1H), 7.76 (d, *J* = 7.7 Hz, 2H), 7.54 (t, *J* = 7.4 Hz, 1H), 7.41 (t, *J* = 7.6 Hz, 2H), 7.32 - 7.22 (m, 3H), 7.20 - 7.07 (m, 2H), 3.39 (t, *J* = 7.6 Hz, 2H), 3.10 - 2.95 (m, 2H).

^{13}C NMR (101 MHz, Chloroform-*d*) δ 167.2, 138.1, 133.1, 131.4, 128.9, 128.7, 128.5, 128.1, 127.0,

55.7, 28.4.

HRMS (ESI) m/z: [M+H$^+$] Calcd. for $C_{15}H_{16}NO_2S^+$: 274.0897; found: 274.0892.

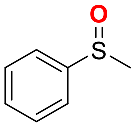

(methylsulfinyl)benzene (**6a**)[S30]

The desired pure product was purified using silica gel chromatography (PE : EA = 5 : 1) to give **6a** as a colorless oil (69.4 mg, 99% yield).

^{1}H NMR (500 MHz, Chloroform-*d*) δ 7.73 - 7.57 (m, 2H), 7.54 - 7.41 (m, 3H), 2.68 (s, 3H).

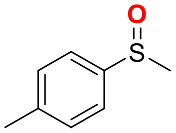

1-methyl-4-(methylsulfinyl)benzene (**6b**)[S30]

The desired pure product was purified using silica gel chromatography (PE : EA = 5 : 1) to give **6b** as a colorless oil (76.4 mg, 99% yield).

^{1}H NMR (500 MHz, Chloroform-*d*) δ 7.50 (d, *J* = 8.3 Hz, 2H), 7.29 (d, *J* = 7.9 Hz, 2H), 2.67 (s, 3H), 2.38 (s, 3H).

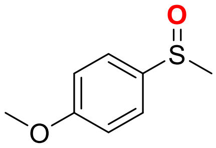

1-methoxy-4-(methylsulfinyl)benze (**6c**)[S30]

The desired pure product was purified using silica gel chromatography (PE : EA = 5 : 1) to give **6c** as a colorless oil (80.0 mg, 94% yield).

^{1}H NMR (500 MHz, Chloroform-*d*) δ 7.77 (dd, *J* = 7.6, 1.8 Hz, 1H), 7.43 - 7.37 (m, 1H), 7.13 (t, *J* = 7.5 Hz, 1H), 6.88 (d, *J* = 7.3 Hz, 1H), 3.84 (s, 3H), 2.72 (s, 3H).

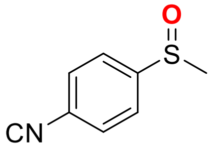

4-(methylsulfinyl)benzonitrile (**6d**)[S30]

The desired pure product was purified using silica gel chromatography (PE : EA = 3 : 1) to give **6d** as

a white solid (81.7 mg, 99% yield).

^{1}H NMR (500 MHz, Chloroform-*d*) δ 7.80 (d, *J* = 8.5 Hz, 2H), 7.74 (d, *J* = 8.5 Hz, 2H), 2.73 (s, 3H).

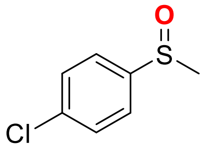

1-chloro-4-(methylsulfinyl)benzene (**6e**)[S30]

The desired pure product was purified using silica gel chromatography (PE : EA = 3 : 1) to give **4f** as a colorless oil (86.4 mg, 99% yield).

^{1}H NMR (500 MHz, Chloroform-*d*) δ 7.55 (d, *J* = 8.6 Hz, 2H), 7.46 (d, *J* = 8.6 Hz, 2H), 2.67 (s, 3H)

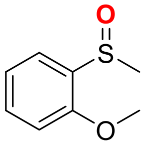

1-methoxy-2-(methylsulfinyl)benzene (**6f**)[S30]

The desired pure product was purified using silica gel chromatography (PE : EA = 5 : 1) to give **6g** as a colorless oil (88.7 mg, 99% yield).

^{1}H NMR (500 MHz, Chloroform-*d*) δ 7.59 - 7.53 (m, 2H), 7.02 - 6.96 (m, 2H), 3.81 (s, 3H), 2.66 (s, 3H).

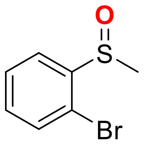

1-bromo-2-(methylsulfinyl)benzene (**6g**)[S30]

The desired pure product was purified using silica gel chromatography (PE : EA = 3:1) to give **6h** as a colorless oil (108.8 mg, 99% yield).

^{1}H NMR (500 MHz, Chloroform-*d*) δ 7.90 (dd, *J* = 7.8, 1.7 Hz, 1H), 7.56- 7.49 (m, 2H), 7.33 (td, *J* = 7.7, 1.7 Hz, 1H), 2.77 (s, 3H).

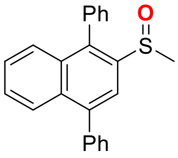

2-(methylsulfinyl)-1,4-diphenylnaphthalene (**6h**)[S31]

The desired pure product was purified using silica gel chromatography (PE : EA = 3 : 1) to give **6i** as

a white solid (37.7 mg, 22% yield).

^{1}H NMR (400 MHz, Chloroform-*d*) δ 8.13 (s, 1H), 8.03 (d, *J* = 8.4 Hz, 1H), 7.61 - 7.44 (m, 12H), 7.30 - 7.26 (m, 1H), 2.57 (s, 3H).

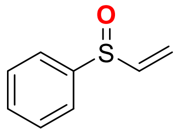

(vinylsulfinyl)benzene (**6i**)[S32]

The desired pure product was purified using silica gel chromatography (PE : EA = 3 : 1) to give **6l** as a colorless oil (62.3 mg, 82% yield).

^{1}H NMR (400 MHz, Chloroform-*d*) δ 7.51 (d, *J* = 7.4 Hz, 2H), 7.43 - 7.30 (m, 3H), 6.50 (m, 1H), 6.09 (dd, *J* = 16.3, 6.1 Hz, 1H), 5.77 (t, *J* = 7.9 Hz, 1H).

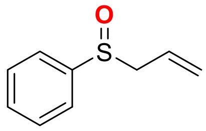

(allylsulfinyl)benzene (**6j**)[S30]

The desired pure product was purified using silica gel chromatography (PE : EA = 3 : 1) to give **6m** as a colorless oil (80.7 mg, 96% yield).

^{1}H NMR (500 MHz, Chloroform-*d*) δ 7.60 - 7.56 (m, 2H), 7.52 - 7.46 (m, 3H), 5.62 (ddt, *J* = 17.5, 10.2, 7.5 Hz, 1H), 5.31 (dd, *J* = 10.2, 1.3 Hz, 1H), 5.17 (dq, *J* = 17.0, 1.3 Hz, 1H), 3.58 - 3.53 (m, 1H), 3.48 - 3.46 (m, 1H).

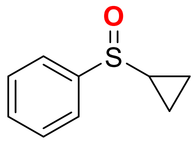

(cyclopropylsulfinyl)benzene (**6k**)[S32]

The desired pure product was purified using silica gel chromatography (PE : EA = 3 : 1) to give **6n** as a colorless oil (77.3 mg, 93% yield).

^{1}H NMR (500 MHz, Chloroform-*d*) δ 7.71 - 7.59 (m, 2H), 7.55 - 7.44 (m, 3H), 2.25 (tt, *J* = 7.9, 4.9 Hz, 1H), 1.33 - 1.25 (m, 1H), 1.05 - 0.91 (m, 1H), 0.96 - 0.89 (m, 2H).

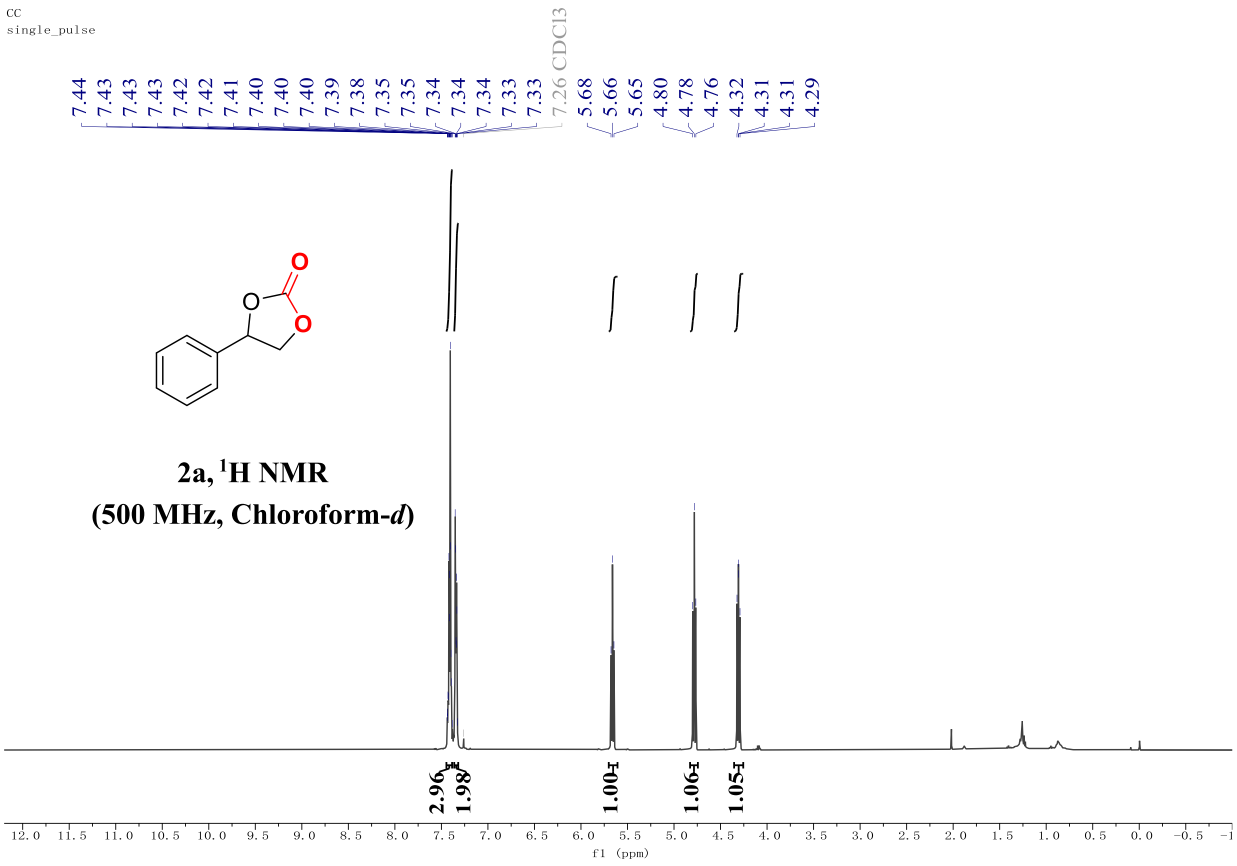
CC
single_pulse
7.44 7.43 7.43 7.43 7.42 7.42 7.41 7.40 7.40 7.40 7.39 7.38 7.35 7.35 7.34 7.34 7.34 7.33 7.33 7.26 CDCl3 5.68 5.66 5.65 4.80 4.78 4.76 4.32 4.31 4.31 4.29
O
O
O
2a, 1H NMR
(500 MHz, Chloroform-d)
2.96 1.98 1.00 1.06 1.05
f1 (ppm)

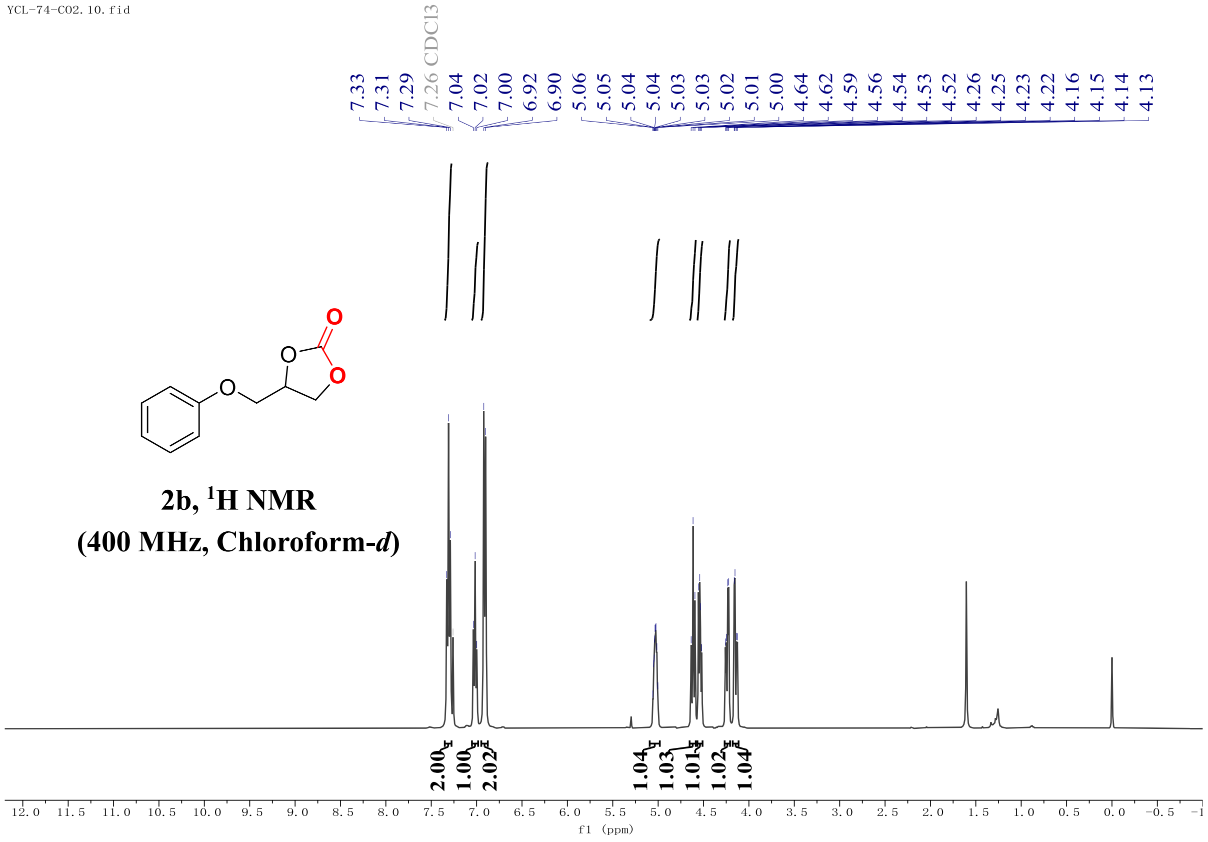
YCL-74-C02.10.fid
7.33 7.31 7.29 7.26 CDCl3 7.04 7.02 7.00 6.92 6.90 5.06 5.05 5.04 5.04 5.03 5.03 5.02 5.01 5.00 4.64 4.62 4.59 4.56 4.54 4.53 4.52 4.26 4.25 4.23 4.22 4.16 4.15 4.14 4.13
O
O
O
O
2b, 1H NMR
(400 MHz, Chloroform-d)
2.00 1.00 2.02 1.04 1.03 1.01 1.02 1.04
f1 (ppm)

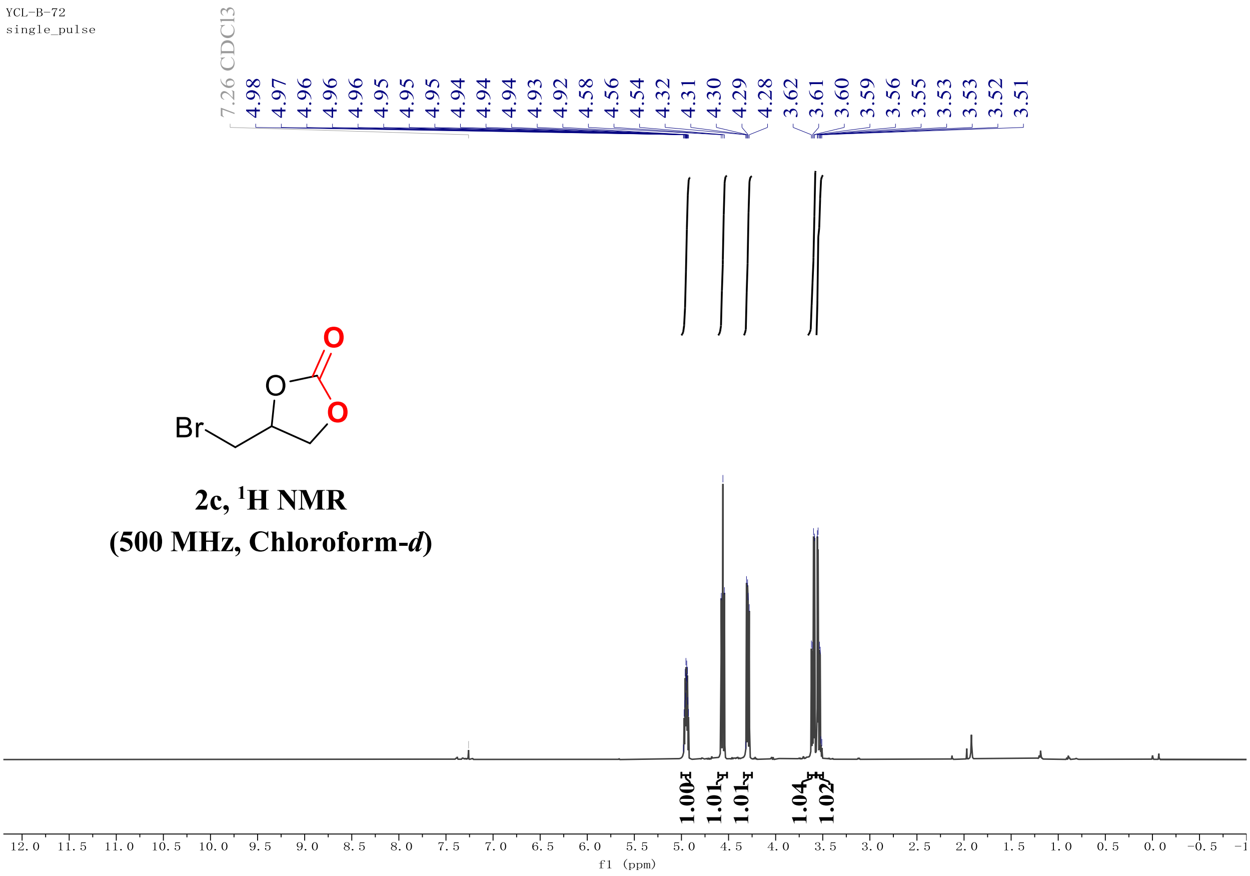

2c, ^{1}H NMR (500 MHz, Chloroform-*d*)

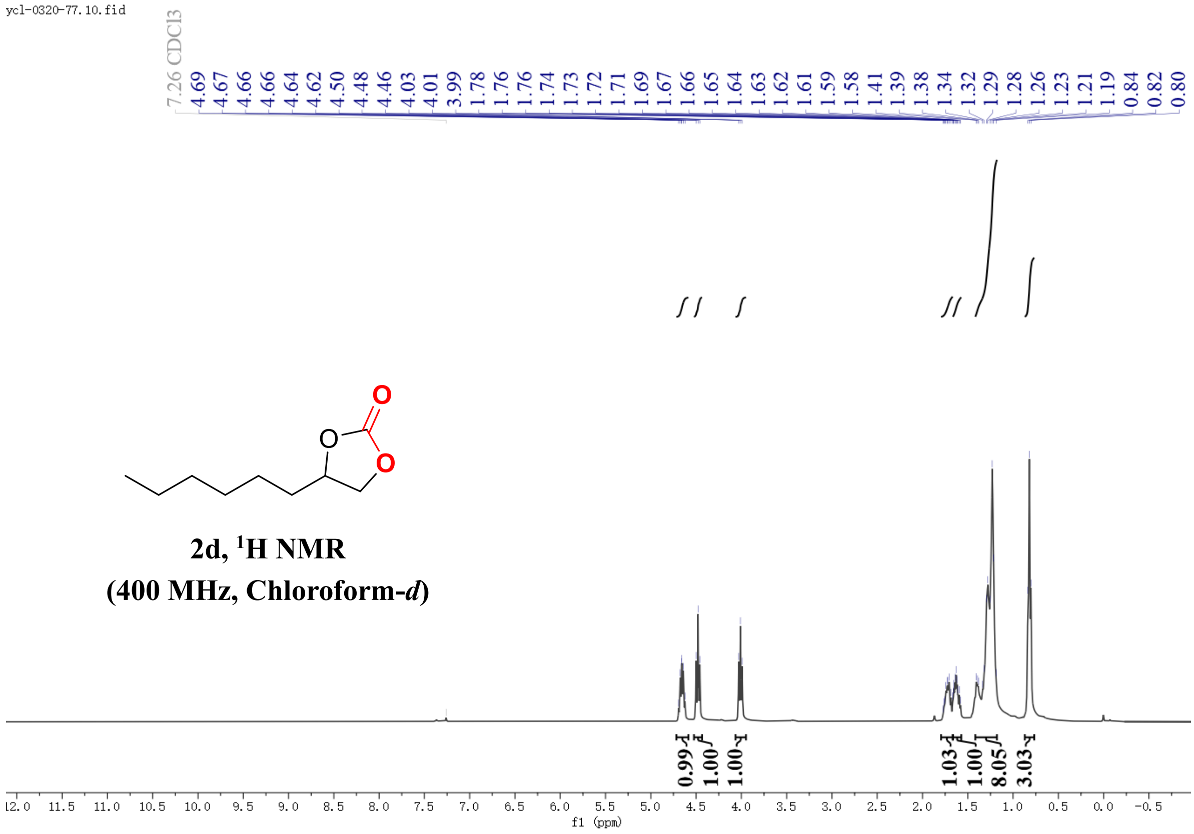

2d, ^{1}H NMR (400 MHz, Chloroform-*d*)

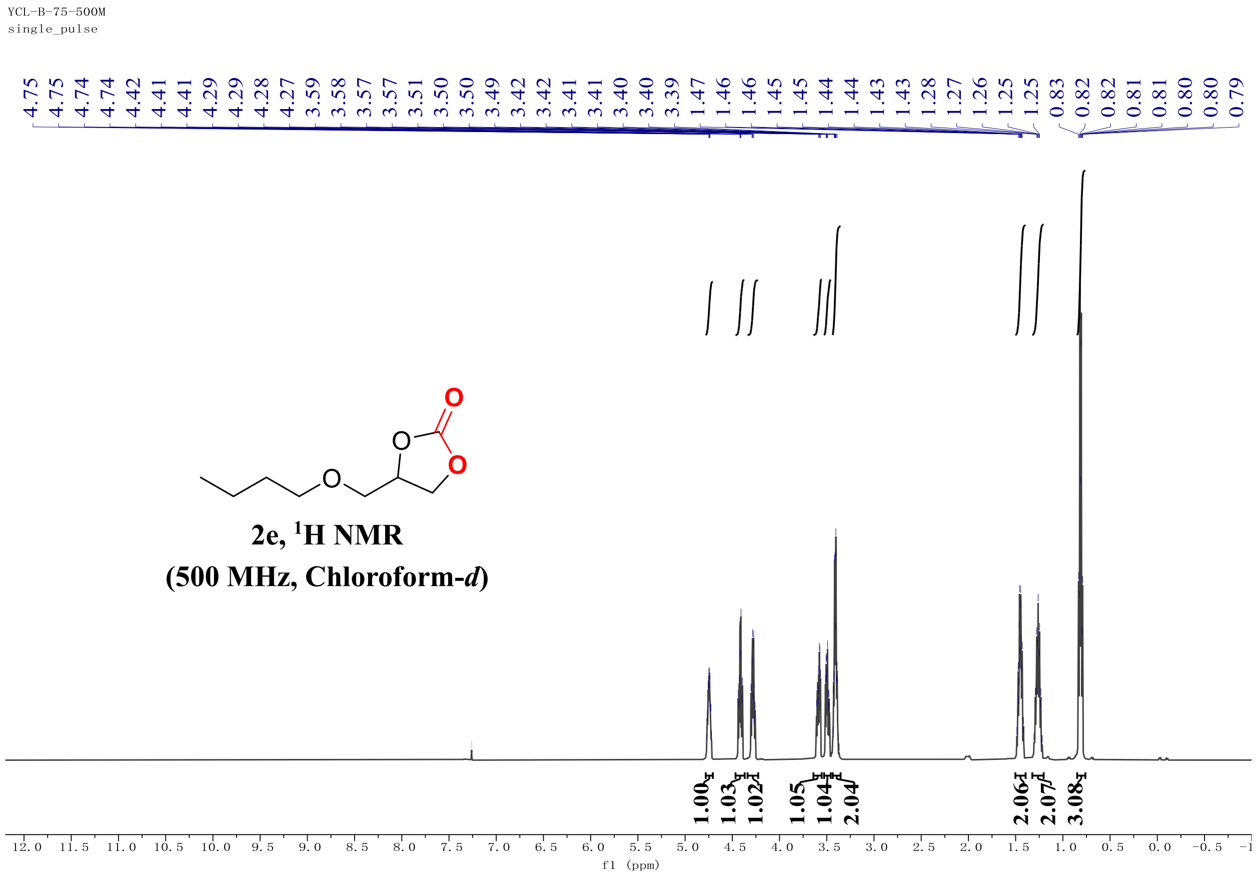
YCL-B-75-500M
single_pulse
4.75 4.75 4.74 4.74 4.42 4.41 4.41 4.29 4.29 4.28 4.27 3.59 3.58 3.57 3.57 3.51 3.50 3.50 3.49 3.42 3.42 3.41 3.41 3.40 3.40 3.39 1.47 1.46 1.46 1.45 1.45 1.44 1.44 1.43 1.43 1.28 1.27 1.26 1.25 1.25 0.83 0.82 0.82 0.81 0.81 0.80 0.80 0.79
2e, ¹H NMR
(500 MHz, Chloroform-d)
1.00 1.03 1.02 1.05 1.04 2.04 2.06 2.07 3.08
f1 (ppm)

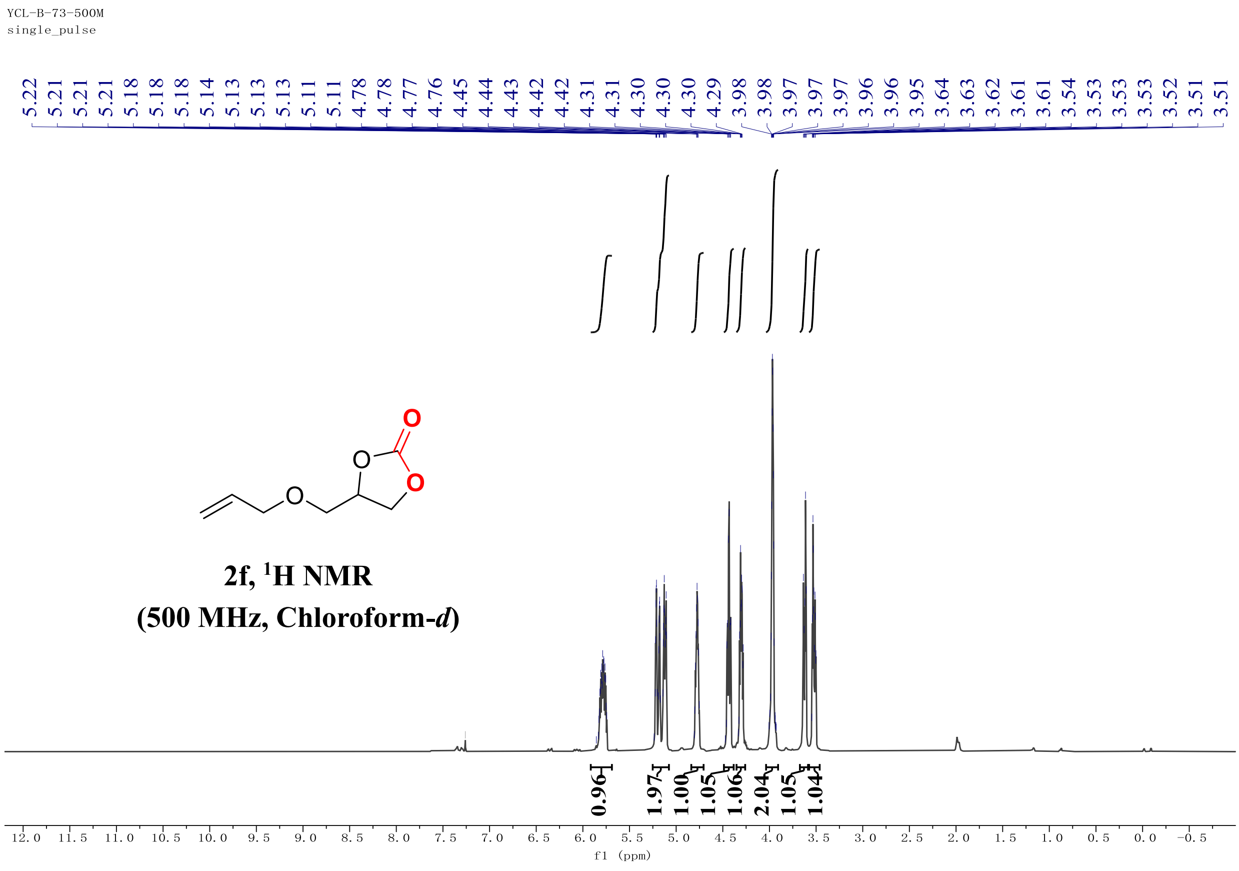
YCL-B-73-500M
single_pulse
5.22 5.21 5.21 5.21 5.18 5.18 5.18 5.14 5.13 5.13 5.13 5.11 5.11 4.78 4.78 4.77 4.76 4.45 4.44 4.43 4.42 4.42 4.31 4.31 4.30 4.30 4.30 4.29 3.98 3.98 3.97 3.97 3.97 3.96 3.96 3.95 3.64 3.63 3.62 3.61 3.61 3.54 3.53 3.53 3.53 3.52 3.51 3.51
2f, ¹H NMR
(500 MHz, Chloroform-d)
0.96 1.97 1.00 1.05 1.06 2.04 1.05 1.04
f1 (ppm)

YCL-B-76-500M
single_pulse

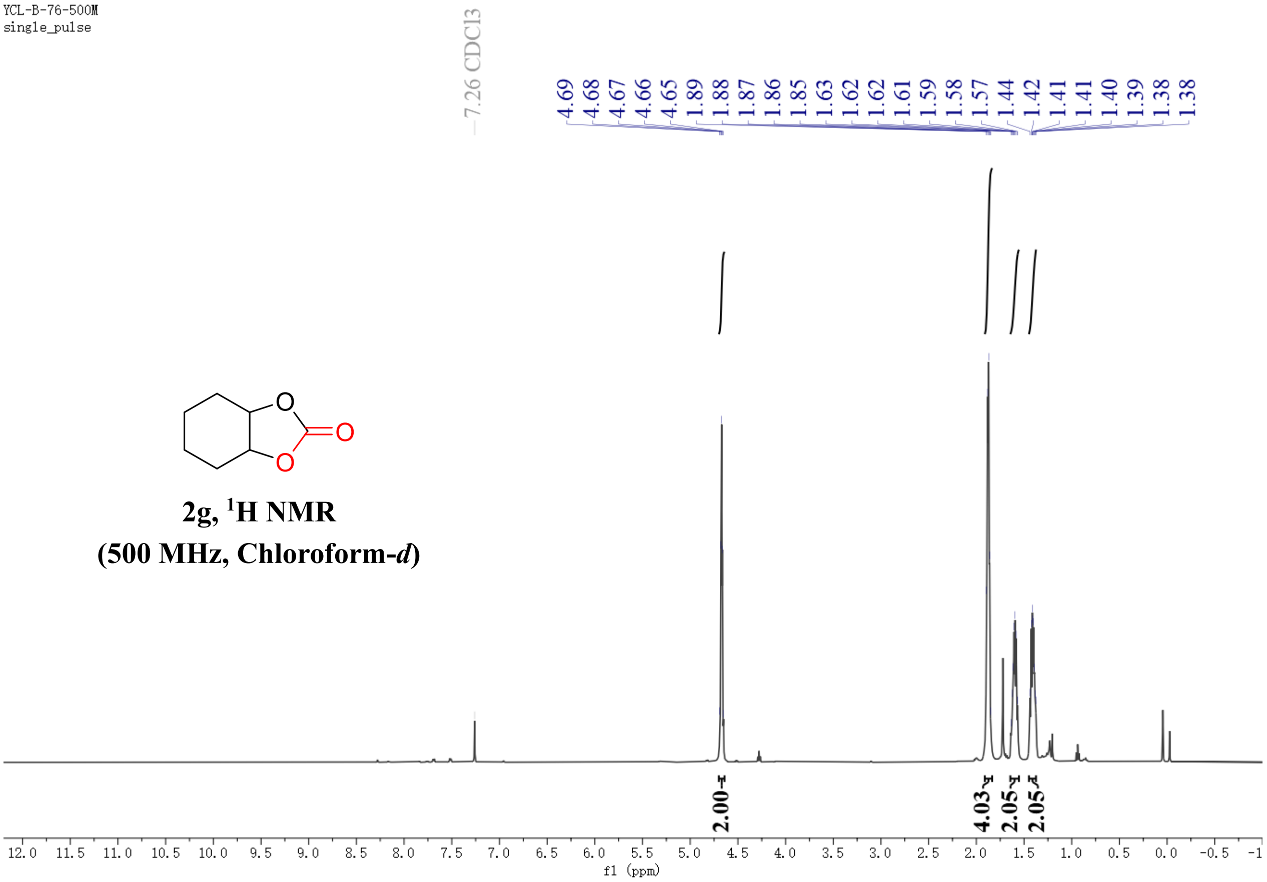

2g, ¹H NMR
(500 MHz, Chloroform-*d*)

Y-51-13.10.fid

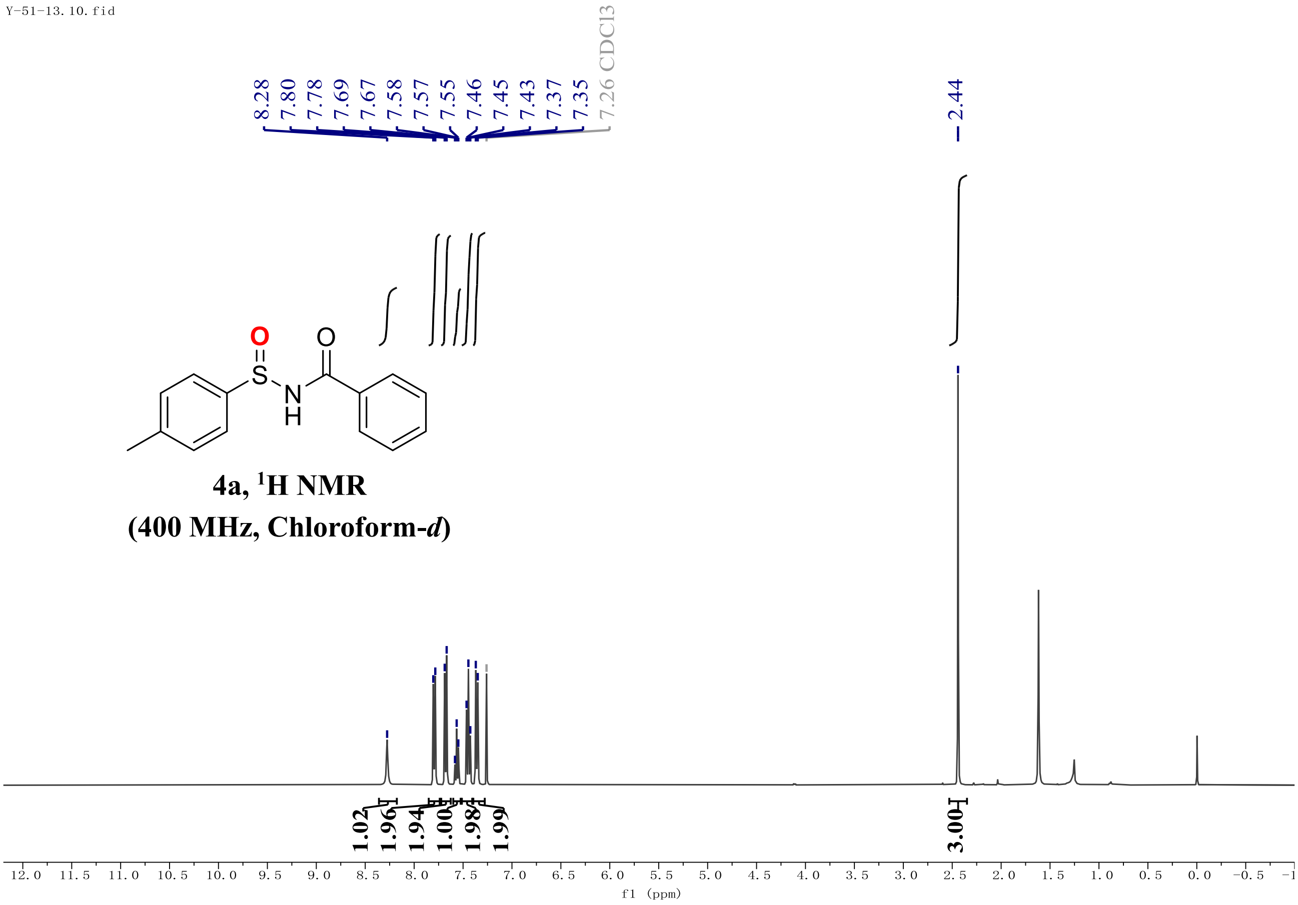

4a, ¹H NMR
(400 MHz, Chloroform-*d*)

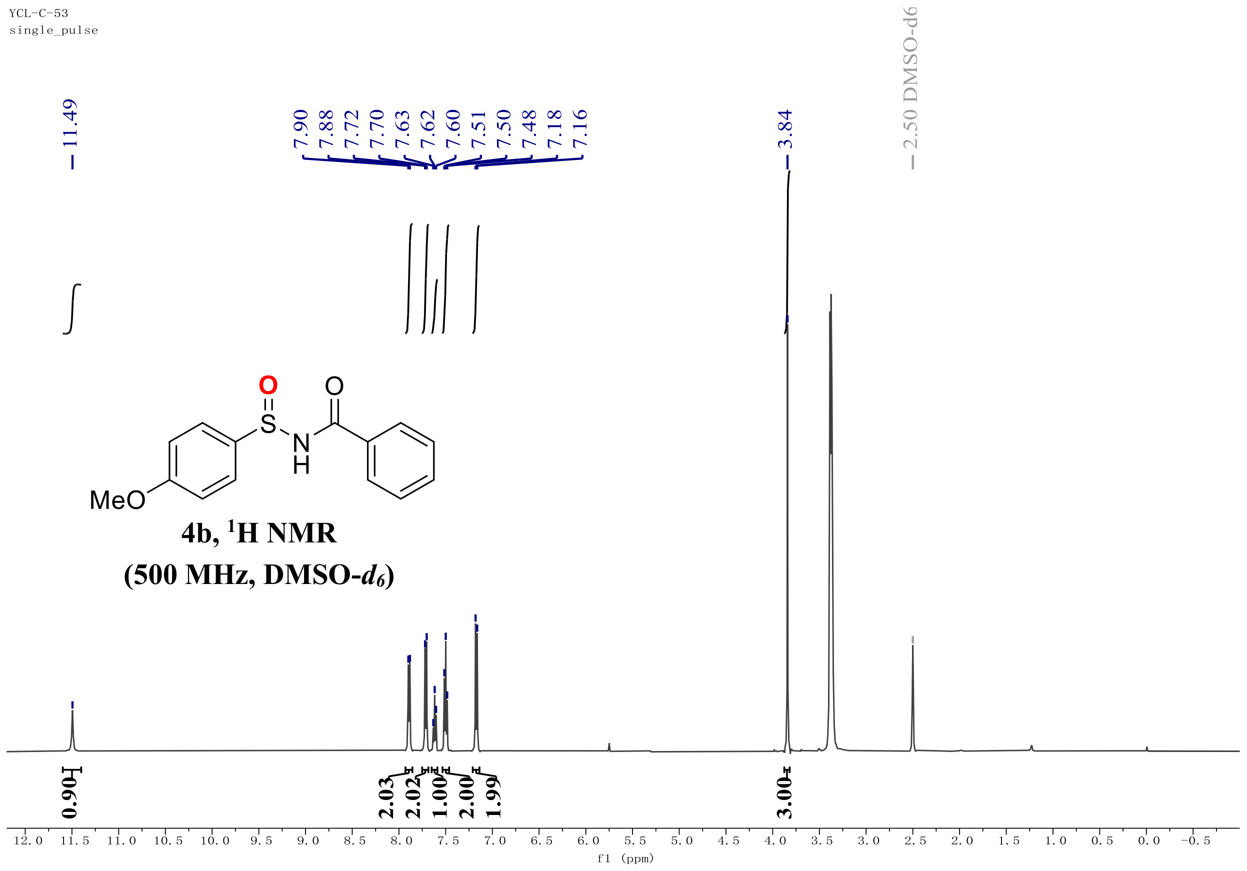
YCL-C-53
single_pulse
11.49
7.90
7.88
7.72
7.70
7.63
7.62
7.60
7.51
7.50
7.48
7.18
7.16
3.84
2.50 DMSO-d6
MeO
4b, 1H NMR
(500 MHz, DMSO-d6)
0.90
2.03
2.02
1.00
2.00
1.99
3.00
f1 (ppm)

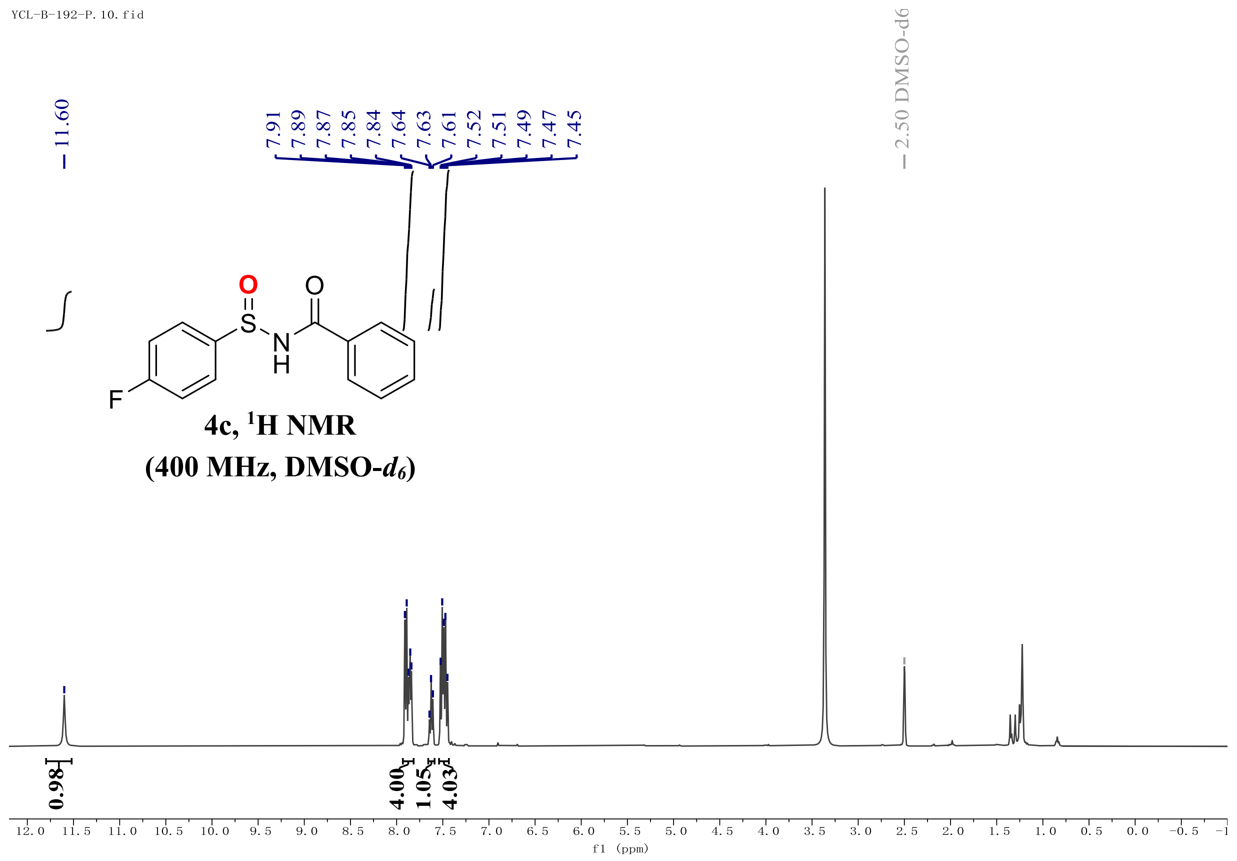
YCL-B-192-P.10.fid
11.60
7.91
7.89
7.87
7.85
7.84
7.64
7.63
7.61
7.52
7.51
7.49
7.47
7.45
2.50 DMSO-d6
F
4c, 1H NMR
(400 MHz, DMSO-d6)
0.98
4.00
1.05
4.03
f1 (ppm)

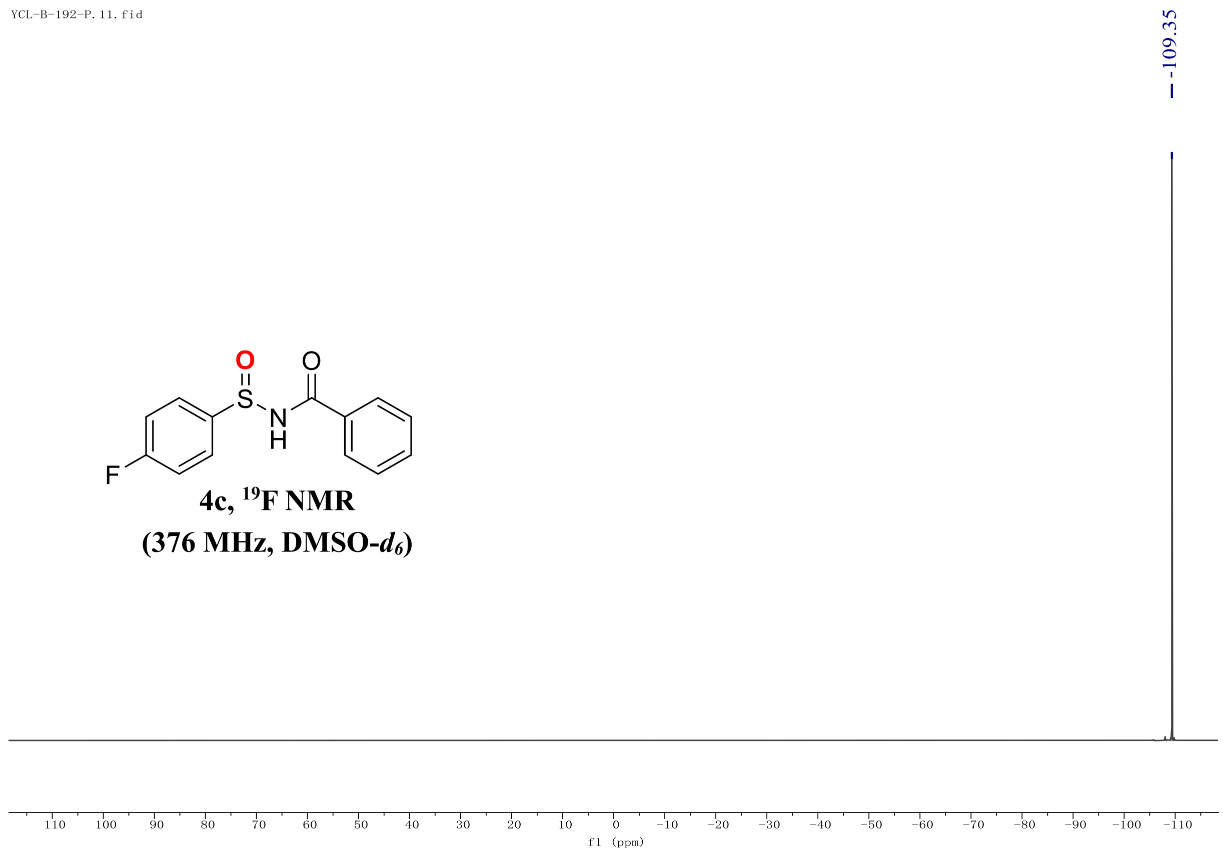
YCL-B-192-P.11.fid
-109.35
F
4c, ¹⁹F NMR
(376 MHz, DMSO-d6)
f1 (ppm)

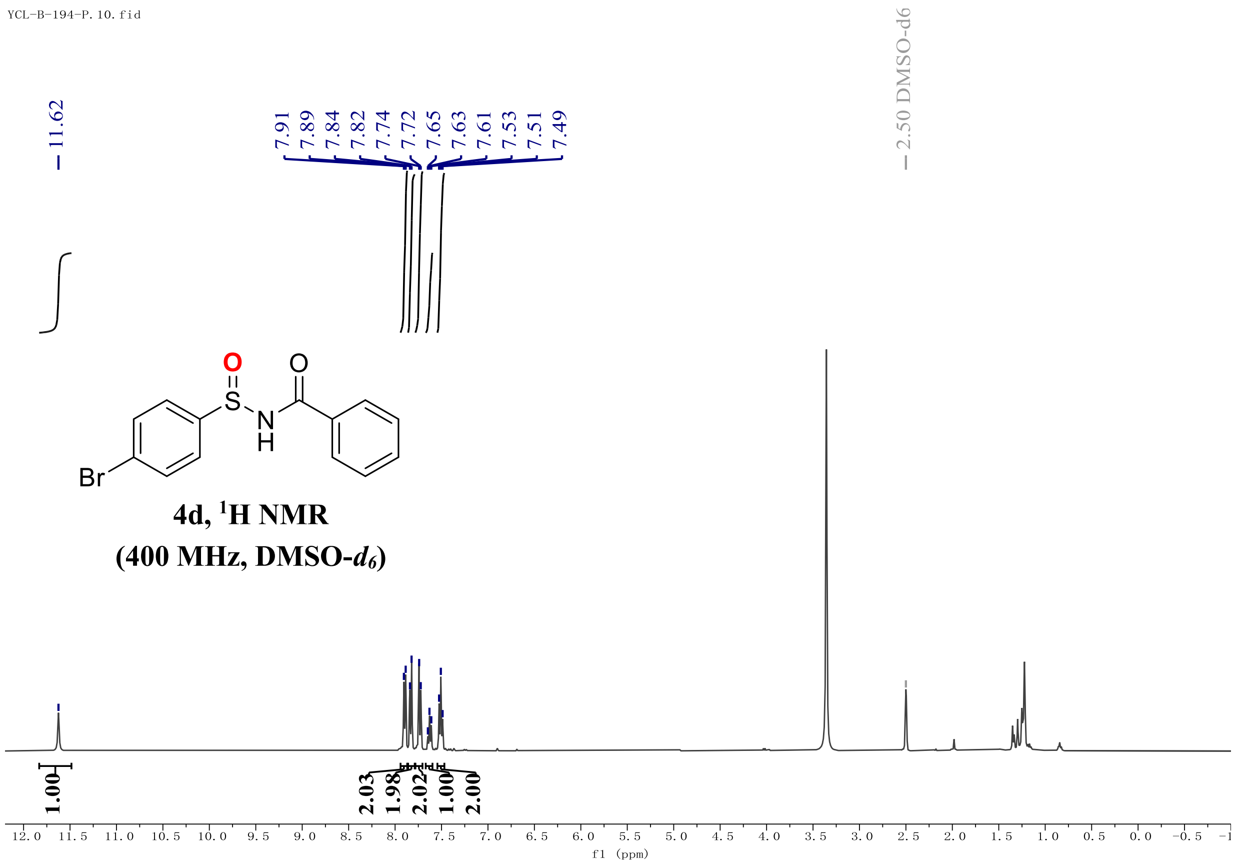
YCL-B-194-P.10.fid
11.62
7.91
7.89
7.84
7.82
7.74
7.72
7.65
7.63
7.61
7.53
7.51
7.49
2.50 DMSO-d6
Br
4d, ¹H NMR
(400 MHz, DMSO-d6)
1.00
2.03
1.98
2.02
1.00
2.00
f1 (ppm)

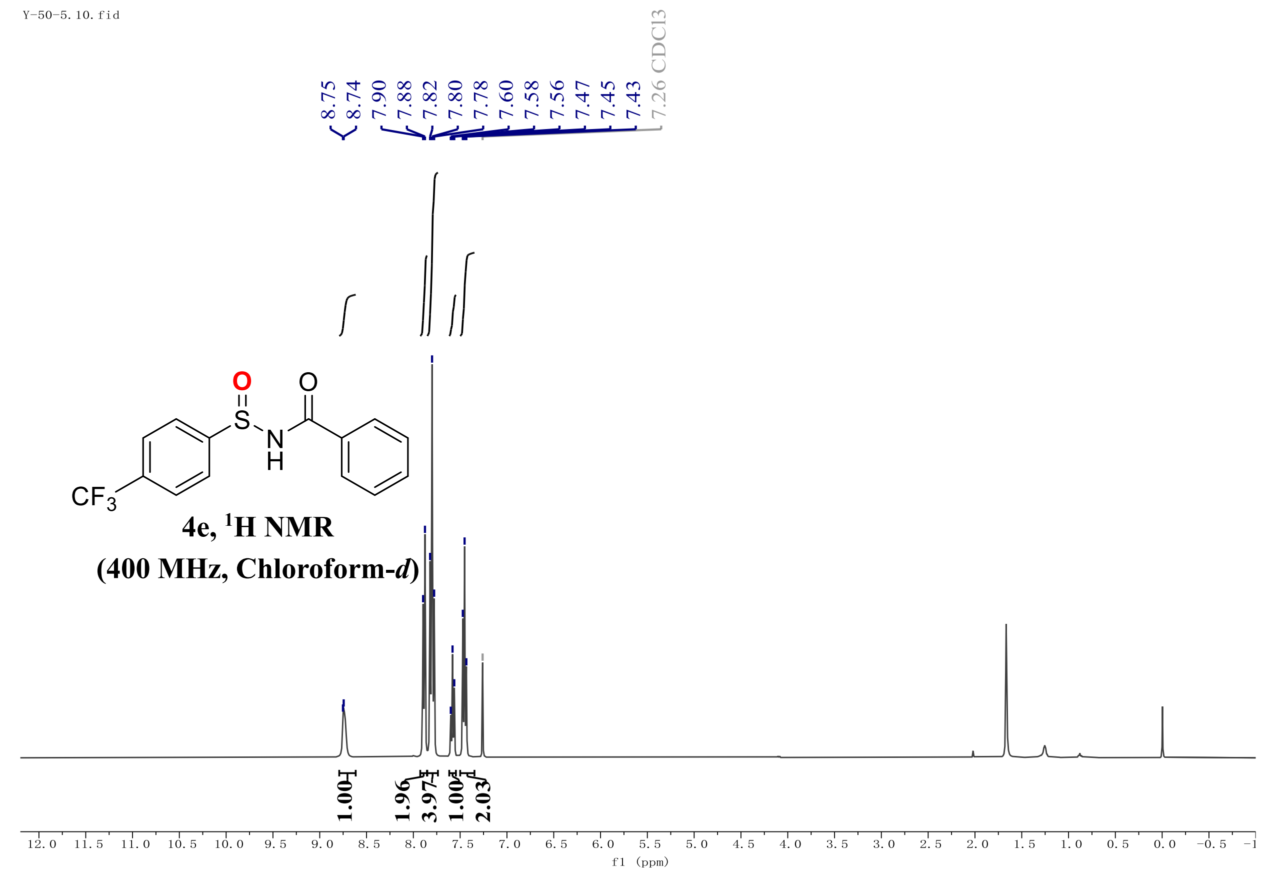

4e, ^{1}H NMR
(400 MHz, Chloroform-*d*)

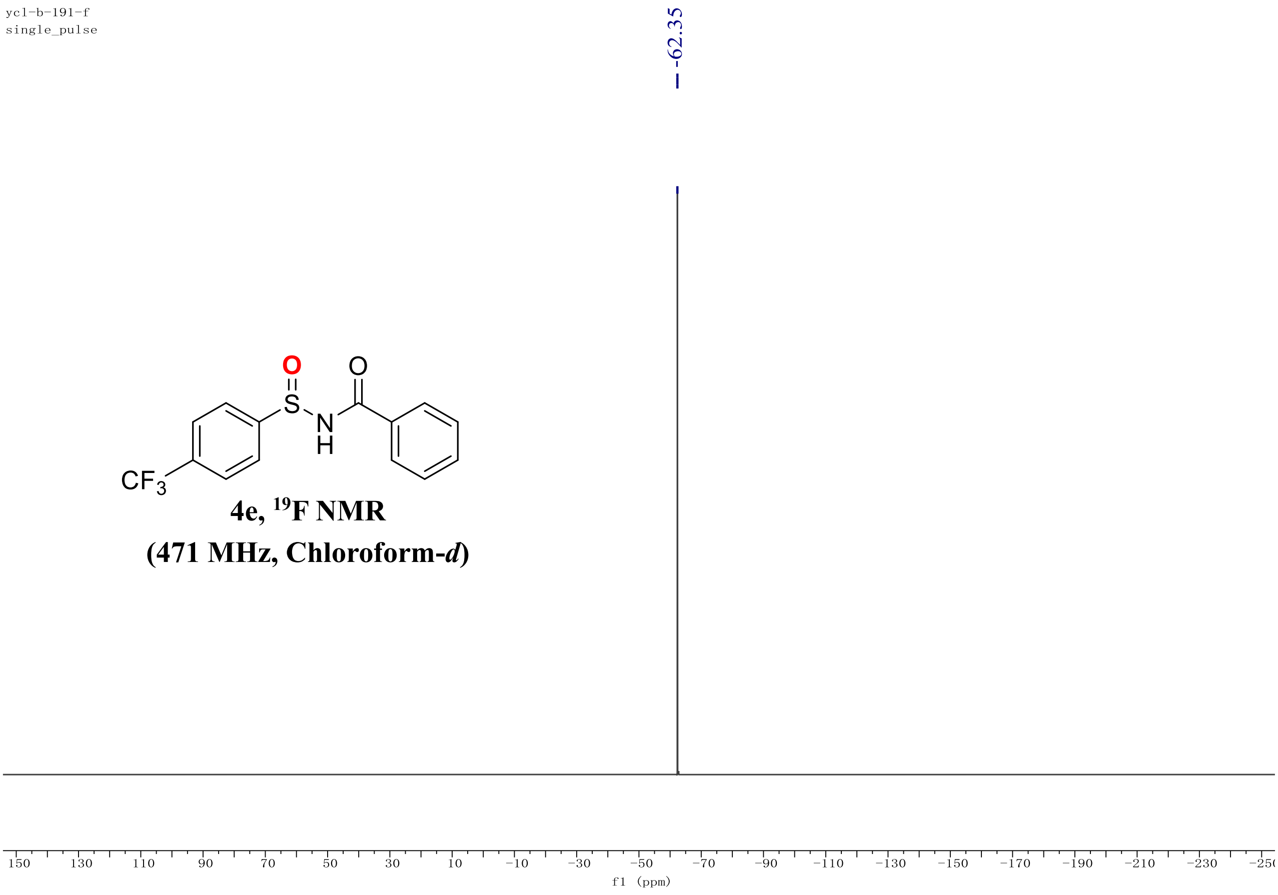

4e, ^{19}F NMR
(471 MHz, Chloroform-*d*)

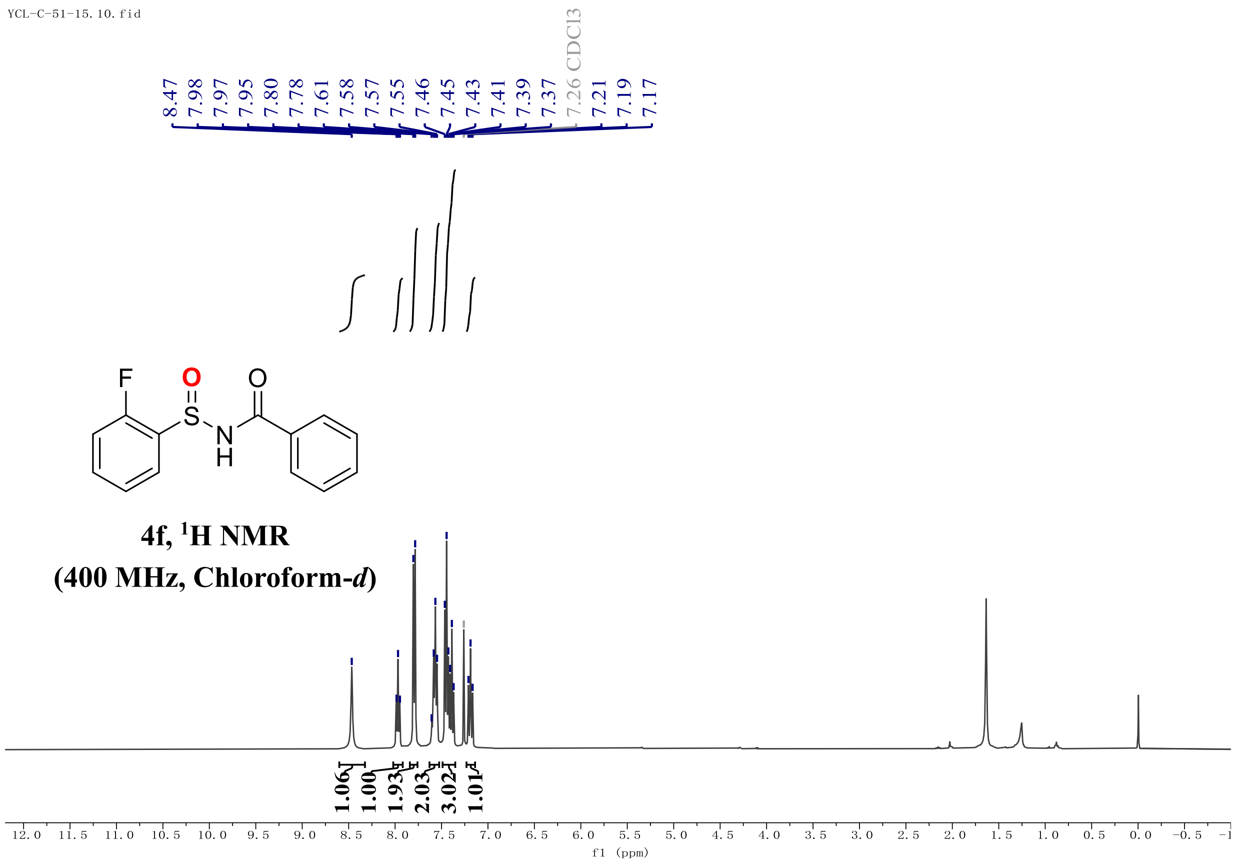
YCL-C-51-15.10.fid
8.47 7.98 7.97 7.95 7.80 7.78 7.61 7.58 7.57 7.55 7.46 7.45 7.43 7.41 7.39 7.37 7.26 CDCl3 7.21 7.19 7.17
F
O
O
S
N
H
4f, ¹H NMR
(400 MHz, Chloroform-d)
1.06 1.00 1.93 2.03 3.02 1.01
12.0 11.5 11.0 10.5 10.0 9.5 9.0 8.5 8.0 7.5 7.0 6.5 6.0 5.5 5.0 4.5 4.0 3.5 3.0 2.5 2.0 1.5 1.0 0.5 0.0 -0.5 -1
f1 (ppm)

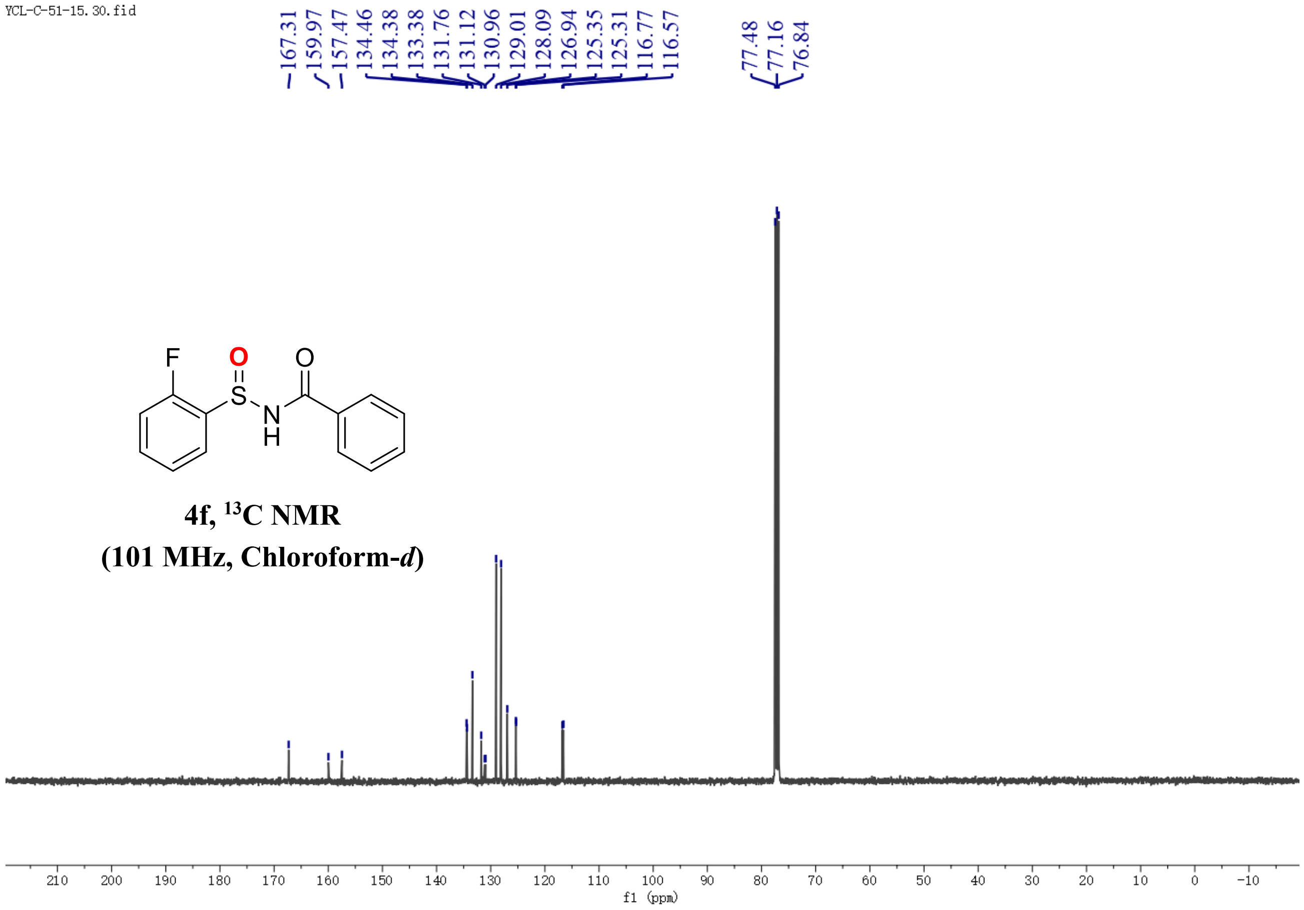
YCL-C-51-15.30.fid
167.31 159.97 157.47 134.46 134.38 133.38 131.76 131.12 130.96 129.01 128.09 126.94 125.35 125.31 116.77 116.57
77.48 77.16 76.84
F
O
O
S
N
H
4f, ¹³C NMR
(101 MHz, Chloroform-d)
210 200 190 180 170 160 150 140 130 120 110 100 90 80 70 60 50 40 30 20 10 0 -10
f1 (ppm)

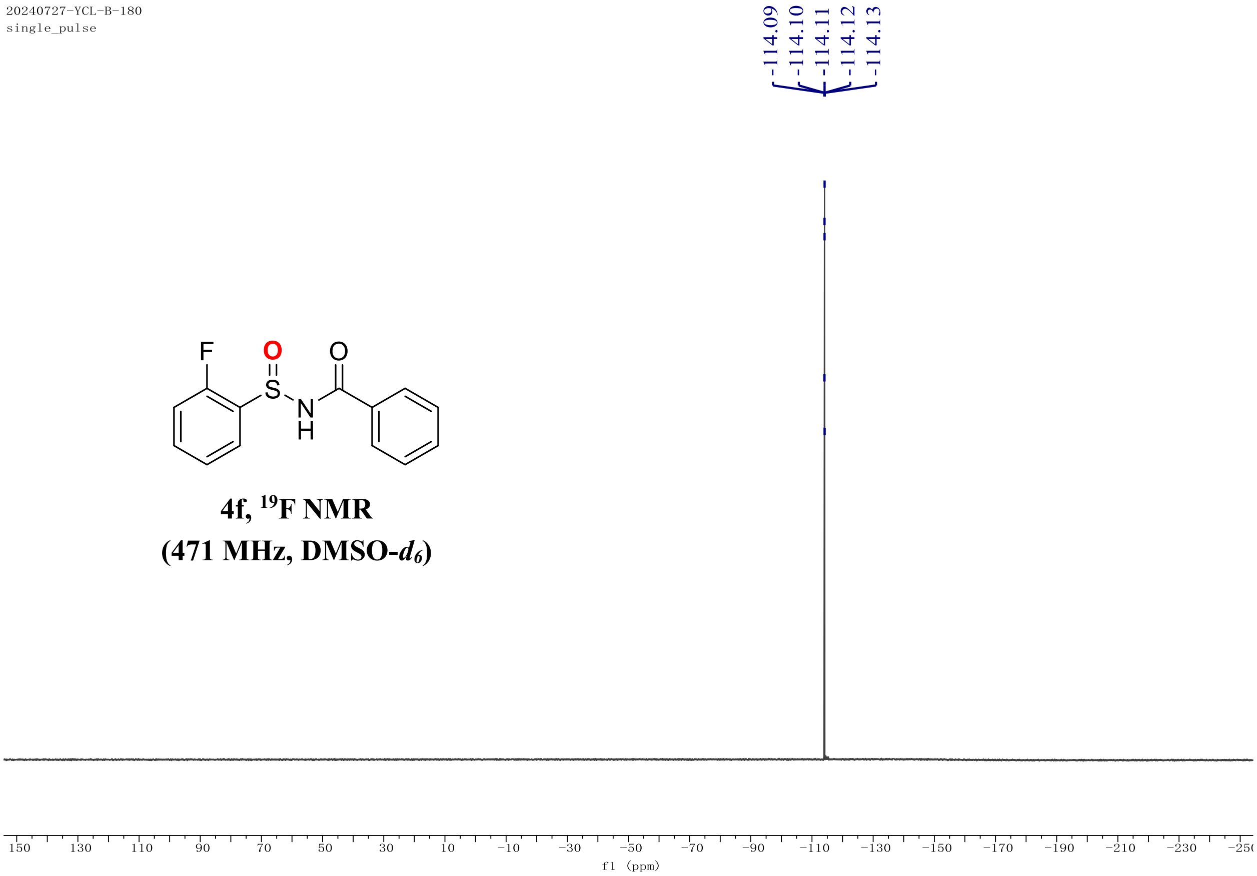

4f, ^{19}F NMR
(471 MHz, DMSO-d_6)

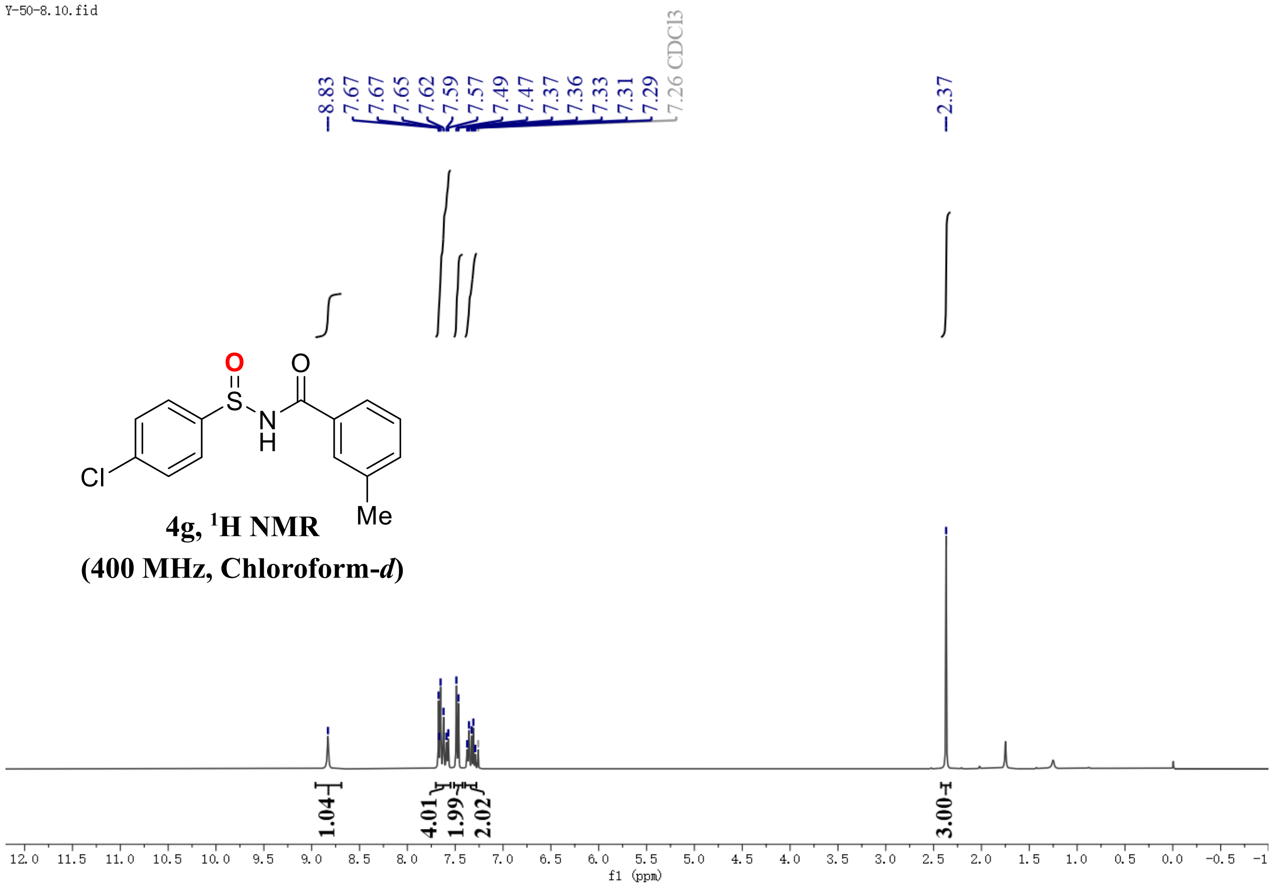

4g, ^{1}H NMR
(400 MHz, Chloroform-d)

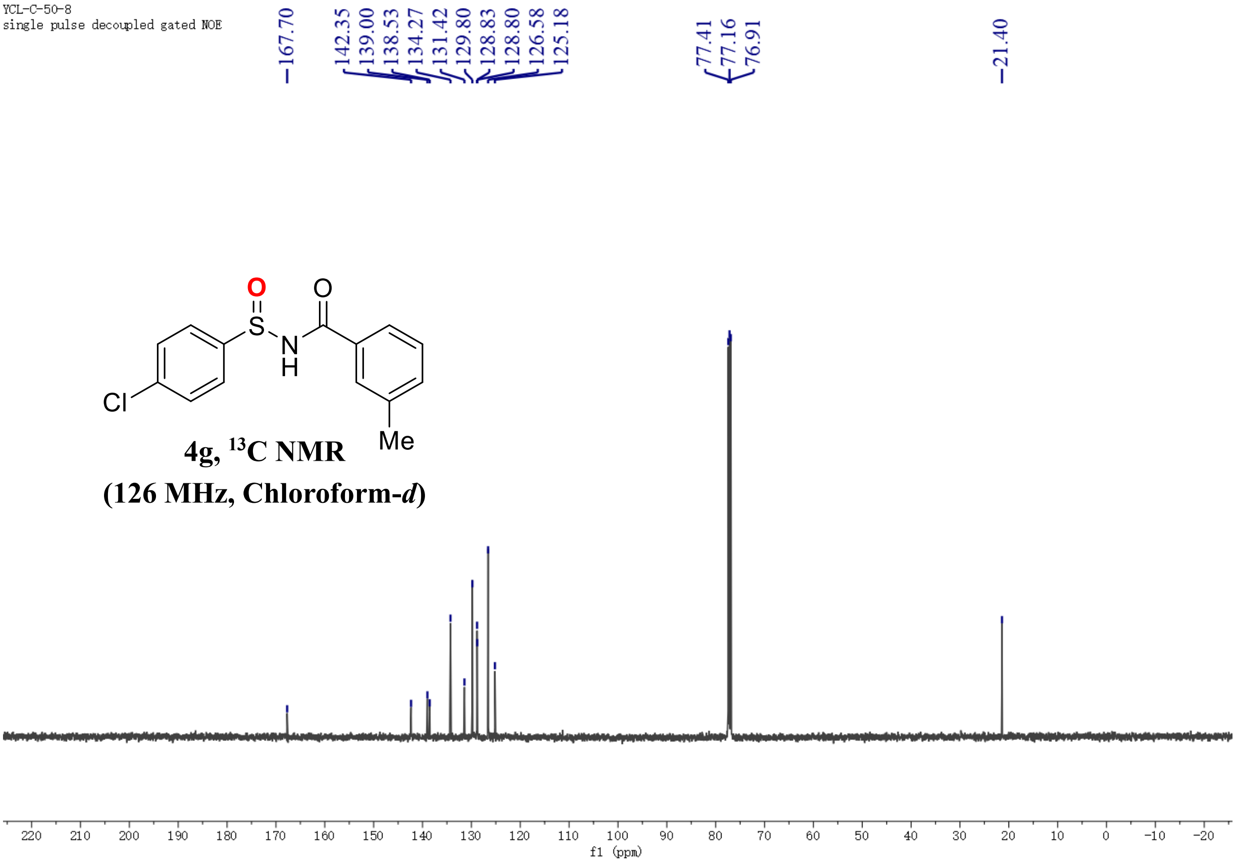
YCL-C-50-8
single pulse decoupled gated NOE
167.70
142.35
139.00
138.53
134.27
131.42
129.80
128.83
128.80
126.58
125.18
77.41
77.16
76.91
21.40
O
S
N
H
O
Cl
Me
4g, 13C NMR
(126 MHz, Chloroform-d)
f1 (ppm)

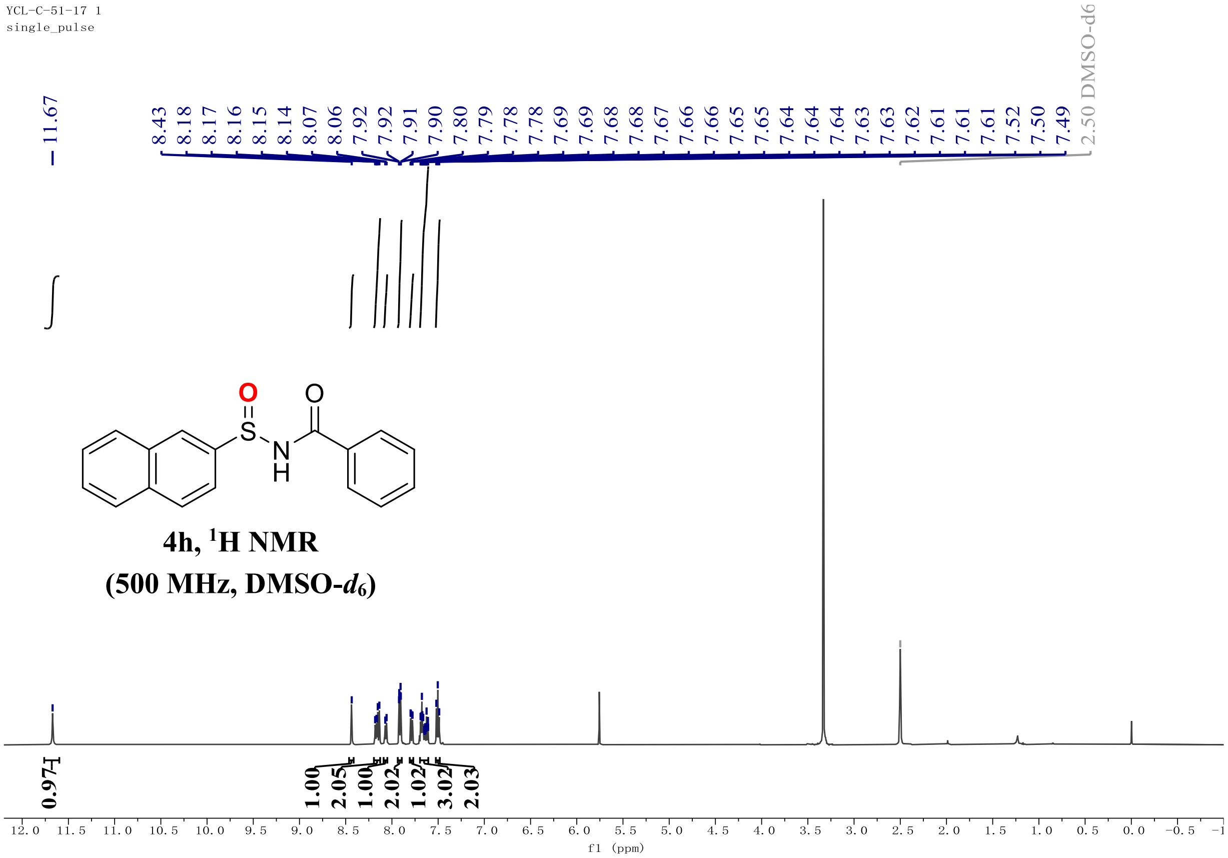
YCL-C-51-17 1
single_pulse
11.67
8.43
8.18
8.17
8.16
8.15
8.14
8.07
8.06
7.92
7.92
7.91
7.90
7.80
7.79
7.78
7.78
7.69
7.69
7.68
7.67
7.66
7.66
7.65
7.65
7.64
7.64
7.63
7.63
7.62
7.61
7.61
7.61
7.52
7.50
7.49
2.50 DMSO-d6
O
S
N
H
O
4h, 1H NMR
(500 MHz, DMSO-d6)
0.97
1.00
2.05
1.00
2.02
1.02
3.02
2.03
f1 (ppm)

YCL-C-51-12.10.fid

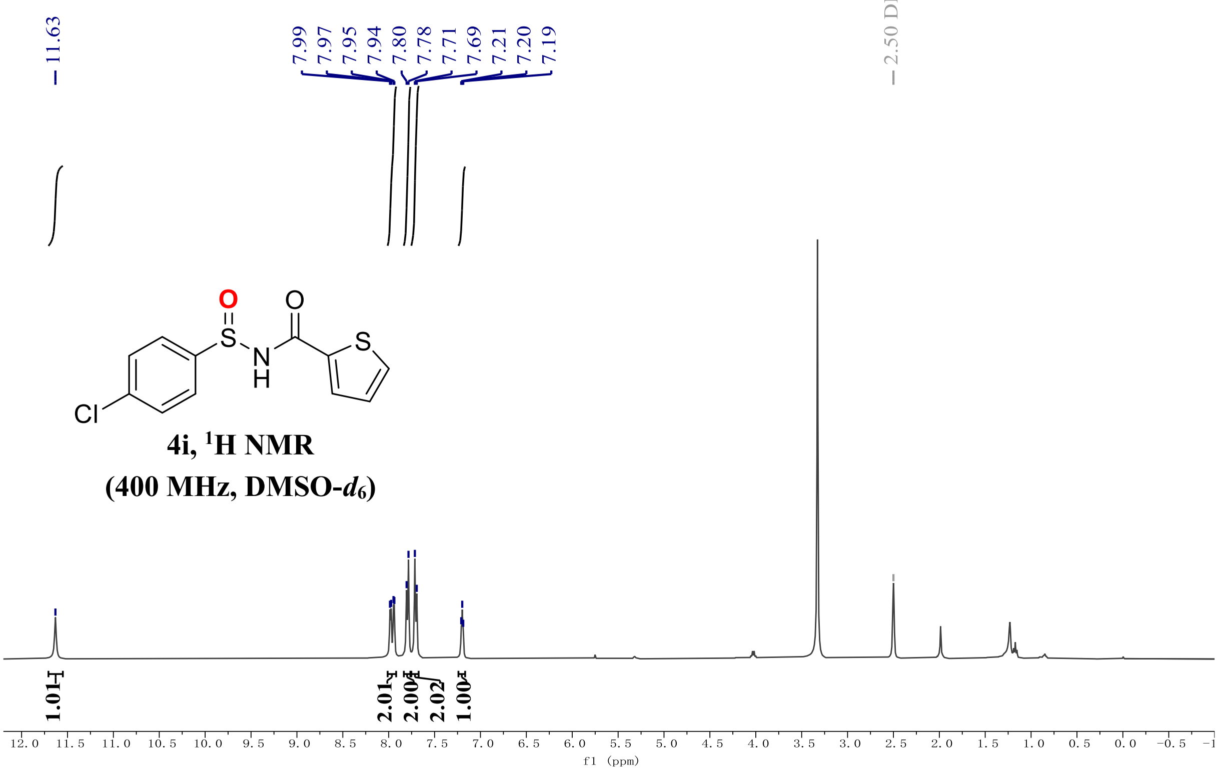

4i, ^{1}H NMR
(400 MHz, DMSO-d_6)

YCL-C-51-12.30.fid

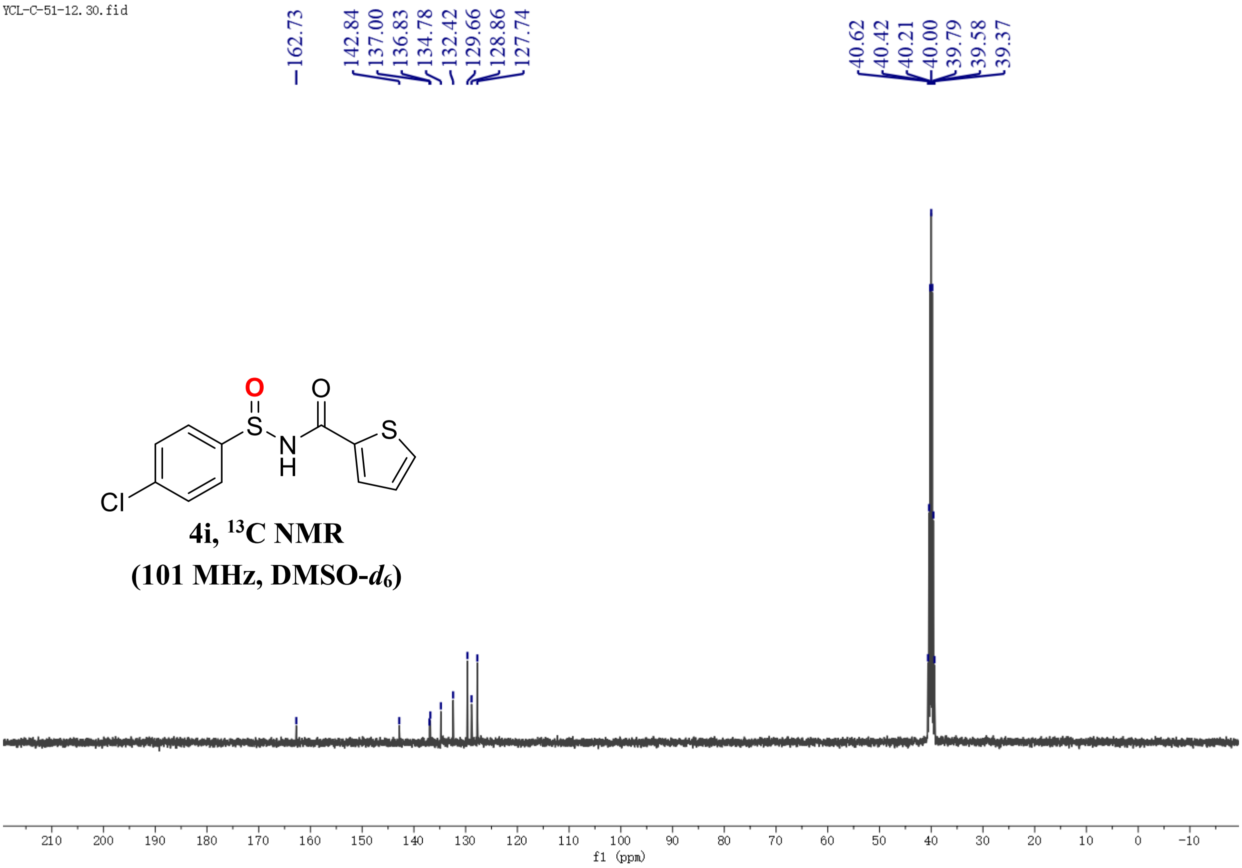

4i, ^{13}C NMR
(101 MHz, DMSO-d_6)

YCL-C-88-1.10.fid

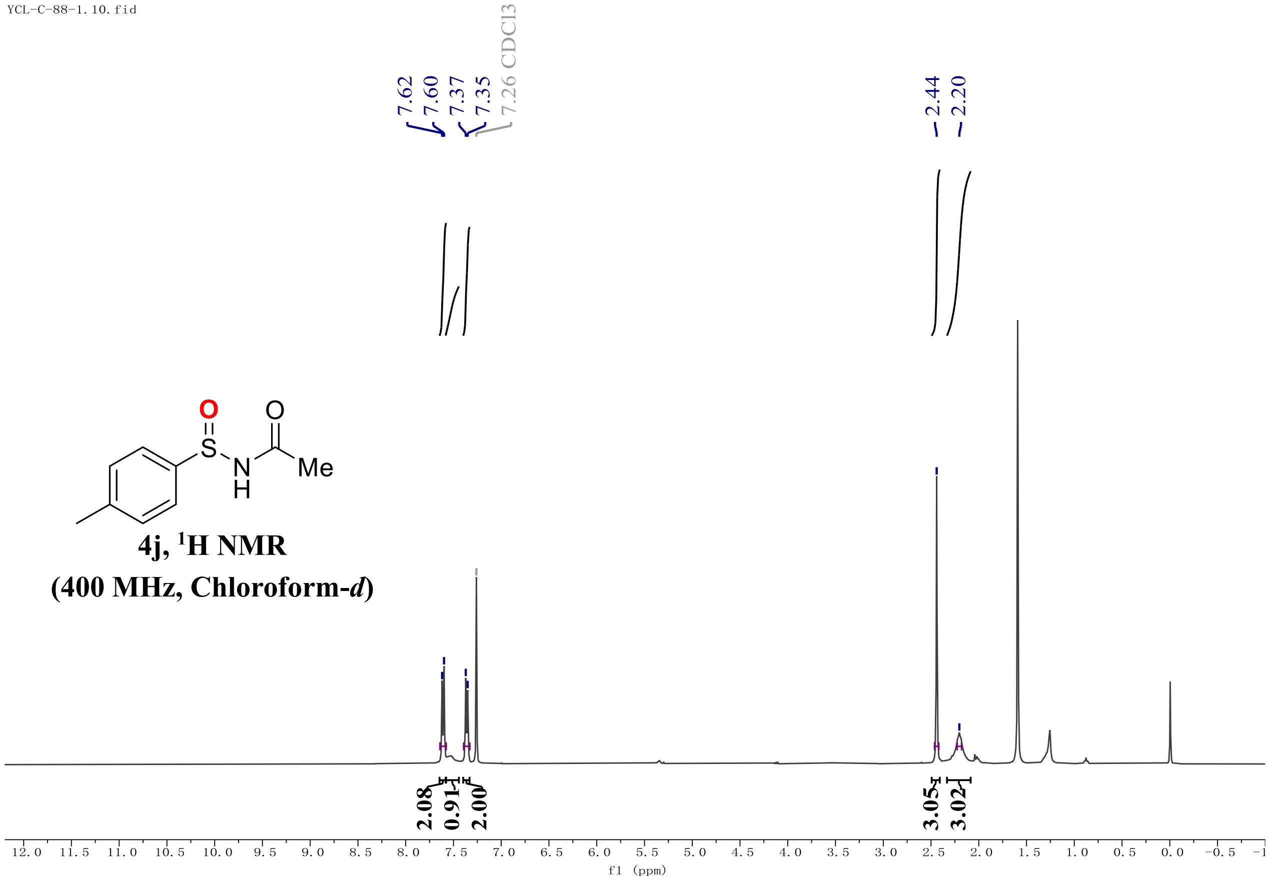

4j, ^{1}H NMR
(400 MHz, Chloroform-*d*)

YCL-C-89-1.10.fid

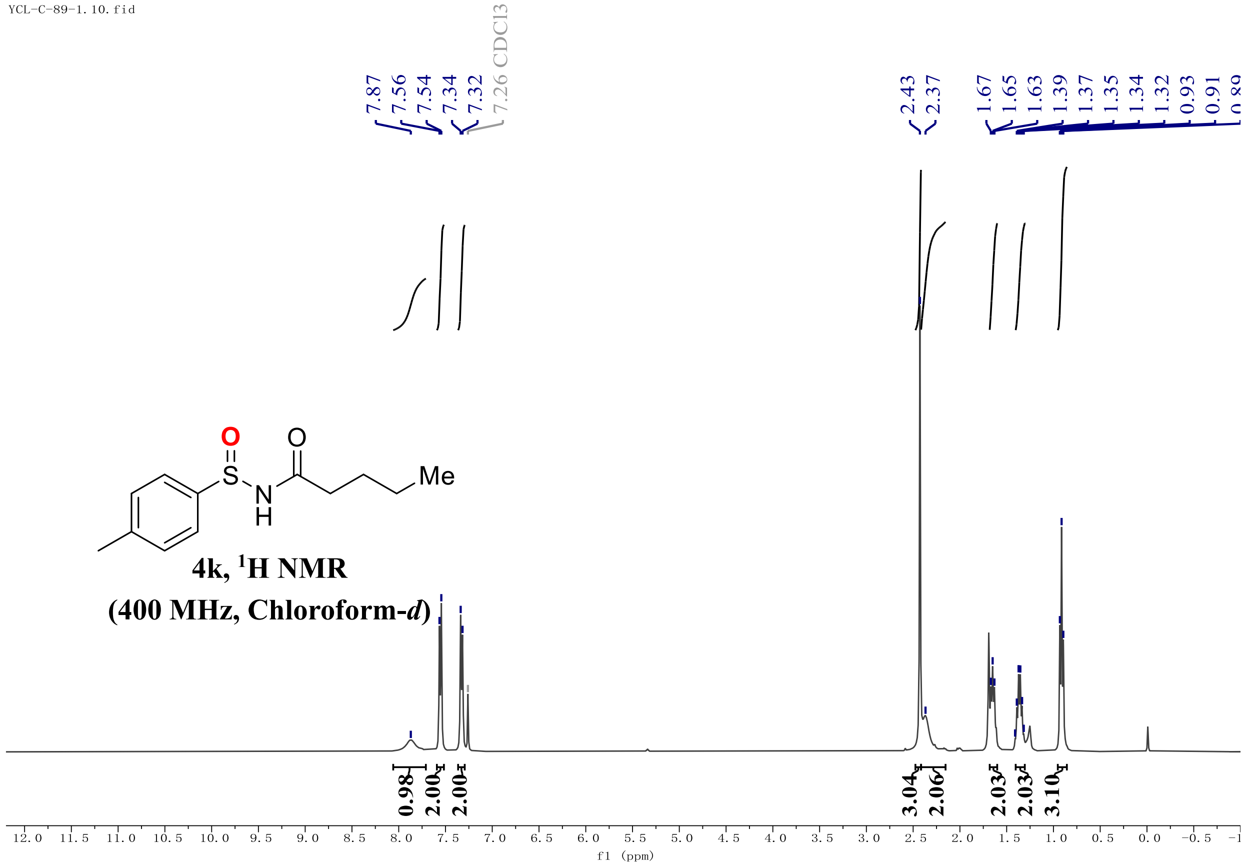

4k, ^{1}H NMR
(400 MHz, Chloroform-*d*)

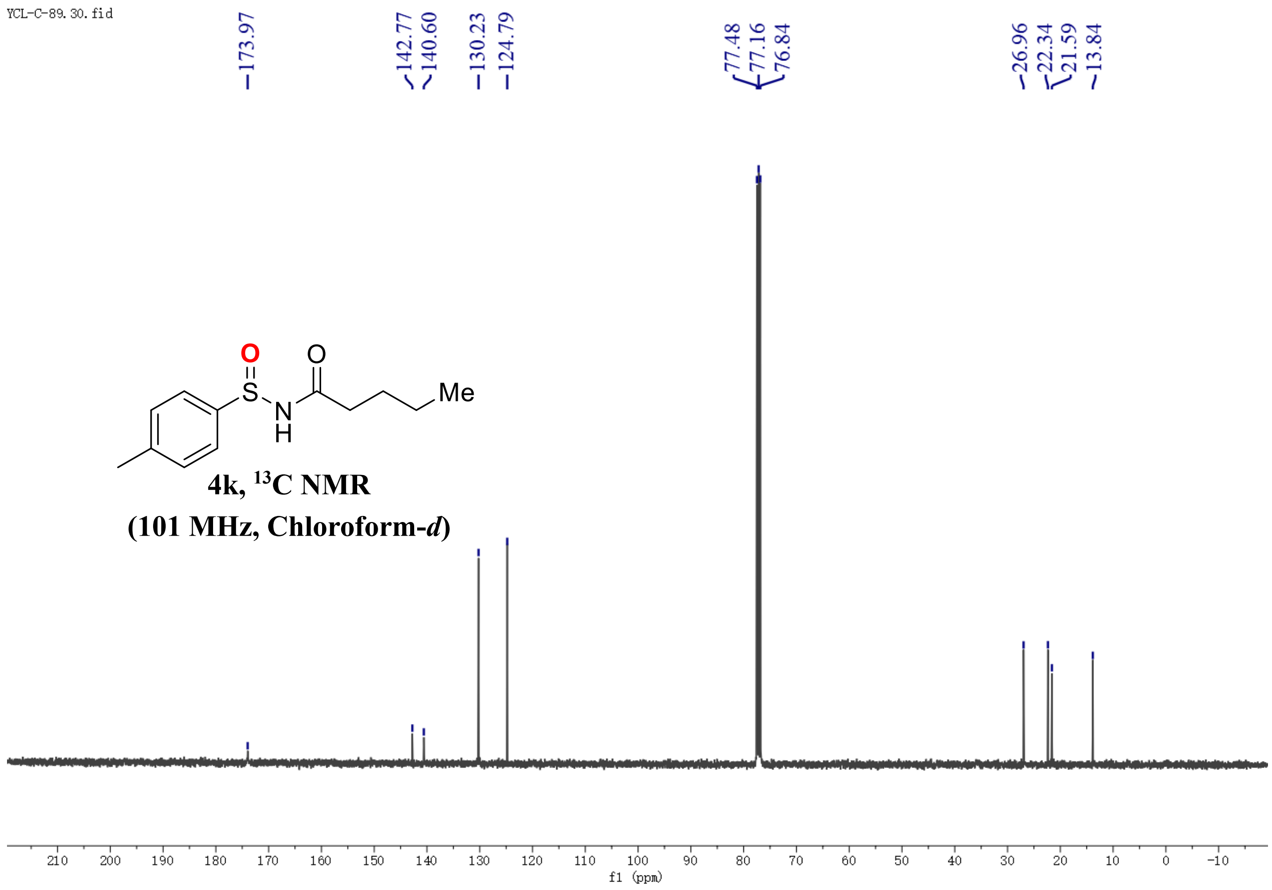
YCL-C-89.30.fid
173.97
142.77
140.60
130.23
124.79
77.48
77.16
76.84
26.96
22.34
21.59
13.84
O
O
S
N
H
Me
4k, ¹³C NMR
(101 MHz, Chloroform-d)
f1 (ppm)

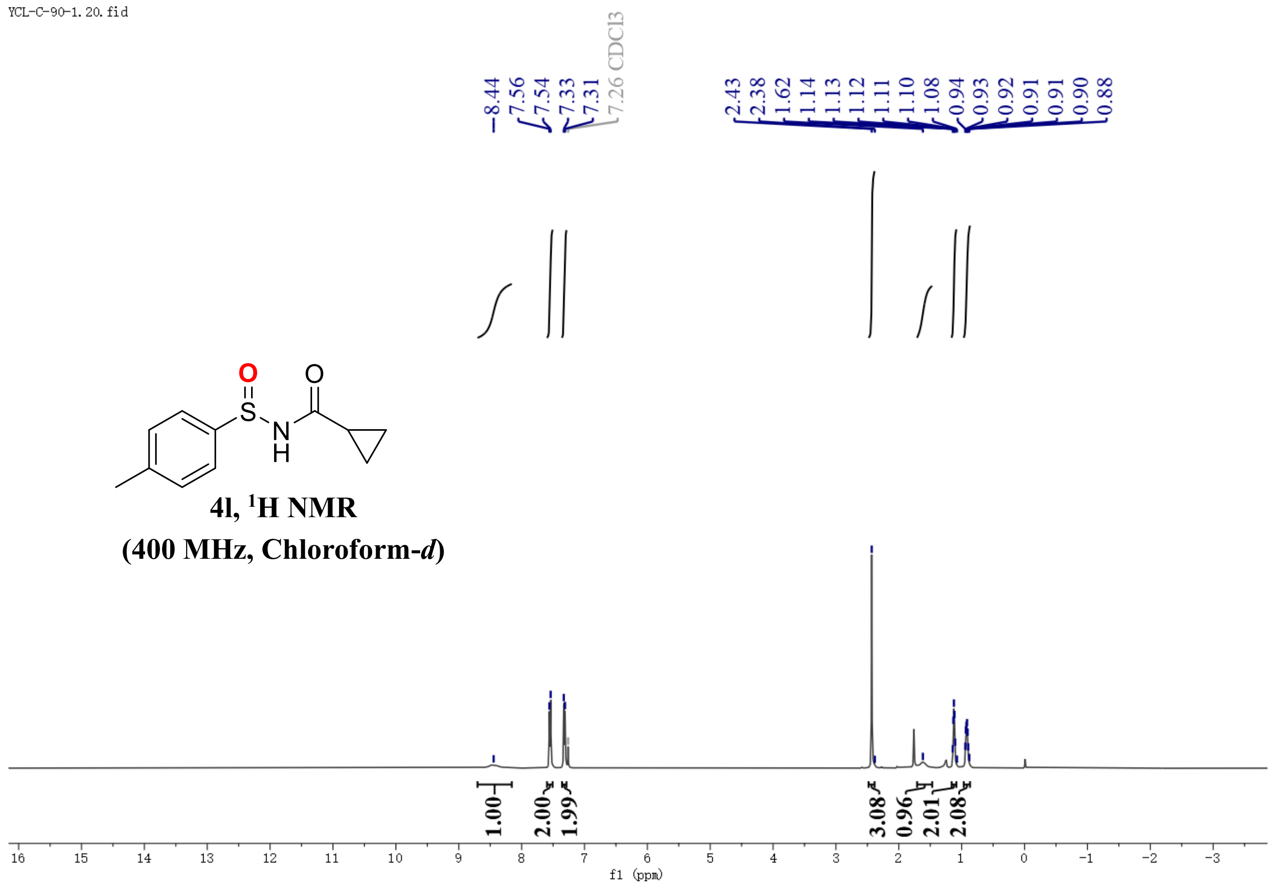
YCL-C-90-1.20.fid
8.44
7.56
7.54
7.33
7.31
7.26 CDCl3
2.43
2.38
1.62
1.14
1.13
1.12
1.11
1.10
1.08
0.94
0.93
0.92
0.91
0.91
0.90
0.88
O
O
S
N
H
4l, ¹H NMR
(400 MHz, Chloroform-d)
1.00
2.00
1.99
3.08
0.96
2.01
2.08
f1 (ppm)

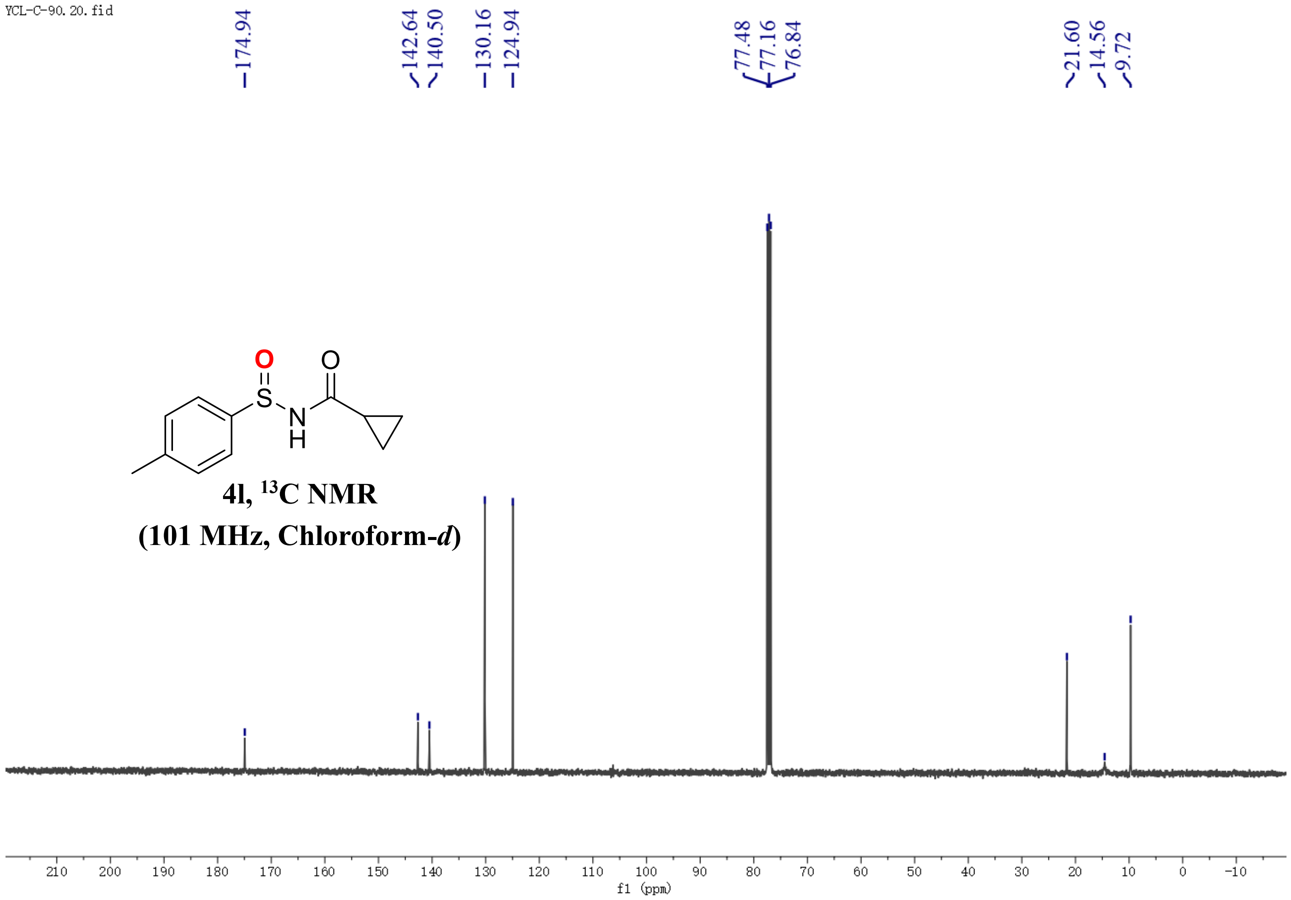

4l, ^{13}C NMR

(101 MHz, Chloroform-*d*)

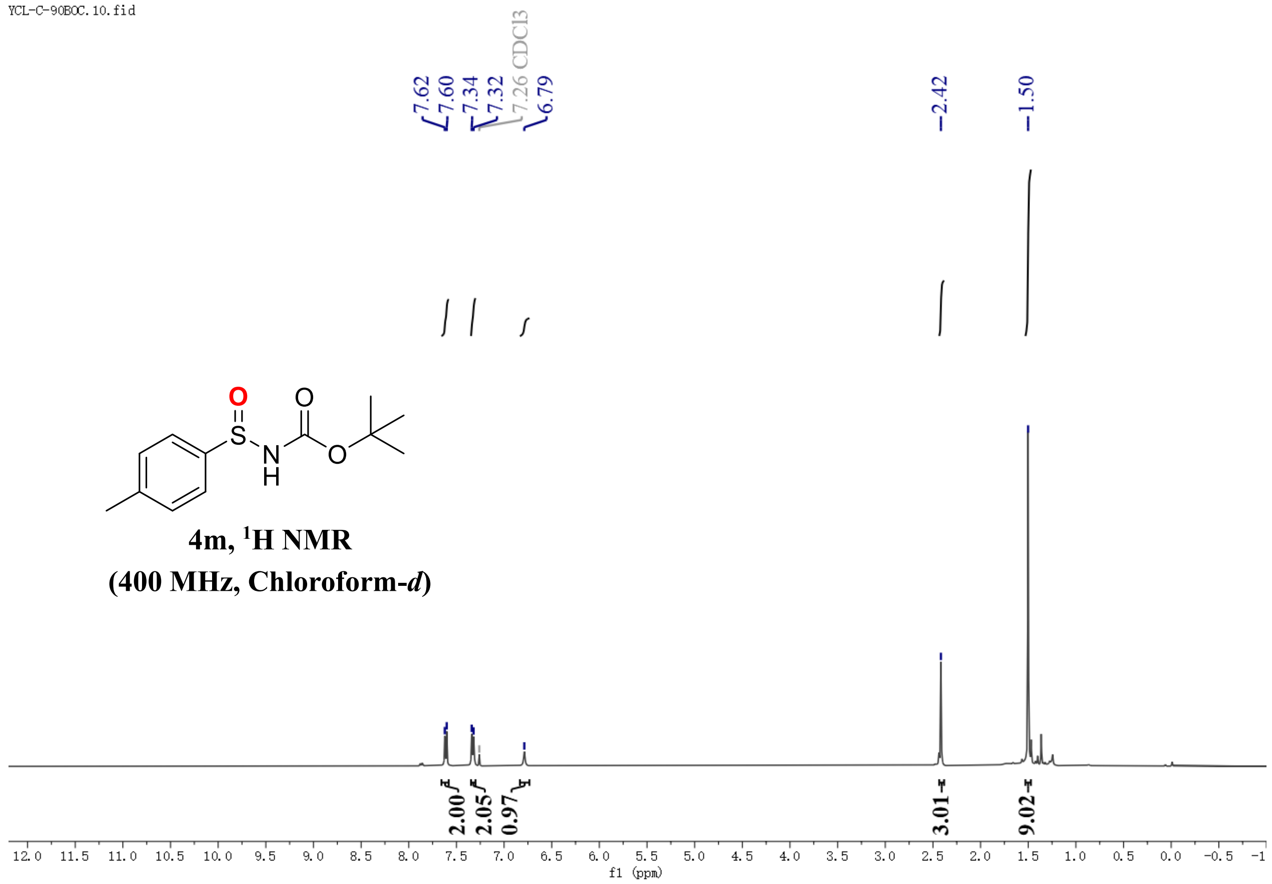

4m, ^{1}H NMR

(400 MHz, Chloroform-*d*)

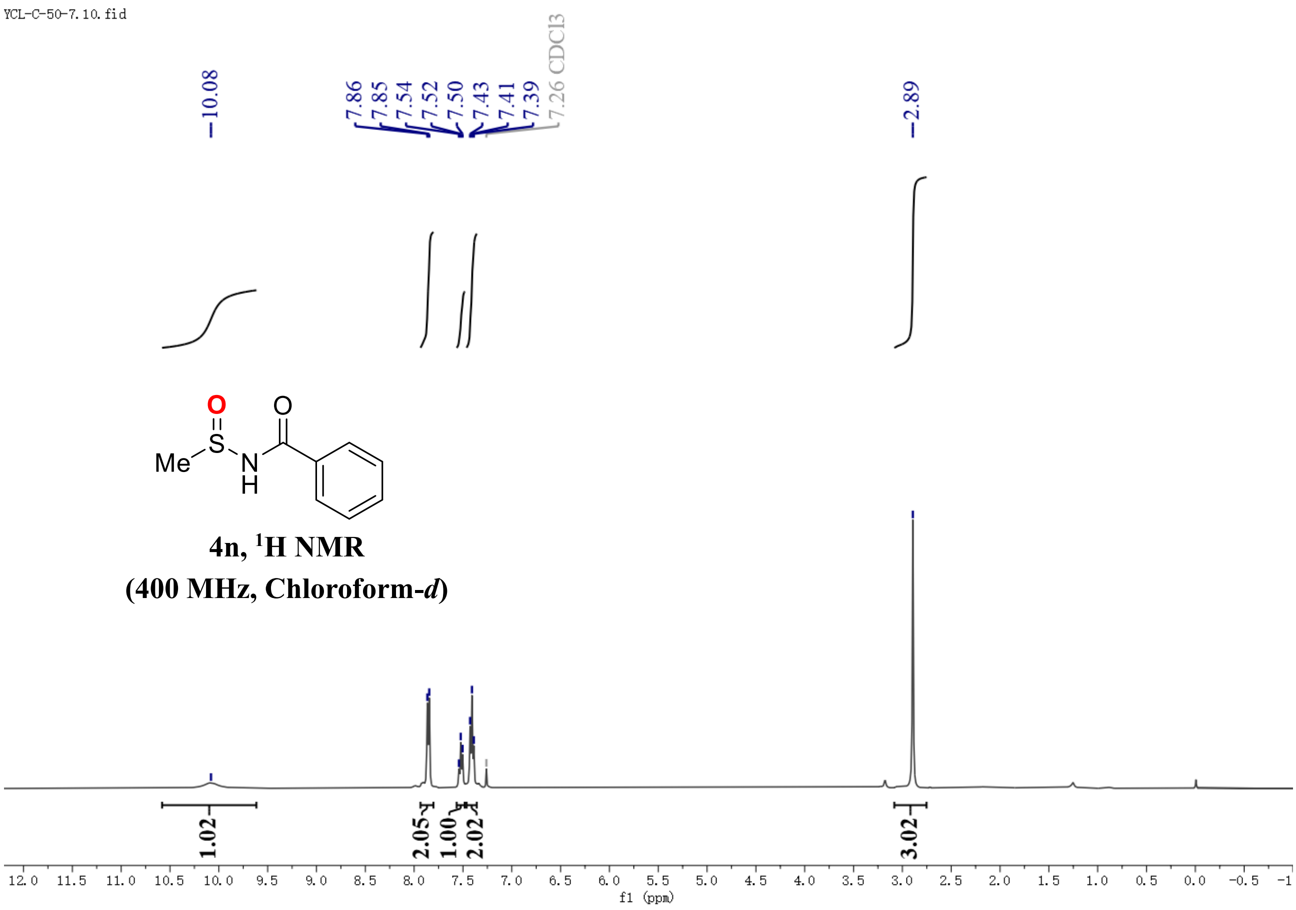
YCL-C-50-7.10.fid
10.08
7.86
7.85
7.54
7.52
7.50
7.43
7.41
7.39
7.26 CDCl3
2.89
Me
S
O
N
H
O
4n, 1H NMR
(400 MHz, Chloroform-d)
1.02
2.05
1.00
2.02
3.02
f1 (ppm)

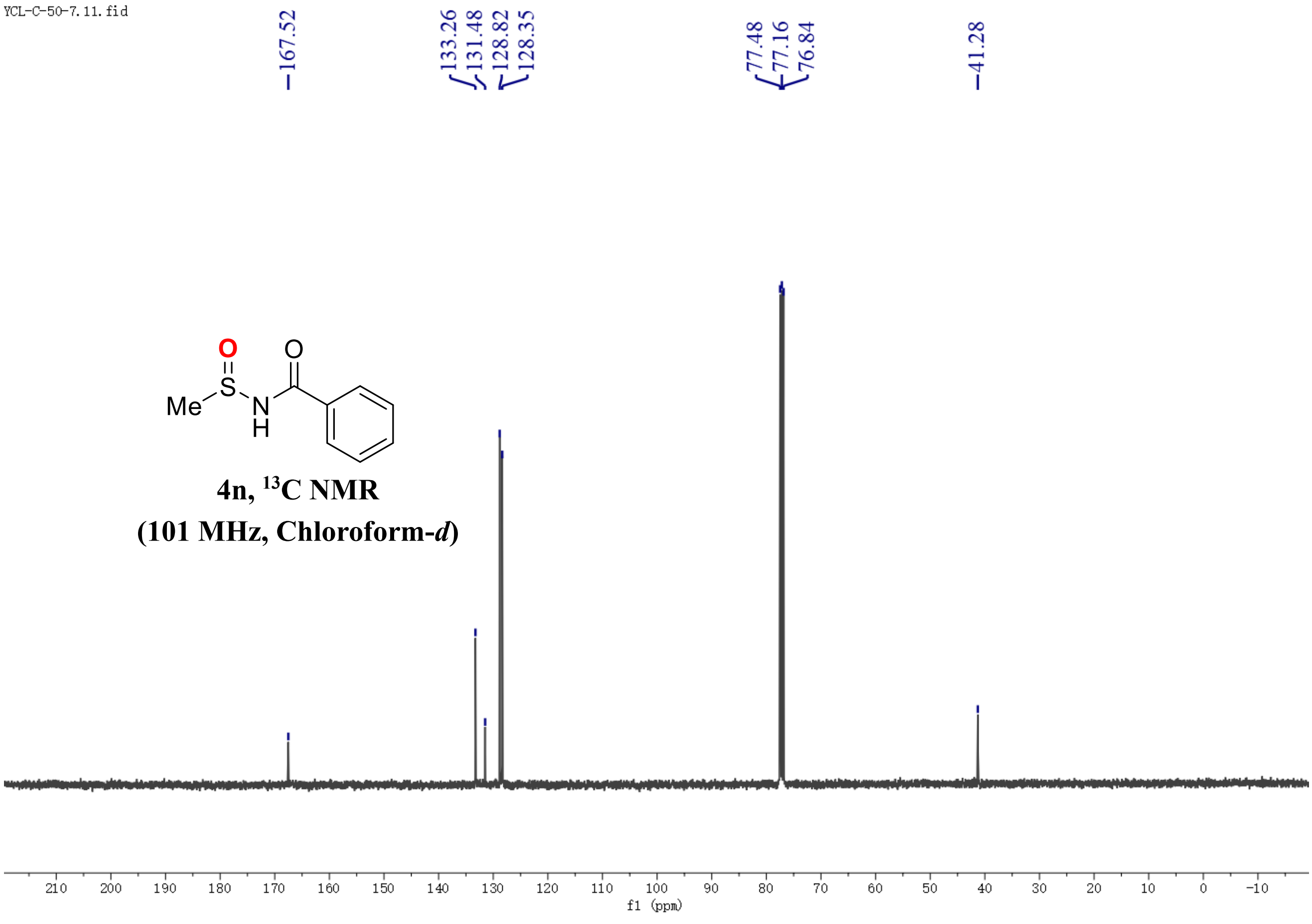
YCL-C-50-7.11.fid
167.52
133.26
131.48
128.82
128.35
77.48
77.16
76.84
41.28
Me
S
O
N
H
O
4n, 13C NMR
(101 MHz, Chloroform-d)
f1 (ppm)

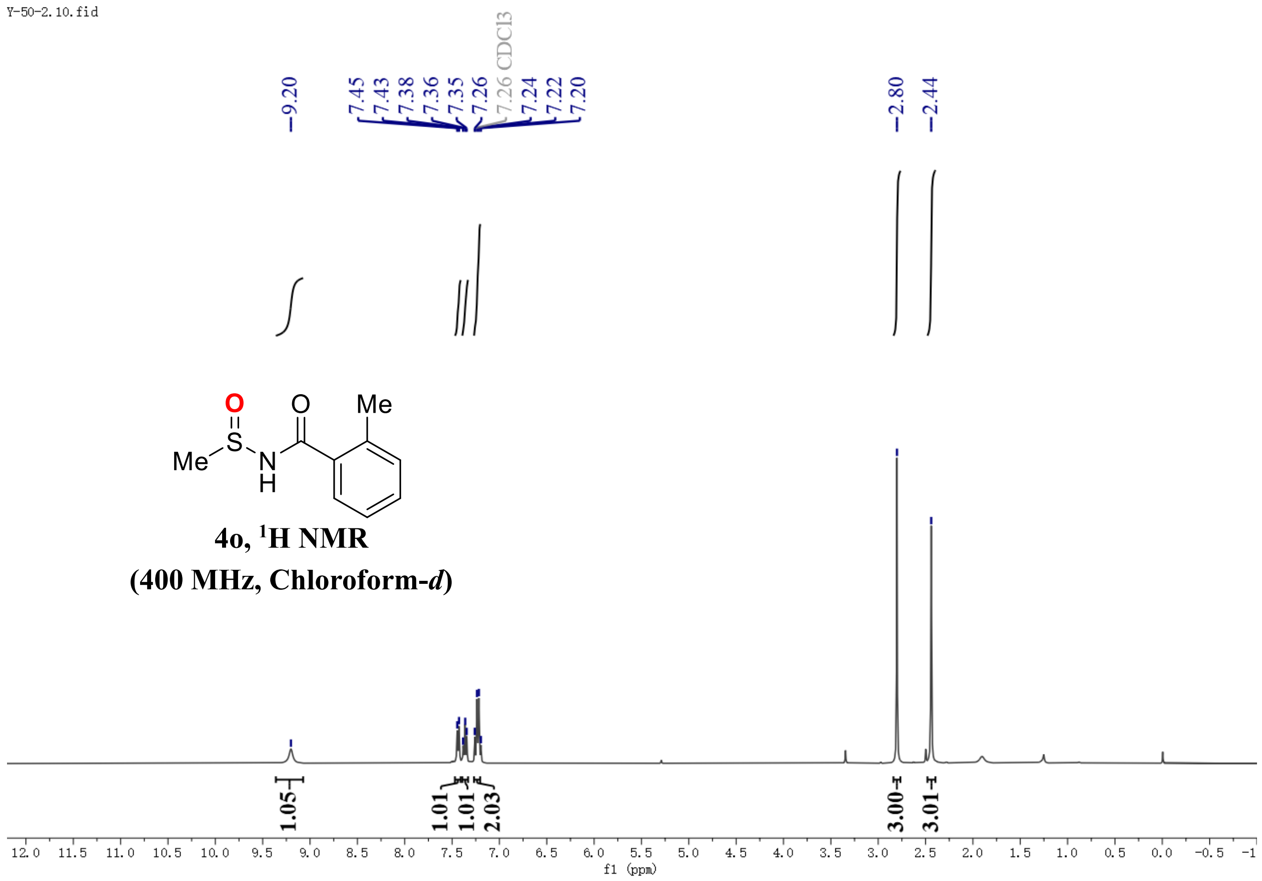
Y-50-2.10.fid
9.20
7.45
7.43
7.38
7.36
7.35
7.26
7.26 CDCl3
7.24
7.22
7.20
2.80
2.44
O
O
Me
Me
S
N
H
4o, 1H NMR
(400 MHz, Chloroform-d)
1.05
1.01
1.01
2.03
3.00
3.01
12.0 11.5 11.0 10.5 10.0 9.5 9.0 8.5 8.0 7.5 7.0 6.5 6.0 5.5 5.0 4.5 4.0 3.5 3.0 2.5 2.0 1.5 1.0 0.5 0.0 -0.5 -1
f1 (ppm)

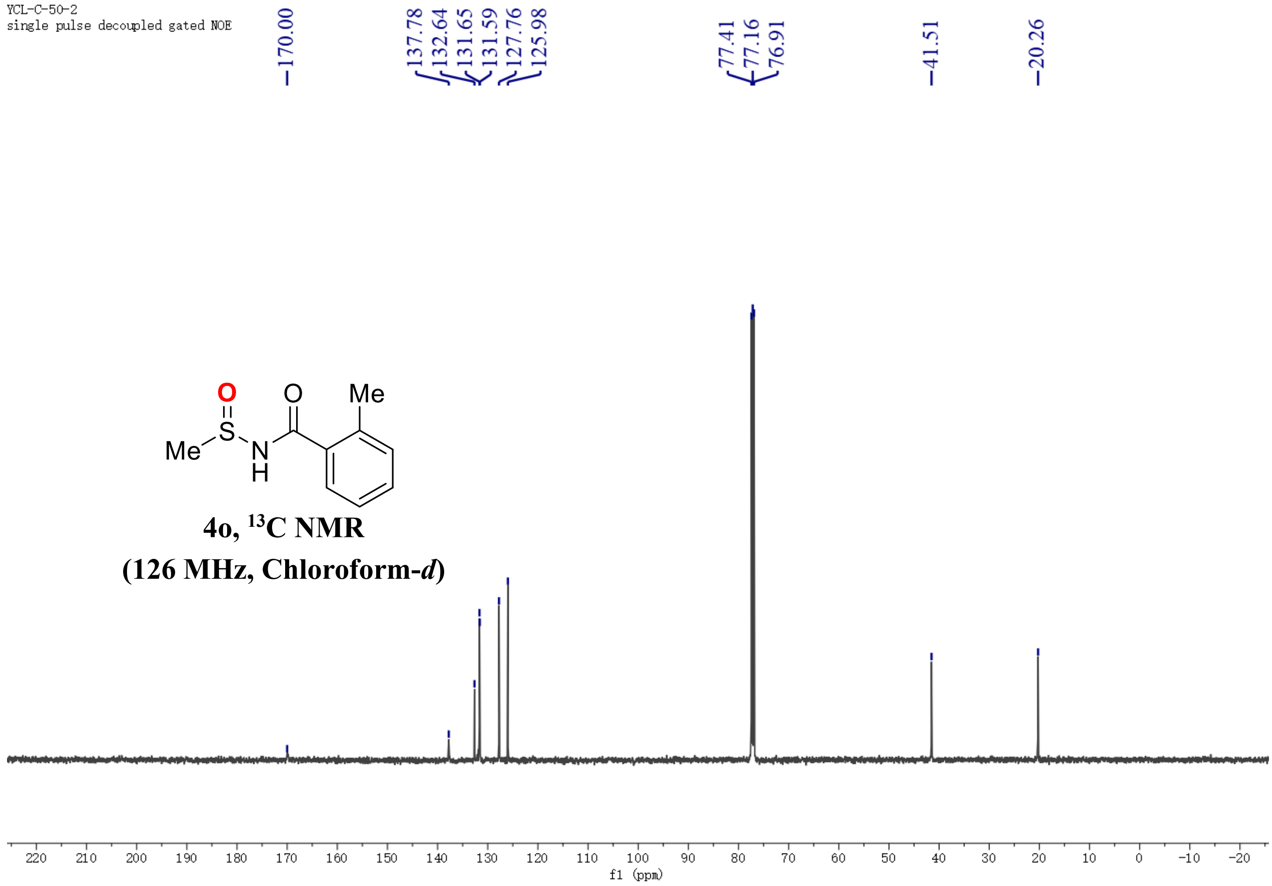
YCL-C-50-2
single pulse decoupled gated NOE
170.00
137.78
132.64
131.65
131.59
127.76
125.98
77.41
77.16
76.91
41.51
20.26
O
O
Me
Me
S
N
H
4o, 13C NMR
(126 MHz, Chloroform-d)
220 210 200 190 180 170 160 150 140 130 120 110 100 90 80 70 60 50 40 30 20 10 0 -10 -20
f1 (ppm)

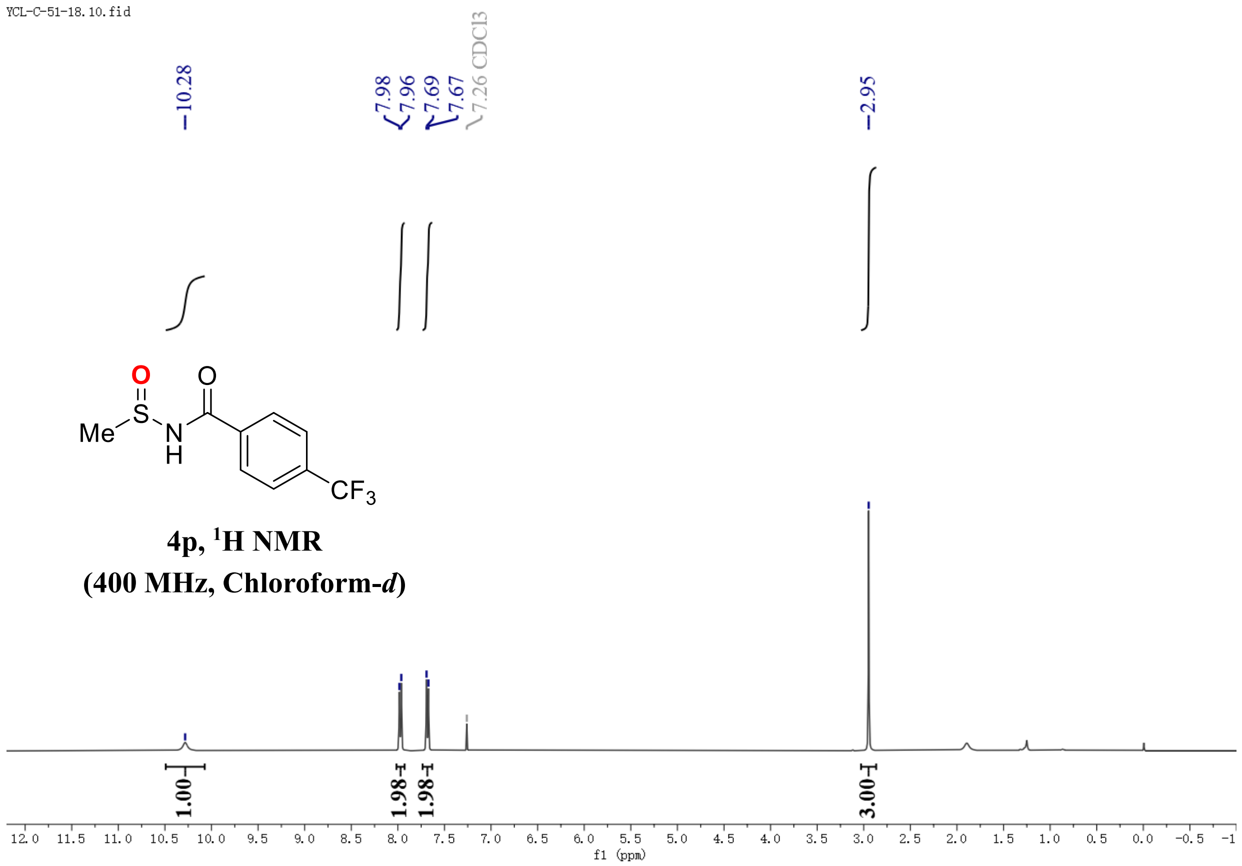

4p, ^{1}H NMR
(400 MHz, Chloroform-*d*)

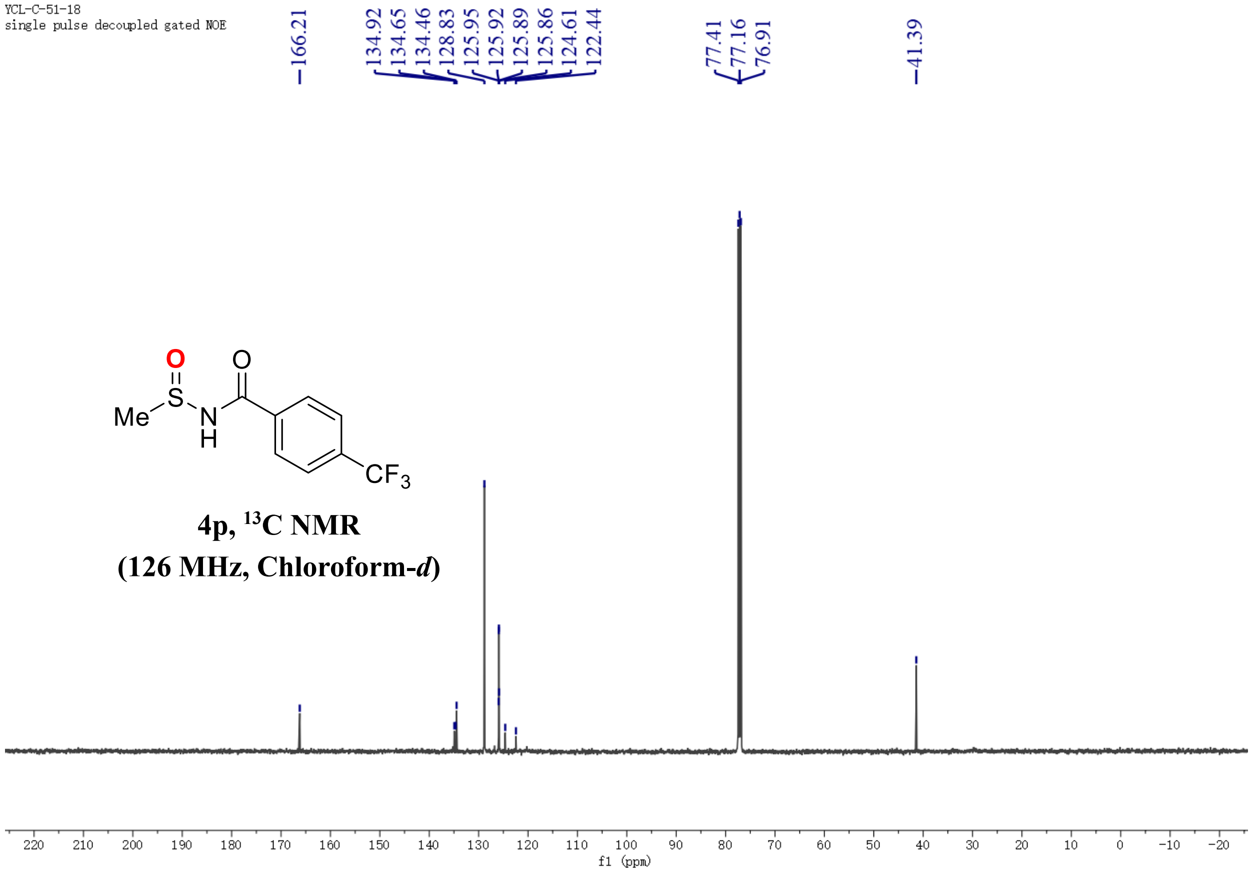

4p, ^{13}C NMR
(126 MHz, Chloroform-*d*)

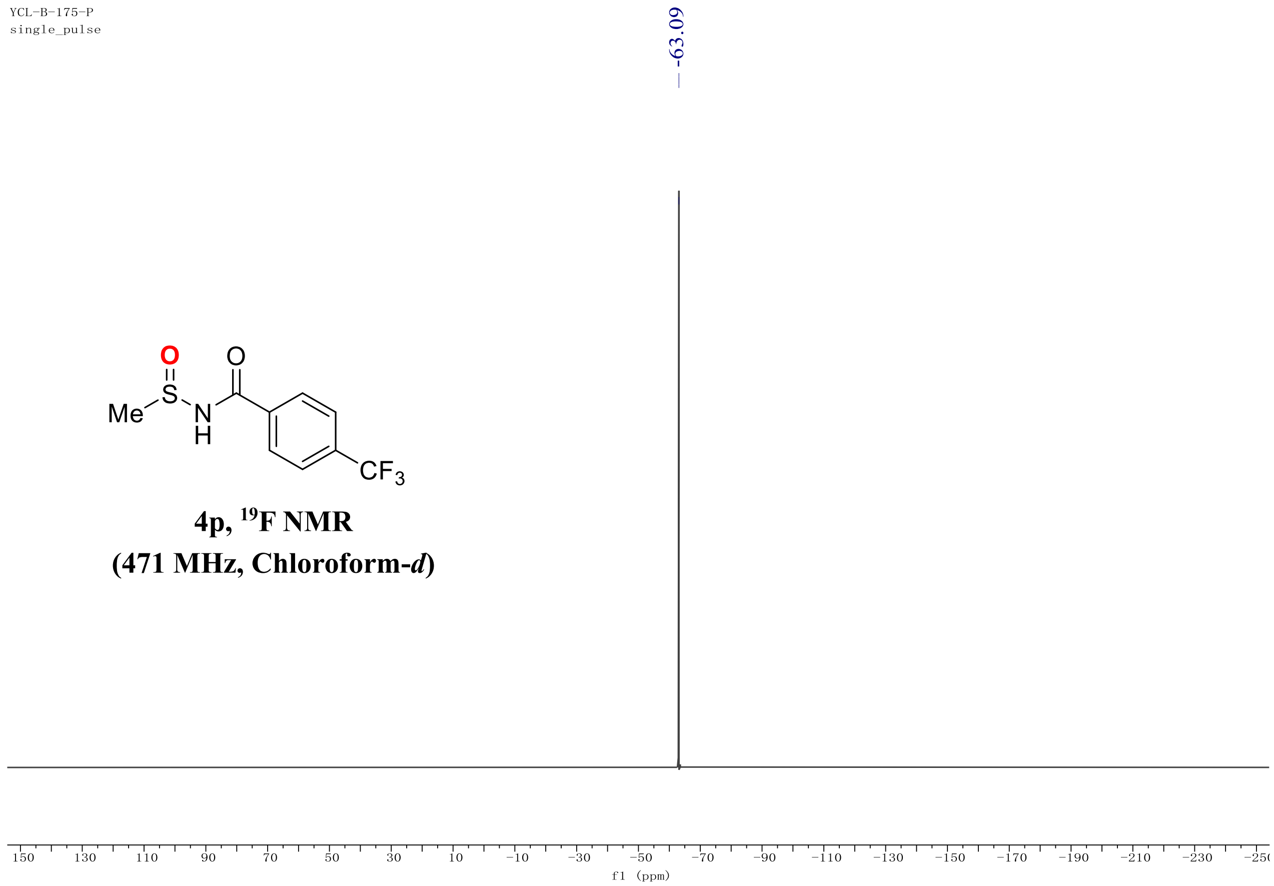
YCL-B-175-P
single_pulse
-63.09
O
O
S
Me
N
H
CF3
4p, 19F NMR
(471 MHz, Chloroform-d)
f1 (ppm)

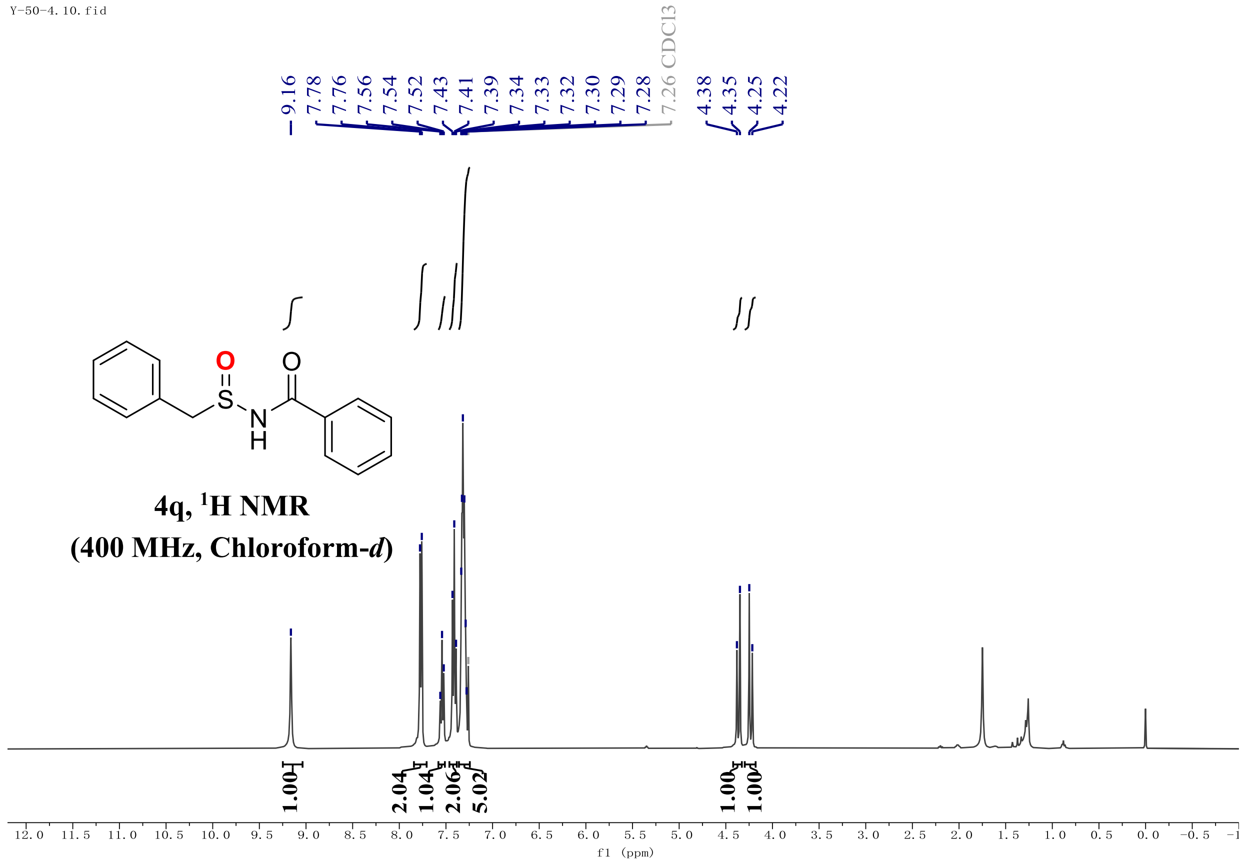
Y-50-4.10.fid
9.16
7.78
7.76
7.56
7.54
7.52
7.43
7.41
7.39
7.34
7.33
7.32
7.30
7.29
7.28
7.26 CDCl3
4.38
4.35
4.25
4.22
O
O
S
N
H
4q, 1H NMR
(400 MHz, Chloroform-d)
1.00
2.04
1.04
2.06
5.02
1.00
1.00
f1 (ppm)

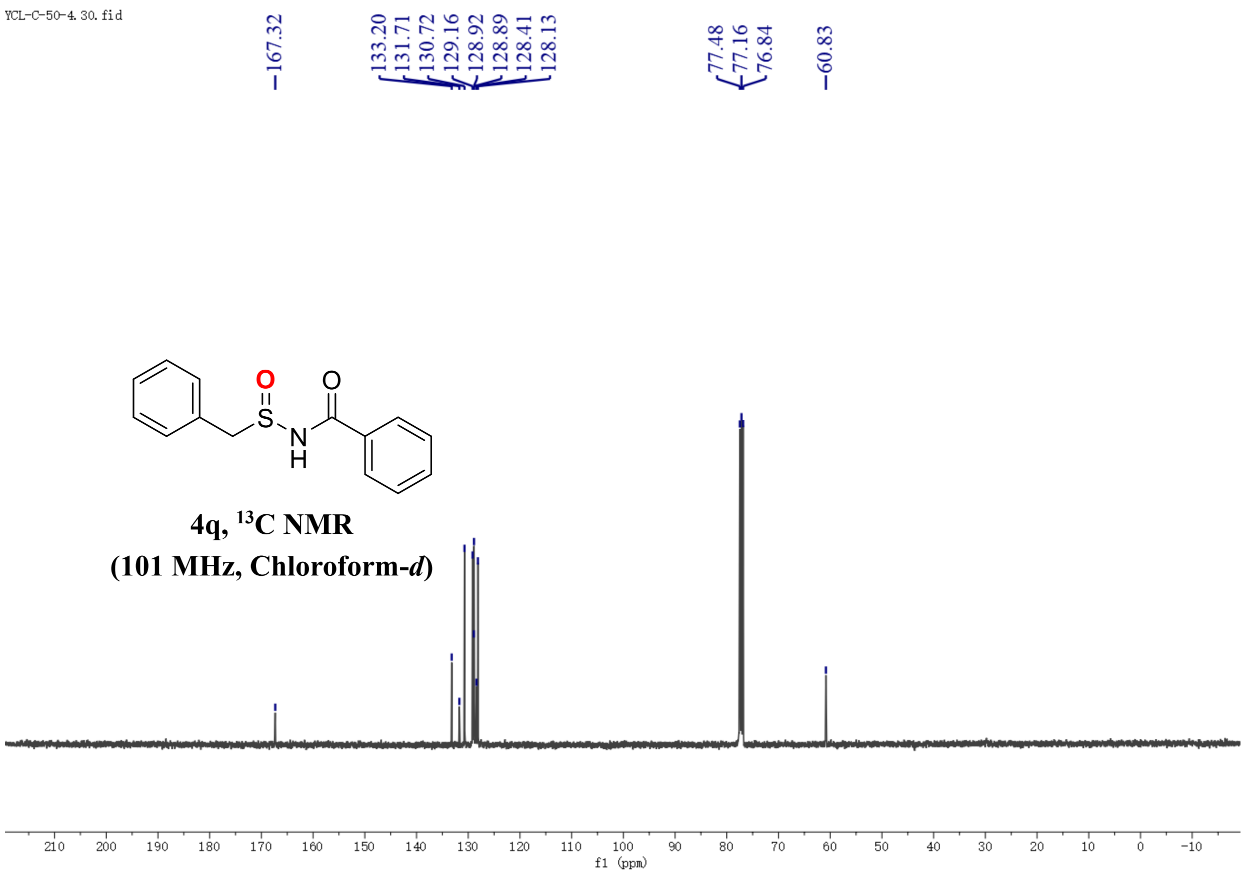
YCL-C-50-4.30.fid
167.32
133.20
131.71
130.72
129.16
128.92
128.89
128.41
128.13
77.48
77.16
76.84
60.83
4q, 13C NMR
(101 MHz, Chloroform-d)
f1 (ppm)

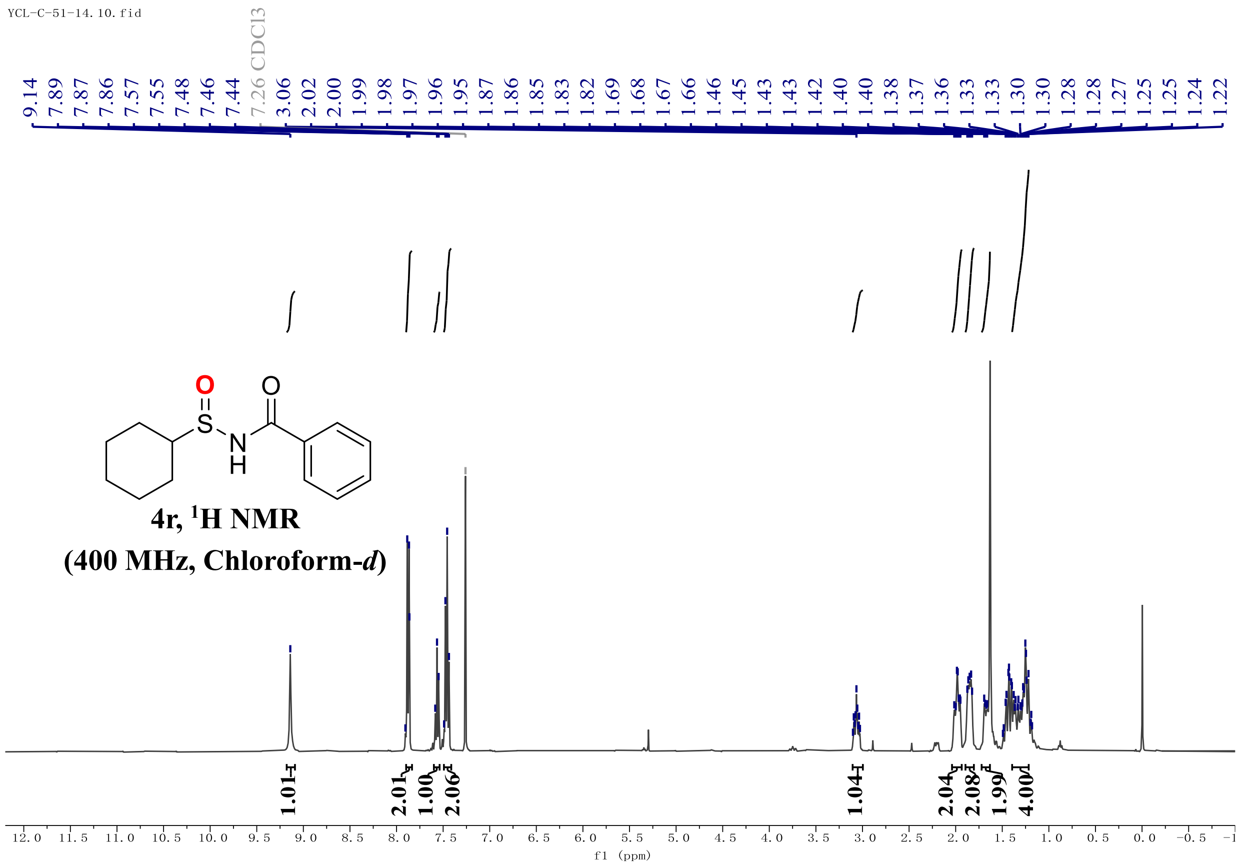
YCL-C-51-14.10.fid
9.14 7.89 7.87 7.86 7.57 7.55 7.48 7.46 7.44 7.26 CDCl3 3.06 2.02 2.00 1.99 1.98 1.97 1.96 1.95 1.87 1.86 1.85 1.83 1.82 1.69 1.68 1.67 1.66 1.46 1.45 1.43 1.43 1.42 1.40 1.40 1.38 1.37 1.36 1.33 1.33 1.30 1.30 1.28 1.28 1.27 1.25 1.25 1.24 1.22
4r, 1H NMR
(400 MHz, Chloroform-d)
1.01
2.01
1.00
2.06
1.04
2.04
2.08
1.99
4.00
f1 (ppm)

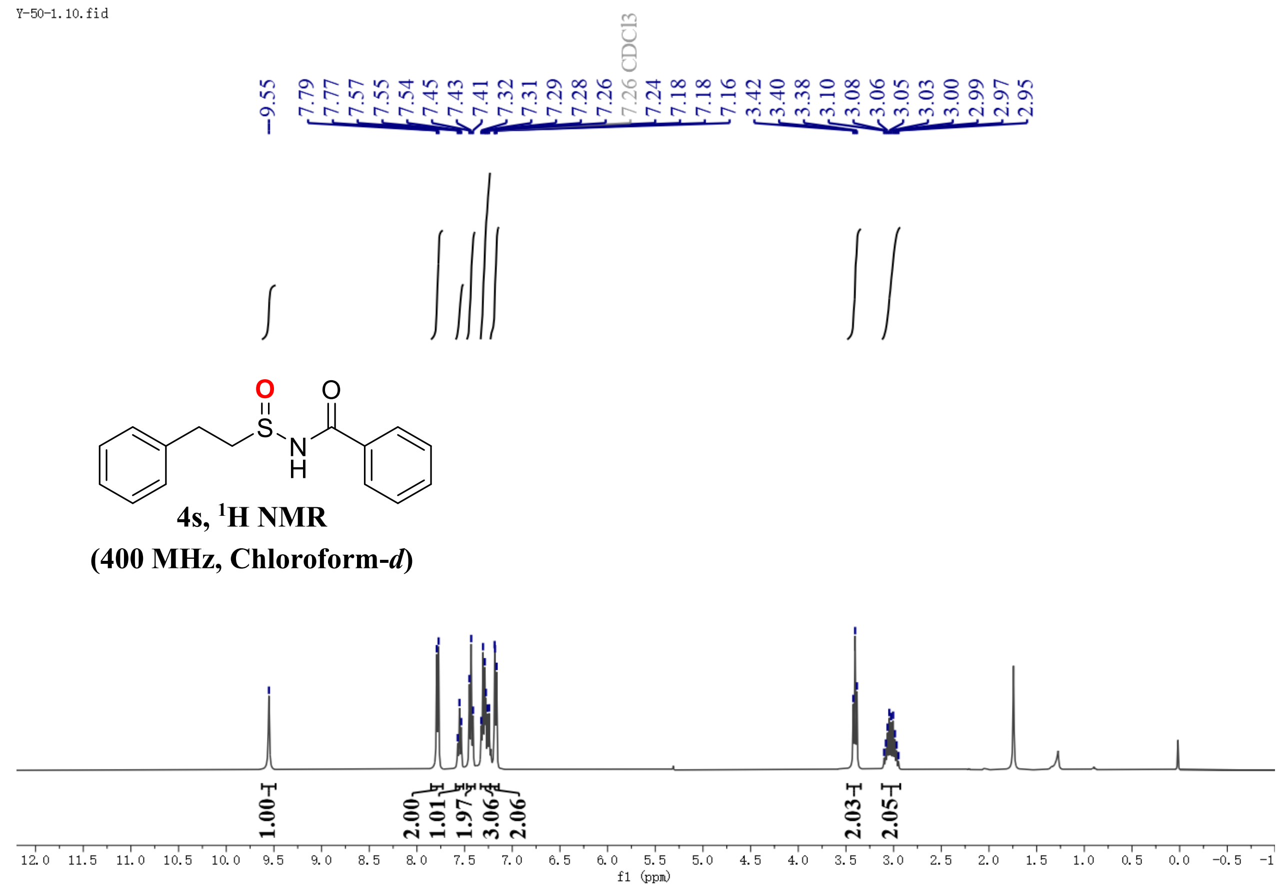

4s, ^{1}H NMR

(400 MHz, Chloroform-*d*)

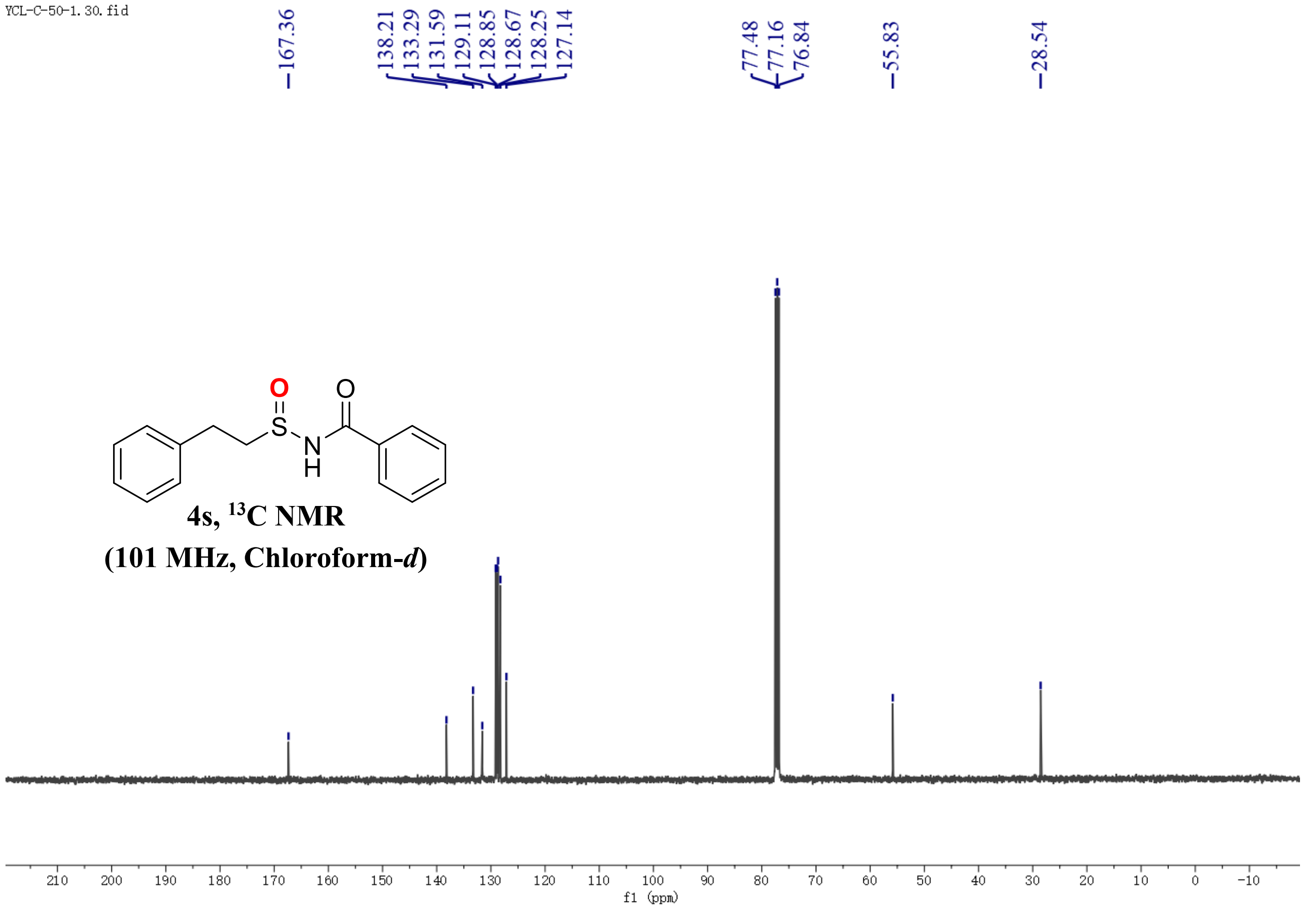

4s, ^{13}C NMR

(101 MHz, Chloroform-*d*)

YCL-B-142
single_pulse

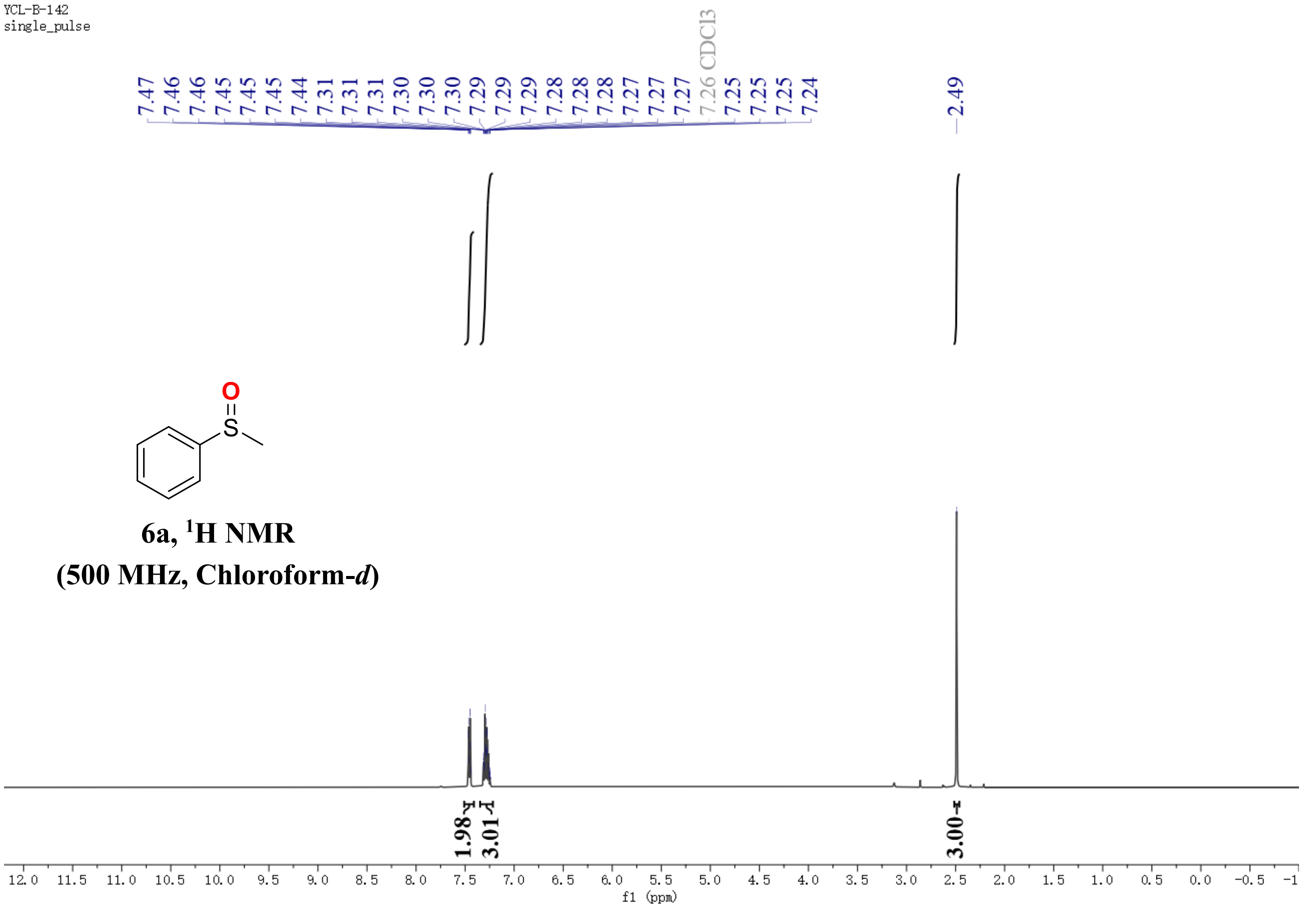

6a, ^{1}H NMR
(500 MHz, Chloroform-*d*)

YCL-B-150-P
single_pulse

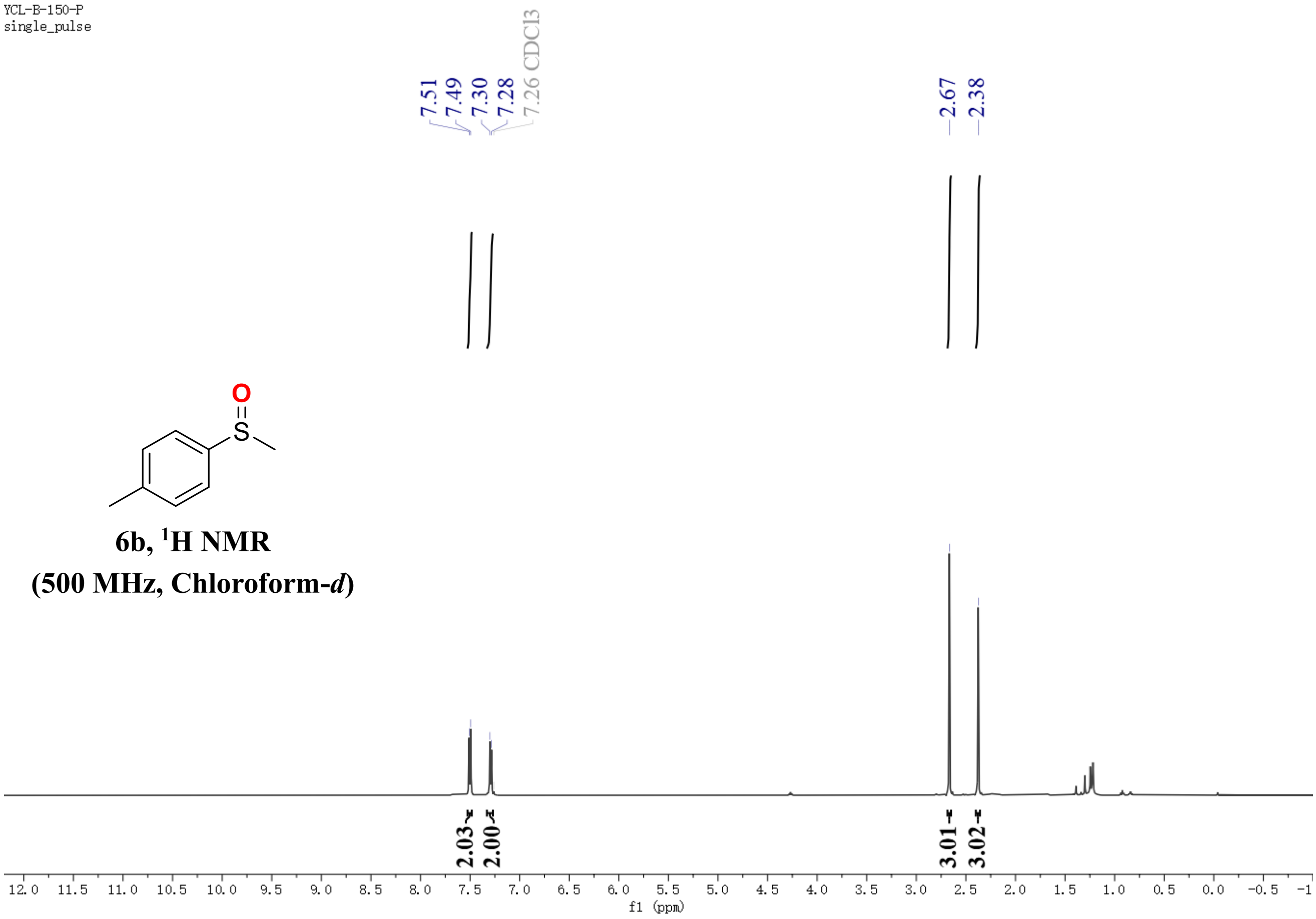

6b, ^{1}H NMR
(500 MHz, Chloroform-*d*)

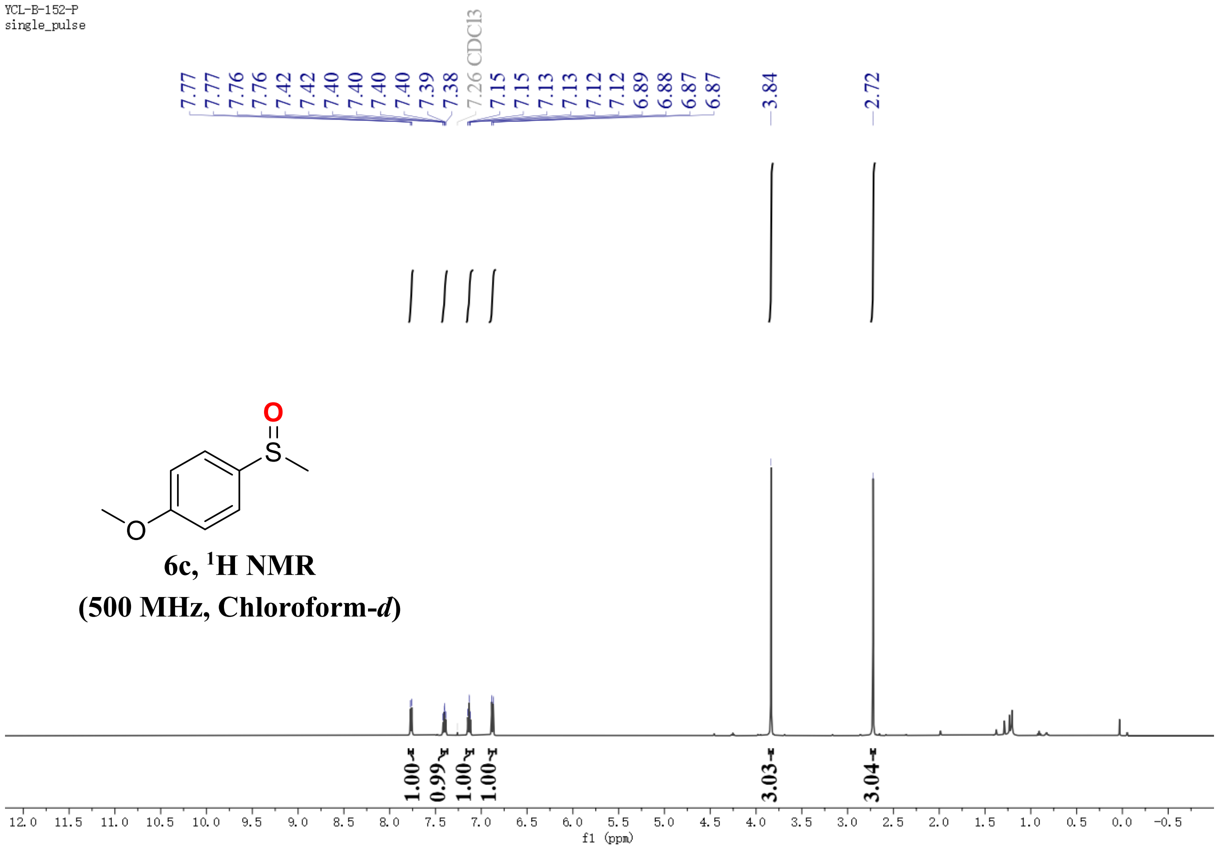
YCL-B-152-P
single_pulse
7.77
7.77
7.76
7.76
7.42
7.42
7.40
7.40
7.40
7.40
7.39
7.38
7.26 CDCl3
7.15
7.15
7.13
7.13
7.12
7.12
6.89
6.88
6.87
6.87
3.84
2.72
O
S
O
6c, 1H NMR
(500 MHz, Chloroform-d)
1.00
0.99
1.00
1.00
3.03
3.04
f1 (ppm)

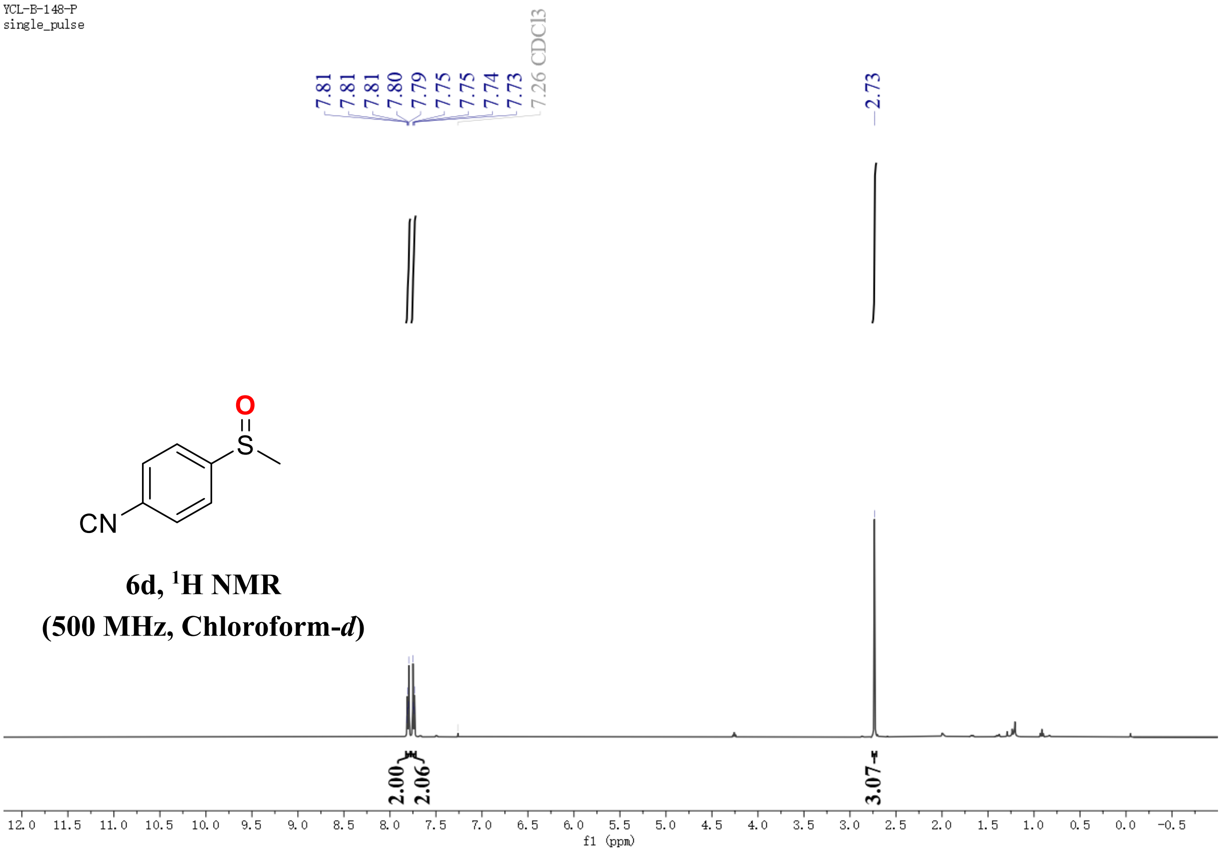
YCL-B-148-P
single_pulse
7.81
7.81
7.81
7.80
7.79
7.75
7.75
7.74
7.73
7.26 CDCl3
2.73
O
S
CN
6d, 1H NMR
(500 MHz, Chloroform-d)
2.00
2.06
3.07
f1 (ppm)

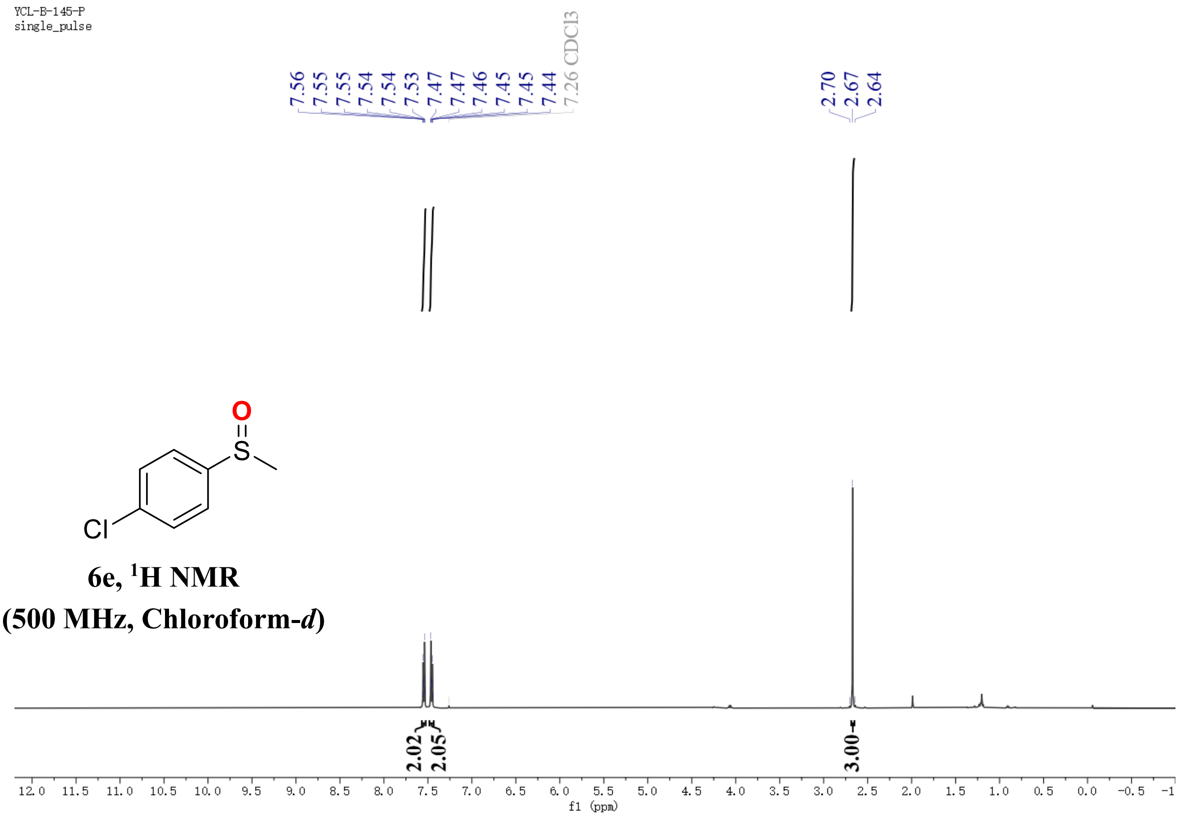

6e, ^{1}H NMR
(500 MHz, Chloroform-*d*)

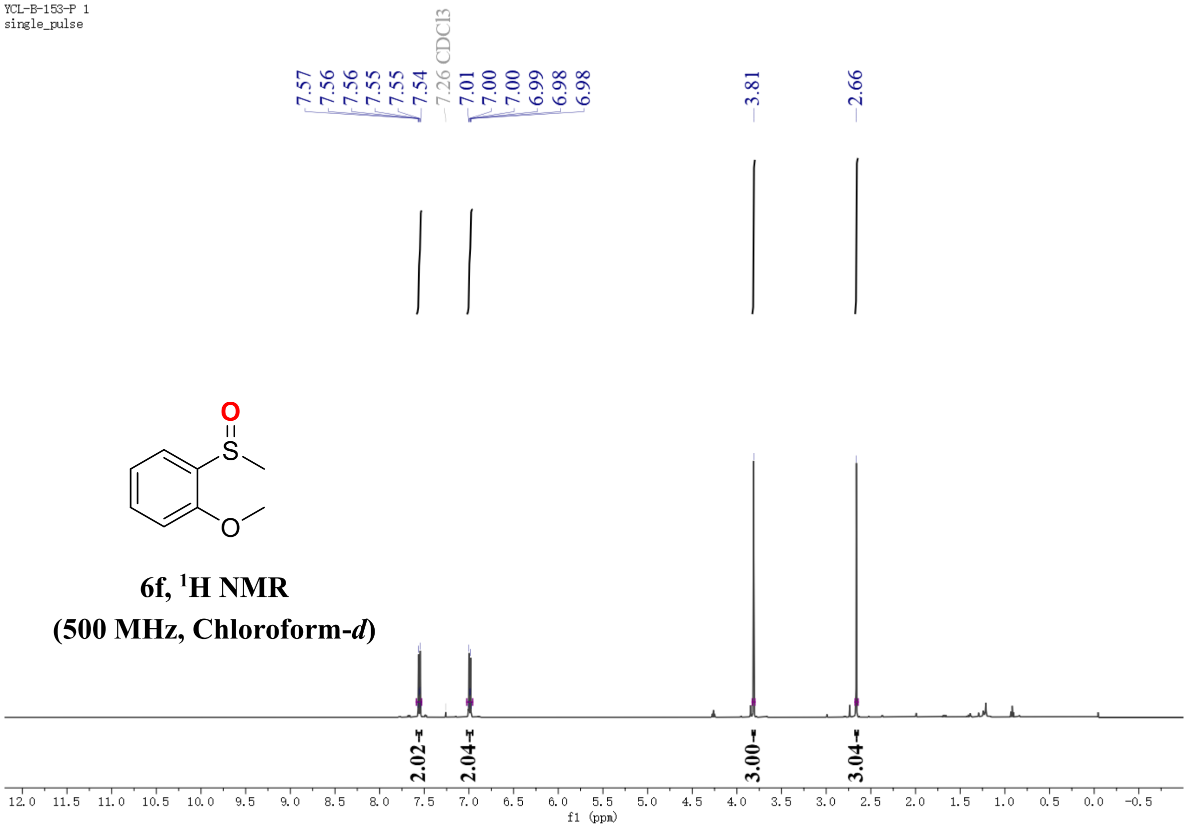

6f, ^{1}H NMR
(500 MHz, Chloroform-*d*)

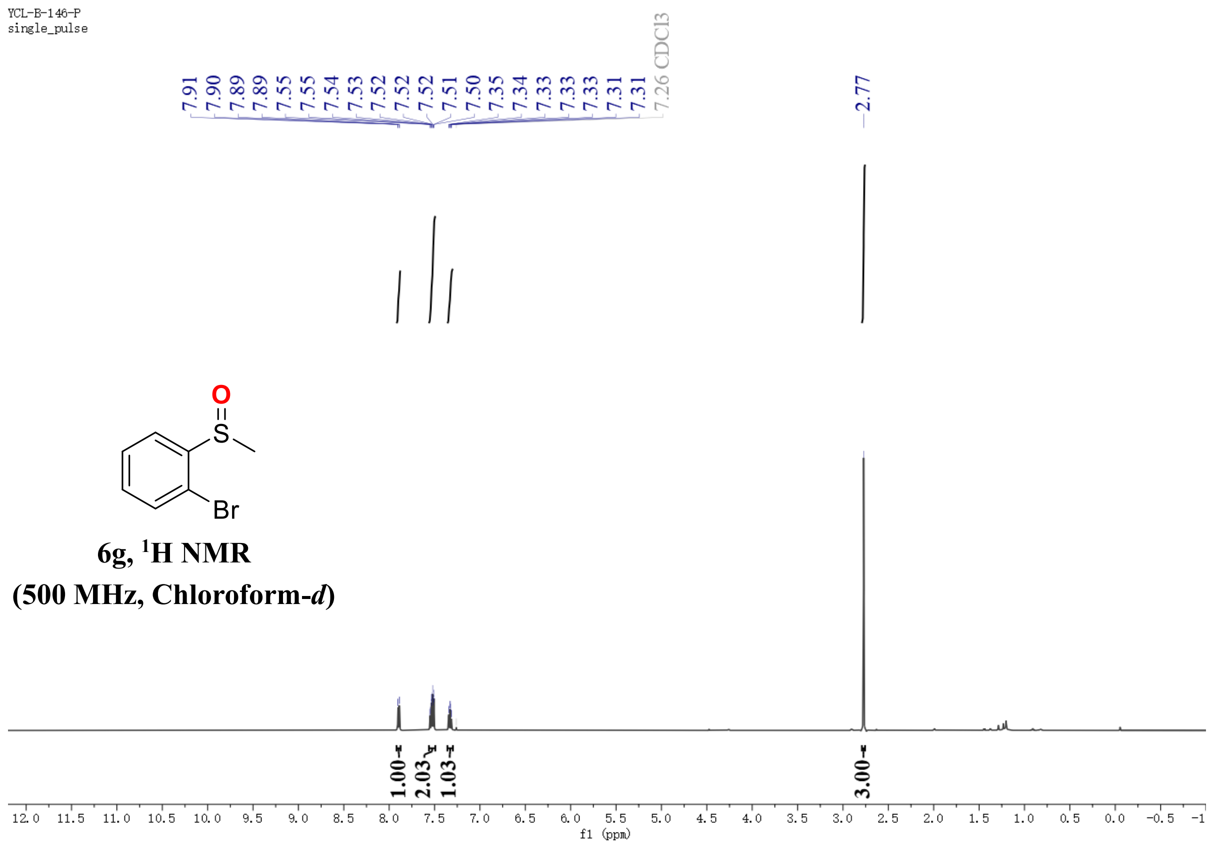

6g, ^{1}H NMR
(500 MHz, Chloroform-*d*)

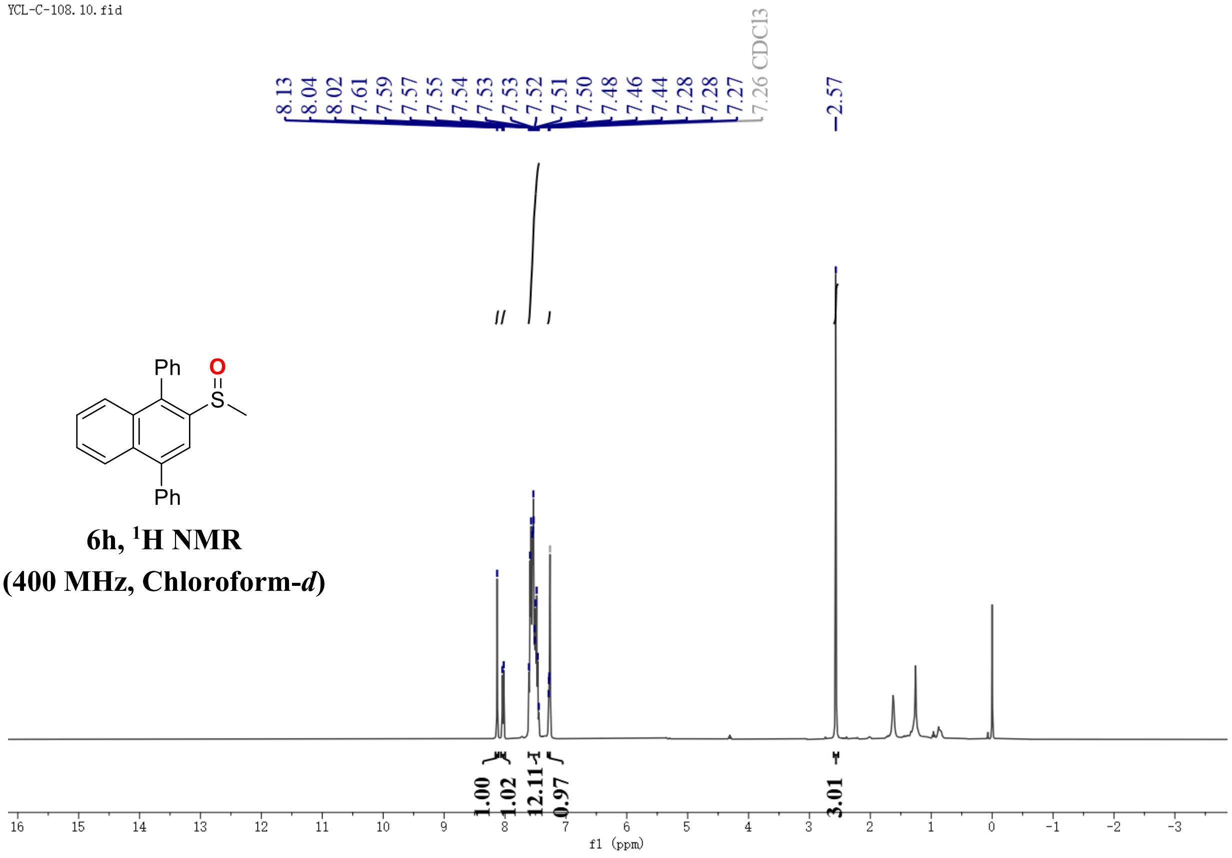

6h, ^{1}H NMR
(400 MHz, Chloroform-*d*)

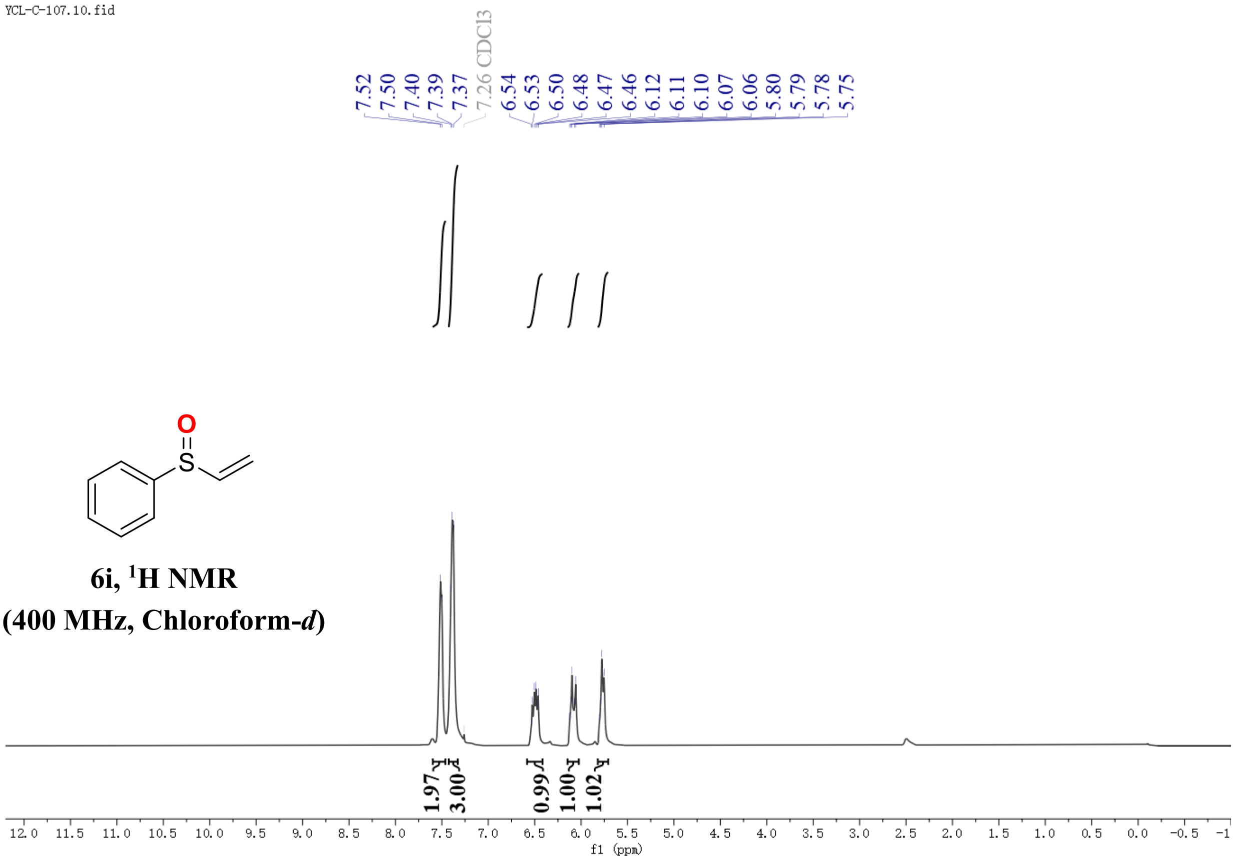

6i, ^{1}H NMR
(400 MHz, Chloroform-*d*)

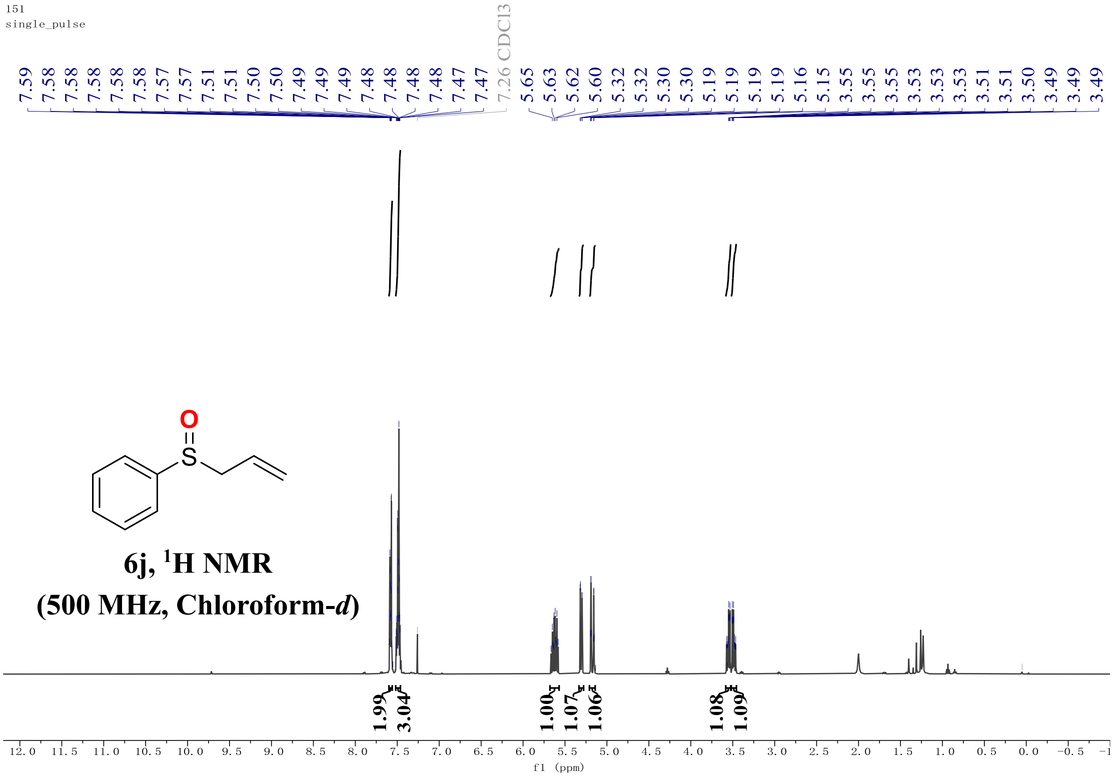

6j, ^{1}H NMR
(500 MHz, Chloroform-*d*)

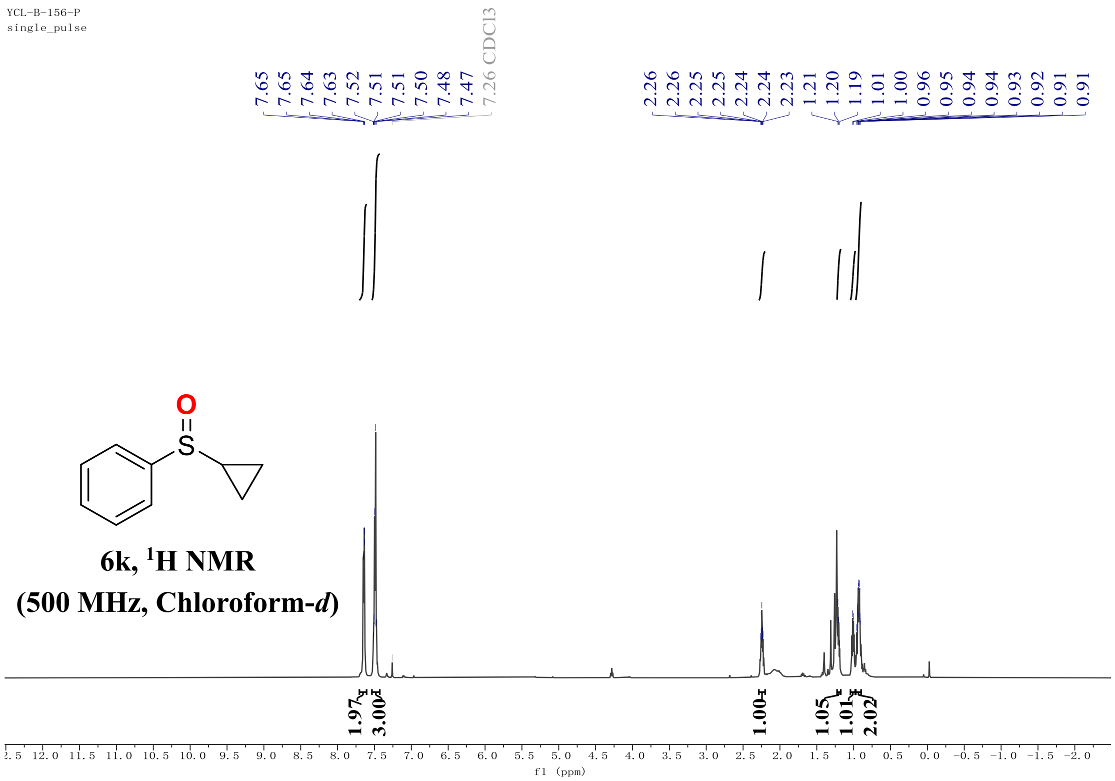
YCL-B-156-P
single_pulse
7.65
7.65
7.64
7.63
7.52
7.51
7.51
7.50
7.48
7.47
7.26 CDCl3
2.26
2.26
2.25
2.25
2.24
2.24
2.23
1.21
1.20
1.19
1.01
1.00
0.96
0.95
0.94
0.94
0.93
0.92
0.91
0.91
O
S
6k, ¹H NMR
(500 MHz, Chloroform-d)
1.97
3.00
1.00
1.05
1.01
2.02
f1 (ppm)

SI-9 References

[S1] D. C. Akintayo, W. A. Munzeiwa, S. B. Jonnalagadda, B. Omondi, *Inorg. Chim. Acta.* **2022**, 532, 120715.

[S2] C. Yu, X. He, C. Gong, J. Li, K.-Y. Ye, *Inorg. Chem.* **2025**, *Inorg. Chem.* **2025**, 64, 6977-6986.

[S3] J. Song, Z. Zhang, S. Hu, T. Wu, T. Jiang, B. Han, *Green Chem.* **2009**, 11, 1031-1036.

[S4] Y.-M. Jiang, Y.-Y. Lin, L. Zhu, Y. Yu, Y. Li, K.-Y. Ye, *CCS Chem.* **2024**, 6, 2021-2030.

[S5] G.-F. Yang, Y. Yuan, Y. Tian, S.-Q. Zhang, X. Cui, B. Xia, G.-X. Li, Z. Tang, *J. Am. Chem. Soc.* **2023**, 145, 5439-5446.

[S6] C. K. Savile, V. P. Magloire, R. J. Kazlauskas, *J. Am. Chem. Soc.* **2005**, 127, 2104-2113.

[S7] L. Ma, L. Bai, Z. Yu, Q. Shen, *Chirality* **2022**, 34, 1191-1196.

[S8] Gaussian 16, Revision C.01, M. J. Frisch, G. W. Trucks, H. B. Schlegel, G. E. Scuseria, M. A. Robb, J. R. Cheeseman, G. Scalmani, V. Barone, G. A. Petersson, H. Nakatsuji, X. Li, M. Caricato, A. V. Marenich, J. Bloino, B. G. Janesko, R. Gomperts, B. Mennucci, H. P. Hratchian, J. V. Ortiz, A. F. Izmaylov, J. L. Sonnenberg, D. Williams-Young, F. Ding, F. Lipparini, F. Egidi, J. Goings, B. Peng, A. Petrone, T. Henderson, D. Ranasinghe, V. G. Zakrzewski, J. Gao, N. Rega, G. Zheng, W. Liang, M. Hada, M. Ehara, K. Toyota, R. Fukuda, J. Hasegawa, M. Ishida, T. Nakajima, Y. Honda, O. Kitao, H. Nakai, T. Vreven, K. Throssell, J. A. Montgomery, Jr., J. E. Peralta, F. Ogliaro, M. J. Bearpark, J. J. Heyd, E. N. Brothers, K. N. Kudin, V. N. Staroverov, T. A. Keith, R. Kobayashi, J. Normand, K. Raghavachari, A. P. Rendell, J. C. Burant, S. S. Iyengar, J. Tomasi, M. Cossi, J. M. Millam, M. Klene, C. Adamo, R. Cammi, J. W. Ochterski, R. L. Martin, K. Morokuma, O. Farkas, J. B. Foresman, D. J. Fox, *Gaussian, Inc., Wallingford CT*, **2016**.

[S9] (a) R. Ditchfield, W. J. Hehre, J. A. Pople, *J. Chem. Phys.* **1971**, 54, 724-728; (b) W. J. Hehre, R. Ditchfield, J. A. Pople, *J. Chem. Phys.* **1972**, 56, 2257-2261; (c) P. C. Hariharan, J. A. Pople, *Theoret.*

Chim. Acta. **1973**, 28, 213-222.

[S10] S. Grimme, J. Antony, S. Ehrlich, H. Krieg, *J. Chem. Phys.* **2010**, 132, 154104.

[S11] S. Grimme, S. Ehrlich, L. Goerigk, *J. Comput. Chem.* **2011**, 32, 1456–1465.

[S12] (a) W. Florian, A. Reinhart, *Phys. Chem. Chem. Phys.* **2005**, 7, 3297. (b) D. Rappoport, F. Furche, *J. Chem. Phys.* 2010, 133, 134105.

[S13] GaussView, Version 6, R. Dennington, T. A. Keith, J. M. Millam, *Semichem Inc., Shawnee Mission, KS,* **2016**.

[S14] T. Lu, F. Chen, *J. Comput. Chem.* **2012**, 33, 580-592.

[S15] T. Lu, *J. Chem. Phys.* **2024**, 161, 082503.

[S16] J. Zhang, T. Lu, *Phys. Chem. Chem. Phys.* **2021**, 23, 20323-20328.

[S17] M. Ding, H.-L. Jiang, *ACS Catal.* **2018**, 8, 3194-3201.

[S18] Y. Gu, B. A. Anjali, S. Yoon, Y. Choe, Y. G. Chung, D.-W. Park, *J. Mater. Chem. A* **2022**,10, 10051-10061.

[S19] X.-J. Bai, X.-Y. Lu, R. Ju, H. Chen, L. Shao, X. Zhai, Y.-N. Li, F.-Q. Fan, Y. Fu, W. Qi, *Angew. Chem. Int. Ed.* **2021**, 60, 701-705.

[S20] K. Liu, S. Jiao, H. Zhao, F. Cao, D. Ma, *Green Chem.* **2021**, 23, 1766-1771.

[S21] M. Pander, M. Janeta, W. Bury, *ACS Appl. Mater. Interfaces* **2021**, 13, 8344-8352.

[S22] N. Qiao, X.-Y. Xin, W.-M. Wang, Z.-L. Wu, J.-Z. Cui, *Dalton Trans.* **2023**, 52, 10725-10736.

[S23] X. Zhang, C. Li, T. Hu, *ACS Sustainable Chem. Eng.* **2023**, 11, 17837-17848.

[S24] X. Zhang, X. Wang, C. Li, L. Fan, T. Hu, *Sep. Purif. Technol.* **2025**, 354, 128926.

[S25] Z. Gao, L. Liang, X. Zhang, P. Xu, J. Sun, *ACS Appl. Mater. Interfaces* **2021**, 13, 61334–61345

[S26] J. Lyu, X. Zhang, P. Li, X. Wang, C. T. Buru, P. Bai, X. Guo, O. K. Farha, *Chem. Mater.* **2019**, 31, 4166-4172.

[S27] Y. B. N. Tran, P. T. K. Nguyen, Q. T. Luong, K. D. Nguyen, *Inorg. Chem.* **2020**, 59, 16747-

16759.

[S28] Q. Zhai, Y. Ren, H. Wang, C. Liu, Z. Li, H. Jiang, *Dalton Trans.* **2024**, 53, 5836-5843.

[S29] H.-X. Liu, Z.-J. Zhou, L. Xie, C. Liu, L. Cai, X.-P. Wu, T.-F. Liu, *Angew. Chem. Int. Ed.* **2024**, 63, e202411508.

[S30] L.-Q. Wei, B.-H. Ye, *ACS Appl. Mater. Interfaces* **2019**, 11, 41448-41457.

[S31] Z. Wang, W.-F. Liang, X. He, J. Li, K.-Y. Ye, *Adv. Synth. Catal.* **2025**, 367, e202401025.

[S32] B.-G. Cai, H. Mao, K. Wang, J. Xuan, *Org. Chem. Front.* **2025**,12, 641-648.
